# Differential Sensitivity of Target Genes to Translational Repression by miR-17~92

**DOI:** 10.1371/journal.pgen.1006623

**Published:** 2017-02-27

**Authors:** Hyun Yong Jin, Hiroyo Oda, Pengda Chen, Chao Yang, Xiaojuan Zhou, Seung Goo Kang, Elizabeth Valentine, Jennifer M. Kefauver, Lujian Liao, Yaoyang Zhang, Alicia Gonzalez-Martin, Jovan Shepherd, Gareth J. Morgan, Tony S. Mondala, Steven R. Head, Pyeung-Hyeun Kim, Nengming Xiao, Guo Fu, Wen-Hsien Liu, Jiahuai Han, James R. Williamson, Changchun Xiao

**Affiliations:** 1 Department of Immunology and Microbial Science, The Scripps Research Institute, La Jolla, California, United States of America; 2 Kellogg School of Science and Technology, The Scripps Research Institute, La Jolla, California, United States of America; 3 State Key Laboratory of Cellular Stress Biology, Innovation Center for Cell Signaling Network, School of Life Sciences, Xiamen University, Xiamen, Fujian, China; 4 Division of Biomedical Convergence/Institute of Bioscience & Biotechnology, College of Biomedical Science, Kangwon National University, Chuncheon, Republic of Korea; 5 Department of Integrative Structural and Computational Biology, The Scripps Research Institute, La Jolla, California, United States of America; 6 Shanghai Key Laboratory of Regulatory Biology, Shanghai Key Laboratory of Brain Functional Genomics (Ministry of Education), School of Life Sciences, East China Normal University, Shanghai, China; 7 Interdisciplinary Research Center on Biology and Chemistry, Shanghai Institute of Organic Chemistry, Chinese Academy of Sciences, Shanghai, China; 8 Department of Molecular and Experimental Medicine, The Scripps Research Institute, La Jolla, California, United States of America; 9 Next Generation Sequencing Core, The Scripps Research Institute, La Jolla, California, United States of America; 10 Department of Molecular Bioscience/Institute of Bioscience & Biotechnology, College of Biomedical Science, Kangwon National University, Chuncheon, Republic of Korea; University of California Berkeley, UNITED STATES

## Abstract

MicroRNAs (miRNAs) are thought to exert their functions by modulating the expression of hundreds of target genes and each to a small degree, but it remains unclear how small changes in hundreds of target genes are translated into the specific function of a miRNA. Here, we conducted an integrated analysis of transcriptome and translatome of primary B cells from mutant mice expressing miR-17~92 at three different levels to address this issue. We found that target genes exhibit differential sensitivity to miRNA suppression and that only a small fraction of target genes are actually suppressed by a given concentration of miRNA under physiological conditions. Transgenic expression and deletion of the same miRNA gene regulate largely distinct sets of target genes. miR-17~92 controls target gene expression mainly through translational repression and 5’UTR plays an important role in regulating target gene sensitivity to miRNA suppression. These findings provide molecular insights into a model in which miRNAs exert their specific functions through a small number of key target genes.

## Author summary

MicroRNAs (miRNAs) are small RNAs encoded by our genome. Each miRNA binds hundreds of target mRNAs and performs specific functions. It is thought that miRNAs exert their function by reducing the expression of all these target genes and each to a small degree. However, these target genes often have very diverse functions. It has been unclear how small changes in hundreds of target genes with diverse functions are translated into the specific function of a miRNA. Here we take advantage of recent technical advances to globally examine the mRNA and protein levels of 868 target genes regulated by miR-17~92, the first oncogenic miRNA, in mutant mice with transgenic overexpression or deletion of this miRNA gene. We show that miR-17~92 regulates target gene expression mainly at the protein level, with little effect on mRNA. Surprisingly, only a small fraction of target genes respond to miR-17~92 expression changes. Further studies show that the sensitivity of target genes to miR-17~92 is determined by a non-coding region of target mRNA. Our findings demonstrate that not every target gene is equal, and suggest that the function of a miRNA is mediated by a small number of key target genes.

## Introduction

MicroRNAs (miRNAs) are endogenously encoded single stranded RNAs of about 22 nucleotides (nts) in length. They suppress target gene expression by translational repression and promoting mRNA degradation. The relative contribution of these two modes of action to miRNA regulation of its target gene expression is a matter of ongoing debate [1–3]. It was initially thought that animal miRNAs repress the protein output of target genes without significantly effecting mRNA levels [4, 5]. Subsequent genetic studies in *C*. *elegans* and zebrafish showed that miRNAs also promote the degradation of their target mRNAs [6, 7]. To reveal the global effect of miRNA on target gene mRNA and protein levels, a series of genome-wide studies applied microarray, RNA-seq, proteomics, and ribosome profiling to mammalian cell lines transiently transfected with miRNA mimics or inhibitors or primary cells from miRNA mutant mice. Two early studies showed significant correlations between the mRNA and protein levels of miRNA target genes, as well as widespread target mRNA degradation [8, 9]. This was followed up by a study concluding that mammalian miRNAs predominantly act to decrease target mRNA levels [10]. However, other studies that employed the same experimental approach, namely transient transfection of miRNA mimics or inhibitors into *in vitro* cultured mammalian cell lines, came to an opposite conclusion. These studies showed that miRNAs affect the expression of most target genes through translational inhibition [11, 12]. Subsequent studies employing temporal dissection of miRNA action seemed to have resolved this discrepancy by showing that translational repression precedes target mRNA deadenylation and decay [13–18]. This order of events can be interpreted either as evidence that mRNA decay is a consequence of translational repression [17, 19], or as reflection of the kinetic differences between these two mechanisms that operate independently from each other [20]. In line with the latter interpretation, analyses performed either in cultured cells or *in vitro* extracts showed that miRNA-mediated translational repression can occur in the absence of target mRNA deadenylation and decay [19, 21–27]. Therefore, it remains an unanswered question whether mRNA degradation is always the end result of miRNA targeting and whether miRNA-mediated translational repression and target mRNA degradation are molecularly coupled under physiological conditions [1, 28, 29].

In contrast to the efforts to search for a unified mechanism of miRNA action, studies of individual miRNA-target mRNA interactions in miRNA mutant mice are painting a rather different picture. A recent survey of literature focused on studies in which target gene mRNA and protein levels were measured concurrently in primary cells and tissues from mutant mice with genetic ablation or transgenic expression of individual miRNA genes [2]. This survey analyzed a total of 159 miRNA-target mRNA interactions in 77 strains of miRNA mutant mice. Among them, 48% target genes are predominantly regulated by translational repression, 29% are regulated mainly by mRNA degradation, and 23% are regulated by both. This heterogeneity in miRNA mechanisms of action has been increasingly recognized as more and more miRNA mutant mice are generated and analyzed, but what determines the dominant mode of miRNA action remains unclear. An interesting finding of this survey is that most target genes identified in developing cells or tissues are regulated by mRNA degradation, whereas target genes identified in terminally differentiated cells tend to be regulated at the translational level. It is conceivable that mRNA degradation gets rid of target mRNA in a non-reversible manner and provides an efficient way for cell fate determination, while translational repression is immediate, transient and reversible, which is more suitable for differentiated cells to respond to environmental stimuli [2]. Indeed, previous studies have shown that miRNA regulation of target gene translation can occur in a rapid and reversible manner under various stress conditions [30, 31]. These studies highlight the importance of cellular context in determining the dominant mode of miRNA action.

The mode of action can also be miRNA-dependent. Transcriptome analysis of mouse liver showed that miR-122 and let-7 cause significant target mRNA degradation, whereas miR-21 has little impact on its target gene mRNA levels [32]. Another study of primary cells from miRNA mutant mice showed that miR-155 in B cells and miR-223 in neutrophils cause significant target mRNA degradation, while miR-150 in B cells and miR-21 in neutrophils have absolutely no effect on their target mRNA abundance [14]. Considering the cellular context- and miRNA-dependency, it is essential to investigate miRNA mechanisms of action in the cellular contexts where miRNA of interest performs its physiological or pathological functions.

Another controversial issue in miRNA research is about how miRNAs achieve their specific functions. On one hand, bioinformatic analysis and experimental target gene identification using the recently established PAR-CLIP and HITS-CLIP methods often find hundreds of target genes for a miRNA [33–36]. Proteomic analysis of mammalian cell lines transiently transfected with miRNA mimics showed that a miRNA regulates the protein output of hundreds of target genes, and that the effect on each target gene is often moderate [8, 9]. These studies led to the conclusion that miRNAs exert their functions by modulating the expression of hundreds of target genes and each to a small degree [37, 38]. However, when the hundreds of target genes regulated by a miRNA are closely examined, they often fall into a broad spectrum of functional categories [36, 39, 40]. How small changes in hundreds of target genes with diverse functions are translated into specific phenotypic outcomes has been a conceptual conundrum. On the other hand, recent genetic studies demonstrated that mutation of miRNA binding sites in a single target gene can phenocopy miRNA deficiency in a cell context-dependent manner in both mice and worms [41–43]. These results provide strong support to the key target gene model, which postulates that the function of a miRNA is often mediated by a small number of key target genes in a given cellular context [44]. We speculated that the discrepancy between these two types of studies regarding how miRNAs exert their specific functions stems from the transient transfection approach, which may not recapitulate the actions of endogenous miRNAs under physiological conditions [2]. Recent studies showed that transient transfection of miRNA mimics into *in vitro* cultured cell lines led to increase of mature miRNAs to supraphysiological levels, appearance of high molecular weight RNA species, frequent mutation of guide strands of miRNA mimics, accumulation of unnatural passenger strands of miRNA mimics, and non-specific alterations in gene expression [45–47]. These findings call into question the physiological relevance of previous studies employing the transient transfection approach to investigate the functions and mechanisms of miRNAs. As increasing numbers of animals harboring gain- and loss-of function mutations for individual miRNA genes are being generated [2, 48], primary cells from these miRNA mutant animals are better systems for studying miRNA mechanisms of action under physiological conditions.

In this study, we investigated miRNA mechanism of action in lymphocytes by conducting an integrated analysis of the transcriptomes and translatomes of primary B cells from miR-17~92 transgenic and knockout mice. The miR-17~92 family consists of three miRNA clusters: miR-17~92, miR-106a~363, and miR-106b~25 (**S1 Fig**). Together, these three clusters contain 15 miRNA stem-loops that give rise to 13 distinct mature miRNAs. They fall into four miRNA subfamilies (miR-17, miR-18, miR-19, and miR-92 subfamilies), with members in each subfamily sharing the same seed sequence. Germline knockout of miR-17~92 family in mice is incompatible with life [49]. These miRNAs are essential for the development of lung, heart, central nervous system, fetal liver, and B lymphocytes [49]. B cell-specific deletion of the miR-17~92 family (CD19-Cre;miR-17~92^fl/fl^;miR-106a~363^-/-^;miR-106b~25^-/-^, termed TKO mice) severely impaired antibody responses, while B cell-specific miR-17~92 transgenic (TG) mice develop lymphomas with high penetrance [40]. This conditional transgene and knock-out strategy bypasses developmental defects caused by dysregulated miR-17~92 expression during the early stages of B cell development [50, 51]. We have now performed a comprehensive molecular analysis of primary B cells expressing miR-17~92 miRNAs at three different levels (TKO, WT and TG). In this cellular context, we found that target genes exhibit differential sensitivity to miRNA suppression, and that only a small fraction of target genes are actually suppressed by a given concentration of miRNA. Absolute quantification of miRNA and miRNA binding site revealed there are more miRNA binding sites than miRNA molecules so that only a small fraction of binding sites are occupied by miRNA molecules at a given time. Moreover, miR-17~92 controls key target gene expression mainly through translational repression and 5’UTR plays an important role in regulating target gene sensitivity to miRNA suppression. These findings provide mechanistic insights into the functional specificity of miRNAs.

## Results

### miR-17~92 regulates functional target gene expression predominantly at the protein level

**Fig 1:**
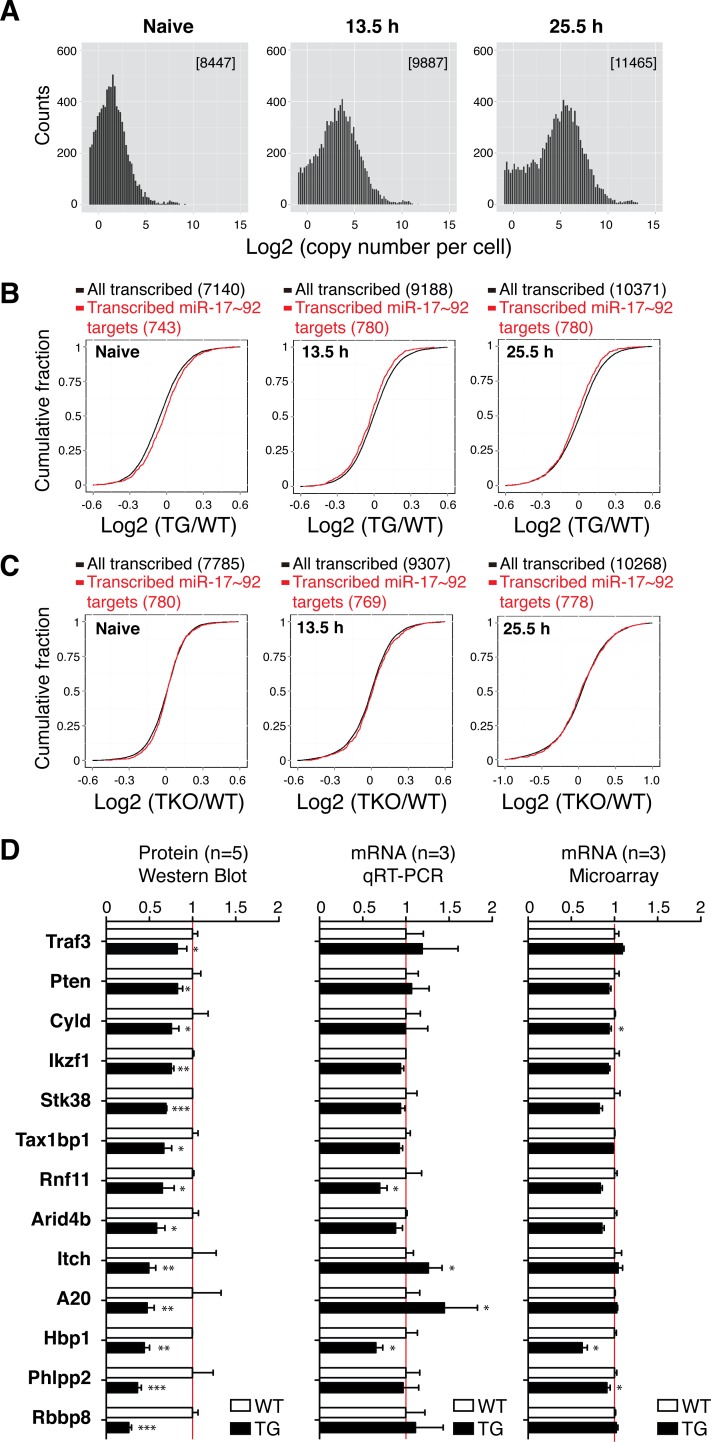
The impact of miR-17~92 on target gene mRNA and protein levels. (**A**) The distribution of mRNA abundance in naïve and activated B cells as determined by ERCC-RNA-seq analysis. Numbers in parenthesis represent the number of unique genes significantly transcribed (greater than 0.5 copy per cell). Y-axis (counts) indicates the number of genes of a given abundance (X-axis, bin size = 0.2). (**B,C**) Microarray analysis of TKO, WT, and TG B cells. Numbers in parenthesis indicate the numbers of transcribed genes and transcribed miR-17~92 targets analyzed by microarray. (**D**) The protein and mRNA levels of 13 target genes showing reduced protein levels in 25.5h activated TG B cells as determined by Immunoblot (n = 5). mRNA levels were determined by qRT-PCR and microarray (n = 3). Target gene expression levels were normalized to β-Actin, and their relative expression in WT naïve B cells was arbitrarily set as 1.0.

We have previously identified 868 target genes harboring a total of 1139 miR-17~92 binding sites conserved in human and mouse (termed miR-17~92 targets) by PAR-CLIP analysis of B cells [40]. This list contains most of miR-17~92 target genes validated in previous studies. We investigated the effect of transgenic miR-17~92 expression and complete deletion of the miR-17~92 family on the mRNA levels of these target genes during B cell activation. We first generated a complete list of significantly expressed mRNAs and their absolute molecule numbers by RNA-seq analysis of WT B cells spiked with a known quantity of ERCC control (ERCC-RNA-seq, **S2A and S3 Figs**) [52]. This analysis showed that 8,000 (naïve B cells) to 11,000 (B cells activated for 25.5h) genes are transcribed in B cells at greater than 0.5 copy per cell (termed transcribed genes), with median copy numbers of 2.6 (naïve), 10 (13.5h), and 31 (25.5h) (**Fig 1A and S1 Table**). This general transcriptional upregulation is essential for activation-induced cell growth and proliferation. Consistent with previous reports, the abundance of significantly expressed mRNAs spans three to four orders of magnitude (**S3A Fig**), with 1 RPKM roughly corresponding to 1 copy per cell (**S3B Fig**) [53, 54]. The transcribed genes included 85% (743 in naïve B cells) to 90% (780 in 25.5h activated B cells) of miR-17~92 target genes (termed transcribed targets).

We next performed microarray analysis of TKO, WT and TG B cells before and after activation (**S2B Fig**), focusing on the transcribed targets (**Fig 1B and 1C**). The time frame used in this study covered both the induction and termination phases of major signaling pathways involved in B cell activation (**S4A Fig**). We confirmed that miR-17~92 expression in TG B cells was 3 fold higher than in WT B cells, and was completely absent in TKO B cells (**S4B and S4C Fig).** When miR-17~92 target genes were analyzed, neither transgenic miR-17~92 expression nor deletion of miR-17~92 family caused significant global changes in their mRNA levels throughout B cell activation (**Fig 1B and 1C and S2 Table**). Analysis of target genes regulated by individual members of the miR-17~92 cluster came to the same conclusion (**S5A and S5B Fig**).

We next examined the effect of miR-17~92 on predicted target genes with the highest context++ scores based on the most recent TargetScan 7.0 algorithm (**S3 Table)** [55]. We selected 128 top target genes for each miRNA subfamily in this cluster, and analyzed the mRNA levels of these transcribed in B cells at greater than 0.5 copy per cell (**S5C and S5D Fig**). In a previous study, transfection of chemically synthesized miRNA mimics into HCT116 cells led to an average reduction of 19% in the mRNA levels of target genes with the same context scores [55]. During B cell activation, there was indeed an inverse correlation between the expression levels of miR-17~92 and these target gene mRNAs at all time points examined, but the average change in target mRNA levels was only 3.7% in TG and 6.6% in TKO B cells (**S5C and S5D Fig**). This rather modest global effect of miR-17~92 on the mRNA levels of its target genes is consistent with the results from previous studies performing transcriptome analysis of T cells, B lymphoma cells, and embryonic heart and tail bud with genetic ablation of either the whole miR-17~92 cluster or its individual members [51, 56–58]. We speculate that these subtle changes in target gene mRNA levels may not explain the dramatic phenotypes observed in TG and TKO mice. Moreover, most of the small number of target genes that show greater than 1.4-fold changes in mRNA levels have not been previously implicated in lymphoma development, cell survival and proliferation, and are unlikely to mediate the functions of miR-17~92 in B cells (**S2 Table**). Therefore, we investigated the possibility that miR-17~92 regulates the expression of functionally relevant target genes mainly at the protein level.

We compiled a list of 63 miR-17~92 target genes, which were either validated in previous studies [59–64], or are novel but functionally relevant to B cell lymphoma development or B cell immune responses (**S4 Table**). Among these 63 targets, we were able to detect and quantify 47 by immunoblot, while the other 16 were discarded due to poor antibody quality (**S6 Fig)**. Only 13 of the 47 target genes showed significant reduction in protein levels in TG B cells (**S7A Fig**), including several inhibitors of the PI3K (*Pten* and *Phlpp2*) and NF-κB (*Tnfaip3/A20*, *Itch*, *Rnf11*, *Tax1bp1*, *Cyld*, *and Traf3)* pathways previously implicated in miR-17~92-driven B cell lymphoma development [40, 65], and five additional tumor suppressor genes (*Hbp1*, *Stk38*, *Arid4b*, *Rbbp8* and *Ikzf1*) [66–69]. Among the other 34 targets, there were no significant changes in protein levels for 25 targets, time- or isoform-dependent changes for 3 targets, and increased protein levels for 6 targets in TG B cells (**S7B and S7C Fig**). We further examined the 13 target genes that exhibited reduced protein levels in TG B cells, focusing on the relative contribution of translational repression and mRNA degradation (**Fig 1D**). We validated the microarray data by qRT-PCR. All of them are regulated either exclusively or significantly at the protein level, except *Hbp1*, which is regulated mainly at the mRNA level (**Fig 1D**). We also measured the protein levels of 16 genes that control translation initiation and elongation in a global manner and completely lack miR-17~92 binding sites in their mRNAs [70]. As shown in **S8 Fig,** none of them was significantly downregulated in TG B cells. Taken together, these results demonstrated that the global impact of miR-17~92 on its target gene mRNA levels is subtle, that only a subset of functionally relevant target genes are suppressed by transgenic miR-17~92 expression, that miR-17~92-mediated suppression occurs predominantly at the protein level, and that this suppression is not caused by an altered translational environment in TG B cells.

### Target genes exhibit different sensitivity to changes in miR-17~92 expression

We next assessed the impact of miR-17~92 expression on target genes using ribosome profiling (**S2C Fig**). This technology directly captures genome-wide maps of protein synthesis (the translatome) by quantifying ribosome density on each mRNA with high resolution and depth, but does not measure post-translational changes in gene expression [71, 72]. We quantified ribosome footprints of 8,271 mRNAs (termed translated genes) in TKO, WT and TG B cells after 25.5h of activation, corresponding to more than 70% of transcribed genes in these cells (**S5 Table**). The ribosome footprint abundance spans six orders of magnitude (**S9A Fig**), which is at least two orders of magnitude broader than that of mRNA abundance (**S3A Fig**), in agreement with previous global gene expression analysis in mammalian cells [73]. We confirmed that ribosome footprint abundance changes highly correlated with protein abundance changes as determined by immunoblot, therefore excluding significant contribution from post-translational regulatory mechanisms such as miRNA-dependent nascent polypeptide destruction (**S9B–S9E Fig**) [74].

Among the 780 transcribed miR-17~92 targets, 641 were detected by significant numbers of ribosome footprints (termed translated targets) (**S10A Fig**). Notably, only 123 (19.2% of translated targets) showed greater than 1.4-fold reduction in ribosome footprint abundance in TG B cells (termed ribo-downregulated TG targets), while only 80 (12.5% of translated targets) were de-repressed by 1.4 fold or more in TKO B cells (termed ribo-upregulated TKO targets) (**S10B and S10D Fig**). When the median values of translation changes were compared with these of mRNA changes, translation changes were dominant at the global level, in both TG and TKO B cells (**S10C and S10E Fig**). Therefore, only a small fraction of target genes respond to changes in miR-17~92 expression levels and miR-17~92 regulates its target gene expression mainly at the translational level.

**Fig 2:**
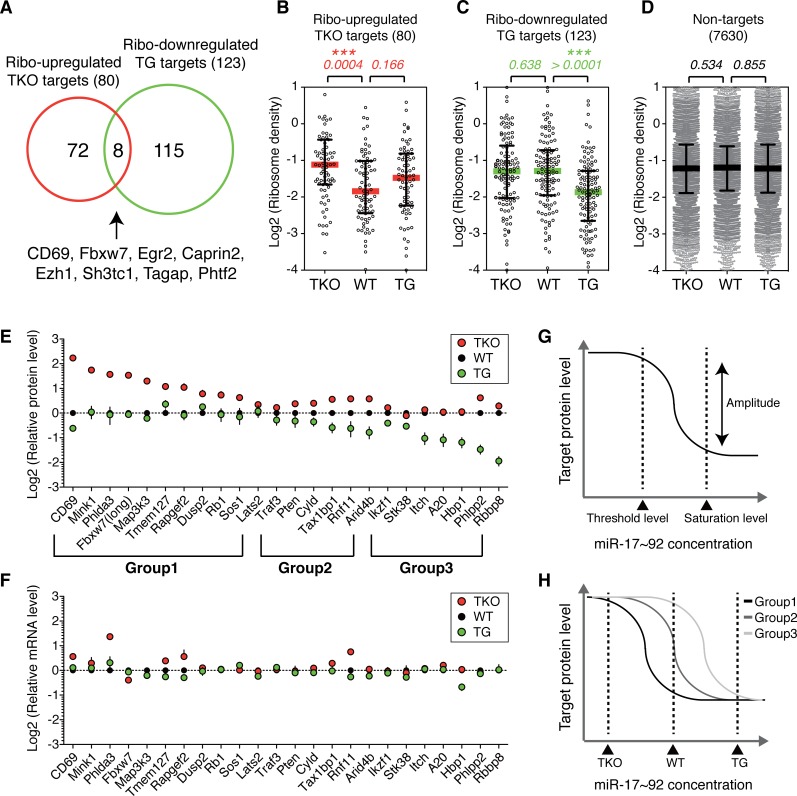
Target genes exhibit different sensitivity to miR-17~92 expression level changes. **(A)** Minimal overlap between ribo-upregulated TKO targets and ribo-downregulated TG targets. **(B-D)** The responses of ribosome density of ribo-upregulated TKO targets **(B)** and ribo-downregulated TG targets **(C)** to three miR-17~92 expression levels. Translated genes lacking miR-17~92 binding sites were used as control **(D)**. Colored bars indicate median values and error bars represent interquartile ranges. Each dot represents relative ribosome density of a unique gene. Numbers indicate p-values. **(E)** Different sensitivity of individual target genes to miR-17~92 expression level changes. Protein levels were determined by immunoblot and normalized to β-Actin (**S7 and S11 Figs**). Target gene protein levels in WT B cells were arbitrarily set as 1.0 (n≥4). Vertical lines indicate error bars. **(F)** Relative mRNA levels of individual target genes in TKO, WT and TG B cells as determined by microarray (n = 3). **(G)** A hypothetical curve depicting target gene protein level change as a function of miRNA concentration. For a miRNA-target mRNA interaction in a given cellular context, there are a threshold level and a saturation level of miRNA concentration. miRNA suppresses target gene expression in a dose-dependent manner when miRNA concentration is between the threshold and saturation levels. Suppression does not occur when miRNA concentration is below the threshold level, while suppression reaches a maximal when miRNA concentration is above the saturation level. **(H)** The hypothetical response curves of group1, group2 and group3 target genes to miR-17~92 expression level changes. Note that the difference in amplitude for individual target genes is not depicted in this graph.

We compared the list of target genes de-repressed by miR-17~92 family miRNA deletion (ribo-upregulated TKO targets) with those suppressed by transgenic miR-17~92 overexpression (ribo-downregulated TG targets). To our surprise, these two lists overlapped by only 8 genes, including four previously validated targets (*CD69*, *Fbxw7*, *Egr2*, *and Caprin2*) (**Fig 2A**) [75–80]. When ribosome profiling data of TKO, WT, and TG B cells were analyzed together, it became clear that ribo-upregulated TKO targets as a group showed significant reduction in ribosome density when miR-17~92 family miRNA expression increased from almost zero in TKO B cells to WT levels, but did not show further reduction when miRNA expression increased from WT to TG levels (**Fig 2B and S6 Table**). In the same analysis, the ribosome density of ribo-downregulated TG targets did not exhibit any significant changes between TKO and WT B cells, but showed significant reduction when miR-17~92 expression increased from WT to TG levels (**Fig 2C and S6 Table**). Translated genes lacking miR-17~92 binding sites were used as negative control, whose ribosome density showed no significant alterations in B cells expressing miR-17~92 at three different levels (**Fig 2D**).

The differential responses of target genes to three different levels of miR-17~92 expression in TKO, WT and TG B cells were confirmed by immunoblot analysis of individual target genes (**S11 Fig**). We first examined TKO B cells for their expression of the 13 targets suppressed in TG B cells (**S7A Fig**). Six of them (*Phlpp2*, *Rnf11*, *Arid4b*, *Tax1bp1*, *Cyld and Pten*) showed significant de-repression in protein levels, but the degree of de-repression was relatively small (1.2–1.5 fold), while the expression of the other seven was not altered (**S11A and S11B Fig**). In contrast, among the 34 targets that were not suppressed in TG B cells (**S7B and S7C Fig**), 10 showed significant increase in ribosome footprint abundance in TKO B cells and belonged to ribo-upregulated TKO targets (*Mink1*, *Phlda3*, *Fbxw7*, *Map3k3*, *Tmem127*, *Rapgef2*, *Dusp2*, *Rb1*, *Sos1 and Lats2*) (**S6 Table**). This was further confirmed by immunoblot analysis of TKO B cells, which showed up to 3.3-fold increases in protein levels of these genes (**S11C and S11D Fig**). When the relative protein levels of these 23 targets in TKO, WT, and TG B cells were plotted together, it became obvious that different targets exhibit different sensitivity to changes in miR-17~92 expression levels (**Fig 2E**). Ten targets (termed group 1 targets) were suppressed when miR-17~92 expression increased from TKO to WT levels, but showed little suppression in TG B cells. Seven targets (termed group 3 targets) were suppressed when miR-17~92 expression increased from WT to TG levels, but showed only marginal de-repression in TKO B cells. The other six targets (termed group 2 targets) showed suppression when miR-17~92 expression increased from WT to TG levels, and were de-repressed in TKO B cells (**Fig 2E**). In contrast to the significant changes in their protein levels, the mRNA levels for most of them remain the same in TKO, WT, and TG B cells, regardless of target groups (**Fig 2F**). We next performed reporter assays in wild type B cells to investigate whether miR-17~92 exerts its effect on these target genes through its cognate binding sites on target mRNAs. As shown in **S12 Fig,** mutation of miR-17~92 binding sites led to increased activity of a luciferase gene fused to target gene 3’UTRs, therefore demonstrating direct regulation of these target genes by miR-17~92 in B cells.

Based on these results, we propose the following model of differential sensitivity of target genes to miRNA suppression. For a miRNA-target mRNA interaction, there is a threshold level and a saturation level of miRNA concentration (**Fig 2G**). Target gene expression is suppressed by miRNA in a dose-dependent manner when miRNA concentration is between these two levels. Below the threshold level, target gene expression cannot be suppressed by miRNA. Above the saturation level, target gene expression cannot be further suppressed by increasing concentration of miRNA. The maximal difference in target gene protein levels (termed amplitude) is reached when miRNA concentration increases from the threshold level to the saturation level. Different target genes exhibit different threshold level, saturation level, and amplitude in their responses to the same miRNA (or miRNA cluster) (**Fig 2H**). The differences in threshold and saturation levels underlie the different sensitivity of group 1, 2, 3 target genes to changes in miR-17~92 expression levels, while the differences in amplitude explain the various degrees of suppression or de-repression in TG and TKO B cells, respectively (**Fig 2E**).

### There are more miRNA binding sites than miRNA molecules

**Fig 3:**
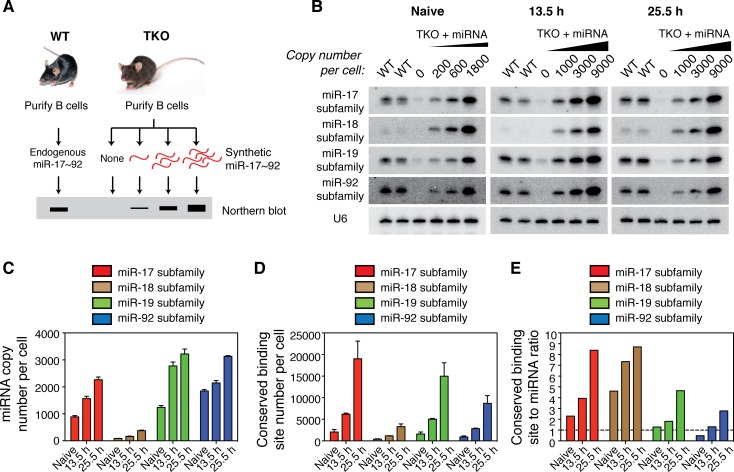
Quantification of miR-17~92 miRNAs and binding sites in primary B cells. (**A,B**) Quantitative Northern blot to determine miR-17~92 miRNA copy numbers. Indicated amounts of synthetic miR-17, miR-18a, miR-19b and miR-92 were added to naïve and activated TKO B cells before RNA extraction. The copy numbers of each miRNA subfamily were determined by Northern blot comparing WT B cells and TKO B cells with graded amounts of spike-in synthetic miRNAs, using a mixture of probes corresponding to all members of a miRNA subfamily (also see **S7 Table**). Naïve B cells were activated with LPS and IL-4 for indicated amounts of time (h, hour). (**C-E**) Summary of miR-17~92 family miRNA copy numbers (**C**), conserved miR-17~92 family miRNA binding sites (**D**) (also see **S8 Table**), and ratios of conserved miR-17~92 family miRNA binding sites to miRNAs (**E**) in naïve and activated B cells.

A prediction of this model is that not all miRNA binding sites are occupied by miRNA. Therefore, it is likely that there are less miRNA molecules than miRNA binding sites in WT B cells. To test this, we determined the copy numbers of miR-17~92 miRNA molecules and miR-17~92 binding sites present in B cells during activation. The miRNA molecule numbers in WT B cells were determined by quantitative Northern blot analysis of WT B cells and TKO B cells spiked with graded amounts of chemically synthesized mature miR-17~92 family miRNAs (**Fig 3A–3C and S7 Table**). By combining the mRNA molecule numbers determined by ERCC-RNA-seq (**S1A Fig**) and conserved miR-17~92 binding sites determined by PAR-CLIP [40], we calculated the number of conserved miR-17~92 binding sites in a B cell (**Fig 3D and S8 Table**). Our calculation showed that each naïve B cell expresses 900–1,800 molecules of miR-17, miR-19, and miR-92 subfamily miRNAs, and 80 molecules of miR-18 subfamily miRNAs (**Fig 3B and 3C and S7 Table**). The ratios between conserved miR-17~92 binding sites and miRNA molecules range from 0.5 (miR-92 subfamily) to 4.6 (miR-18 subfamily) in naïve B cells (**Fig 3E**). Upon activation, both miR-17~92 miRNAs and their target mRNAs are up-regulated (**Fig 3C and 3D**), but the fold increase of the latter outpaces the former, thereby increasing the ratios between conserved binding sites and miRNA molecules to 2.8 (miR-92 family) and 8.7 (miR-18 family) in 25.5h activated B cells (**Fig 3E**). Moreover, the PAR-CLIP analysis identified 2.4-fold more non-conserved binding sites than conserved ones [40]. Previous studies showed that non-conserved binding sites can also be occupied by RISC [36]. Taking non-conserved binding sites into account, potential miR-17~92 binding sites outnumber miRNA molecules even further, by as much as 20-fold. These estimations are consistent with results from previous studies measuring the molecule numbers of miRNAs and their binding site numbers on target mRNAs in hepatocytes and stem cells [81, 82]. Therefore, we conclude that only a fraction of potential binding sites are occupied by miR-17~92 miRNAs at any given time.

### miRNAs reduce ribosome occupancy on a fraction of target mRNA molecules

**Fig 4:**
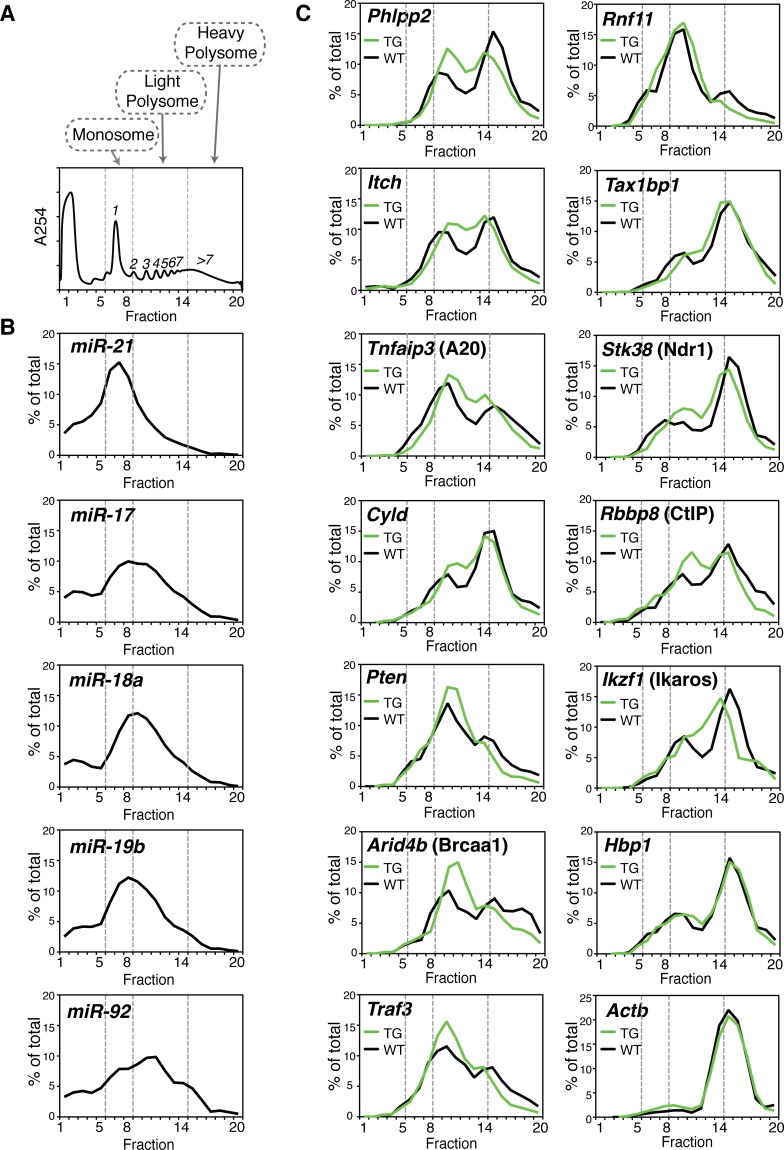
Transgenic miR-17~92 expression shifts target mRNAs from heavy to light polysomes. (**A**) A representative polysome profile of activated B cells, from two biological replicates for each genotype. Numbers inside the graph indicate the number of ribosomes associated with mRNA. (**B**) Distribution of miR-21 and miR-17~92 in the sucrose gradient in WT B cells. (**C**) Distribution of miR-17~92 target mRNAs in the sucrose gradient in WT and TG B cells. β-Actin mRNA (*Actb*) is enriched in heavy polysome fractions and is used as an internal control.

We next investigated how miRNAs regulate target gene translation using polysome profiling (**S2D Fig**) [23, 83]. While ribosome profiling measures ribosome footprint abundance, which is a sum of mRNA abundance and translation rate [72], polysome profiling directly measures the number of ribosome associated with a mRNA molecule, independent of mRNA expression level (**S13 Fig**) [84]. We first confirmed that miRNA gene mutations had little impact on the global polysome profile (**S14 Fig**). We then investigated the distribution of individual miRNAs and mRNAs in the sucrose gradient. miR-21, one of a few miRNAs enriched in monosome fractions [32, 85], and highly abundant in B cells [86], was used as control. In contrast to miR-21, miR-17~92 miRNAs were mainly associated with light polysomes (**Fig 4A and 4B**). This suggests that miR-17~92 miRNAs are predominantly associated with target mRNAs undergoing slow translation. Next we measured the distribution of target mRNAs in the sucrose gradient. While the β-Actin mRNA (*Actb*) was enriched in heavy polysome fractions, mRNAs of all validated miR-17~92 target genes exhibited a bimodal distribution (**Fig 4C**). The first peak was located at fractions 10–11, corresponding to mRNAs associated with 3–4 ribosomes, while the second peak was located at fractions 14–16, corresponding to mRNAs associated with more than 7 ribosomes (**Fig 4C**). Our quantification of miR-17~92 family miRNA molecules and their potential binding sites on target mRNAs in B cells suggested that only a fraction of target mRNA molecules are occupied by these miRNAs (**Fig 3E**). In addition, the distribution of miR-17~92 family miRNAs largely overlapped with the first peak of their target mRNAs (**Fig 4B and 4C**). Taken together, these results suggest that target mRNAs are compartmentalized: target mRNAs in the first peak are associated with miR-17~92 family miRNAs and undergo slow translation, while target mRNAs in the second peak are largely free of miR-17~92 family miRNAs and undergo more active translation. Transgenic miR-17~92 expression shifted a fraction of target mRNAs from the second peak into the first peak (**Fig 4C**), thereby reducing the overall translation rate and protein output (**S7A Fig**). Consistent with the previous observations that miR-17~92 regulation of *Hbp1* occurs mainly at the mRNA level (**Fig 1D and S9E Fig**), the distribution of the *Hbp1* mRNA in the sucrose gradient showed little change (**Fig 4C**).

We conducted the same analyses for another well-studied lymphocyte-specific miRNA, miR-155 [87], to see whether our observation is a general phenomenon. This included absolute quantification of miR-155 and its binding sites in WT B cells and ribosome profiling analysis of miR-155-deficient (155KO) B cells. Our results showed that there are 7-fold more conserved miR-155 binding sites than miR-155 molecules (**S15A Fig**), that miR-155 was enriched in light polysome fractions (**S15B Fig**), and that deletion of miR-155 caused a significant shift of mRNAs of previously validated target genes *(Aicda*, *Sfpi1*, *Jarid2 and Peli1)* from light to heavy polysomes (**S15C Fig**) [88–91].

To independently confirm these results, we took an un-biased approach to assess changes of target mRNA distribution in the sucrose gradient (Poly-RNA-seq, **S16A Fig**). We performed RNA-seq analysis of total RNA purified from polysome fractions 10–11 and 14–16, corresponding to the first and second peaks of target mRNA distribution in the sucrose gradient, and calculated the relative abundance of target mRNA of interest in these two peaks in B cells from mice of different genotypes. This analysis produced results consistent with polysome profiling analysis, showing that miR-17~92 target mRNAs were enriched in fractions 10–11 while depleted in fractions 14–16 in TG B cells, and miR-155 target mRNAs were depleted in fraction 10–11 while enriched in fractions 14–16 in miR-155 KO B cells (**S16B and S16C Fig**). Taken together, our polysome profiling analysis of individual target gene mRNAs demonstrated that miRNAs suppress target gene expression by reducing ribosome occupancy on a fraction of target mRNA molecules.

### Ribosome accumulation in the 5’UTR correlates with translational repression

**Fig 5:**
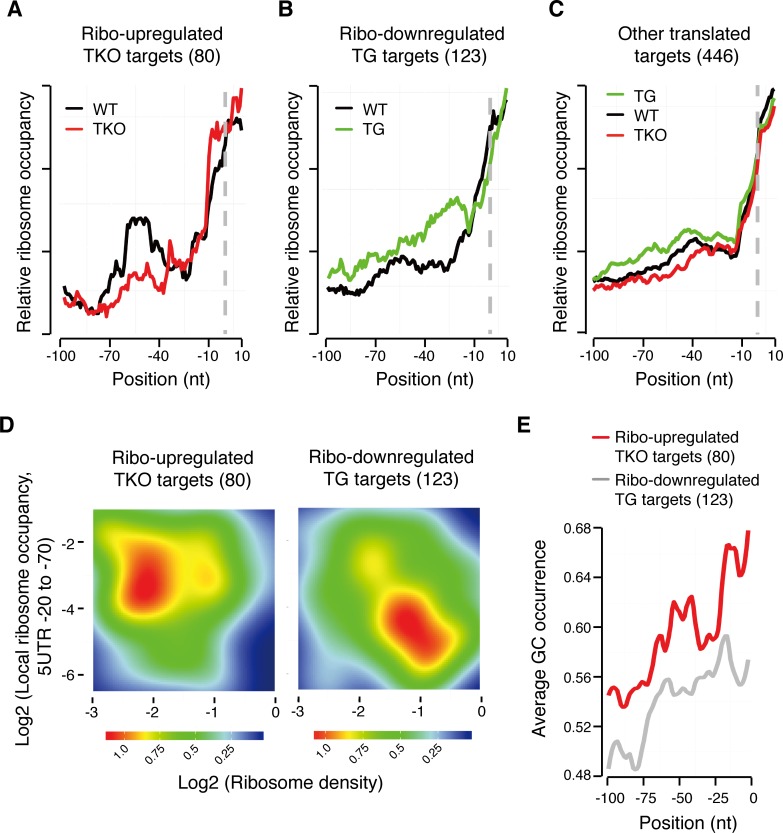
Ribosome accumulation in 5’UTR correlates with translational repression of target genes. (**A-C**) Ribosome accumulation in 5’UTRs of ribo-upregulated TKO targets in WT B cells (**A**), ribo-downregulated TG targets in TG B cells (**B**), but not in 5’UTRs of other targets (**C**). Ribosome occupancy in 5’UTR was normalized to the overall ribosome footprint abundance of the same gene [96]. The first nucleotide of start codon is set as position 0 (grey dashed line). (**D**) Inverse correlation between ribosome occupancy in 5’UTR and the overall ribosome density on target mRNA in WT B cells. (**E**) High GC content in 5’UTRs of ribo-upregulated TKO targets.

We sought to understand what determines target gene sensitivity to miRNA-mediated translational repression. While the contribution of seed types and other *cis*-factors has been extensively investigated in the cellular contexts in which miRNAs predominantly act to decrease target mRNA levels [8, 92], the factors that regulate miRNA-mediated translational repression remain largely unknown. We systematically investigated the length of 5’UTR, coding region (CDS) and 3’UTR, numbers of conserved miR-17~92 binding sites, enrichment of specific seed types, and locations of binding sites. We found mRNAs with miR-17~92 binding sites tend to have longer 5’UTR, CDS, and 3’UTR, but their length did not predict target gene sensitivity. None of the other features correlates with target gene sensitivity globally (**S17 Fig**). As our results showed that miR-17~92 suppresses target gene expression mainly through translational repression, we then focused on molecular features implicated in translational regulation [93]. Ribosome footprint distribution analysis showed that there were ribosome footprints in 5’UTRs of miR-17~92 target genes, though their abundance was lower than ribosome footprint abundance in CDS (**S18 Fig**). A close examination revealed a significant accumulation of ribosome footprints in 5’UTRs of ribo-upregulated TKO targets in WT B cells as compared to TKO B cells (**Fig 5A**). This suggested that miR-17~92 represses translation initiation of these target genes through their 5’UTRs (**See** **discussion**). Consistently, ribosome footprint abundance in 5’UTRs of ribo-downregulated TG targets was increased when miR-17~92 expression increased from WT to TG levels (**Fig 5B**), while other non-responsive target genes did not show significant changes in ribosome footprint abundance in their 5’UTRs in TKO, WT or TG B cells (**Fig 5C**). Moreover, local ribosome occupancy in 5’UTRs inversely correlated with overall ribosome density, which is a good indicator of translation rate and protein output. This suggests a role of ribosome hindrance in 5’UTR in suppressing translation initiation (**Fig 5D**). We searched the 5’UTRs of ribo-upregulated TKO targets for potential enrichment of specific sequence motifs but did not find any. Instead, we found high GC content in these 5’UTRs, and the position of the GC content peak correlated with the position of the ribosome footprint peak (**Fig 5A and 5E**). The translation efficiency of mammalian mRNAs is highly sensitive to GC content of 5’UTR, as high GC content often indicates the presence of secondary structures. A previous study showed that an increase in 5’UTR GC content from 52% to 62% led to a 2-fold decrease in translation efficiency [94]. In line with this, recent bioinformatic analyses implied that local structures in 5’UTRs contribute to efficient miRNA-mediated gene regulation *via* translational repression [19, 95]. Moreover, our reporter assay experiments confirmed direct regulation of group 1 targets by miR-17~92 in wild type B cells, but the degree of de-repression in reporter activity caused by binding site mutation was often less than the degree of de-repression in target gene protein levels in TKO B cells (**Fig 2E and S12 Fig**). This suggests that cis-elements beyond miRNA binding sites in 3’UTRs contribute to the amplitude of target gene regulation. Taken together, we surmised that ribosome hindrance mediated by secondary structures in 5’UTRs contribute to target gene sensitivity to miRNA suppression at the translation initiation stage.

### 5’UTR regulates target gene sensitivity to miRNA suppression

**Fig 6:**
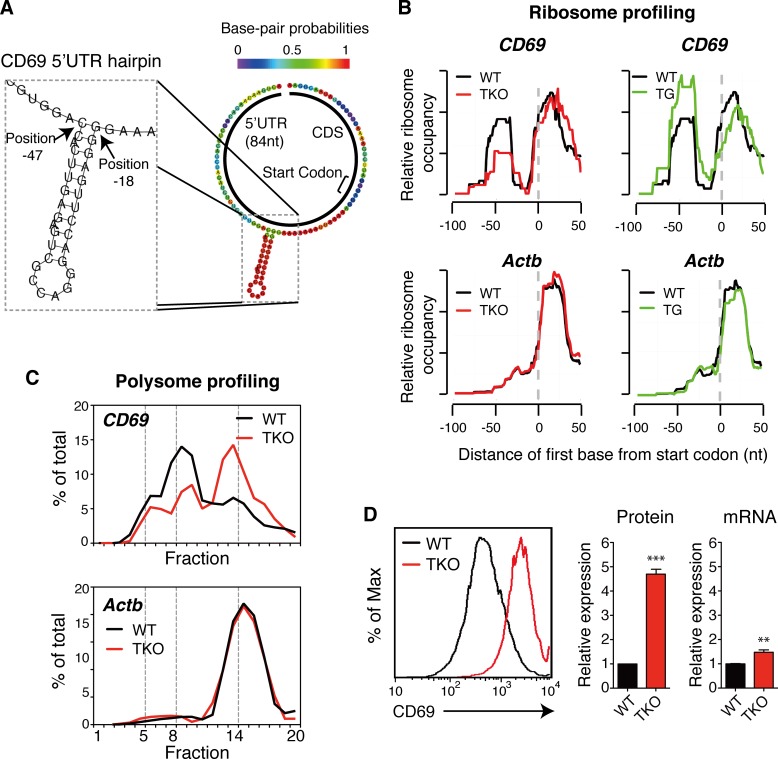
Secondary structures in 5’UTR correlate with translational repression of target genes. (**A**) A hairpin structure in the *CD69* 5’UTR. (**B**) Ribosome accumulation in *CD69* 5’UTR correlated with miR-17~92 family miRNA expression levels. Note that the hairpin structure co-localizes with the ribosome footprint peak in the *CD69* 5’UTR. *Actb* was used as control. (**C**) Deletion of the miR-17~92 family miRNAs shifted *CD69* mRNA from light to heavy polysomes. (**D**) Increased *CD69* expression in TKO B cells was mainly due to translation de-repression. Experiments in **B-D** were performed with 25.5h activated B cells.

We explored this idea further by focusing on *CD69*, the most sensitive target gene among the 24 validated by immunoblot (**Fig 2E**). *CD69* has a relatively short 5’UTR (84nt), which harbors no internal ribosome entry sites (IRES) or 18s rRNA binding regions that may enhance cap-independent translation [97–99]. Instead, there are two sub-optimal start codons (AUC and GUG) and a potential hairpin [100, 101] (**Fig 6A and S19 Fig**). Consistent with the global analysis of ribo-upregulated TKO targets (**Fig 5A**), there was an accumulation of ribosome footprints in *CD69* 5’UTR in WT B cells (**Fig 6B**). The ribosome footprint is 31 nt long, corresponding to the width of a single ribosome. Interestingly, the ribosome footprint overlaps with the two sub-optimal start codons and the 5’ arm of the putative hairpin, while its abundance shows positive correlation with miR-17~92 expression levels and negative correlation with *CD69* expression (**Figs 6B and 2E**). We hypothesized that the two sub-optimal start codons and the hairpin work together to slow down translational initiation, thereby rendering *CD69* mRNA sensitive to translational repression. Indeed, polysome profiling analysis confirmed that miR-17~92 represses *CD69* expression at the translation level (**Fig 6C**), and deletion of the miR-17~92 family miRNAs led to a 4.5-fold increase in cell surface expression of *CD69* in TKO B cells, with only marginal effect on its mRNA level (**Fig 6D**).

**Fig 7:**
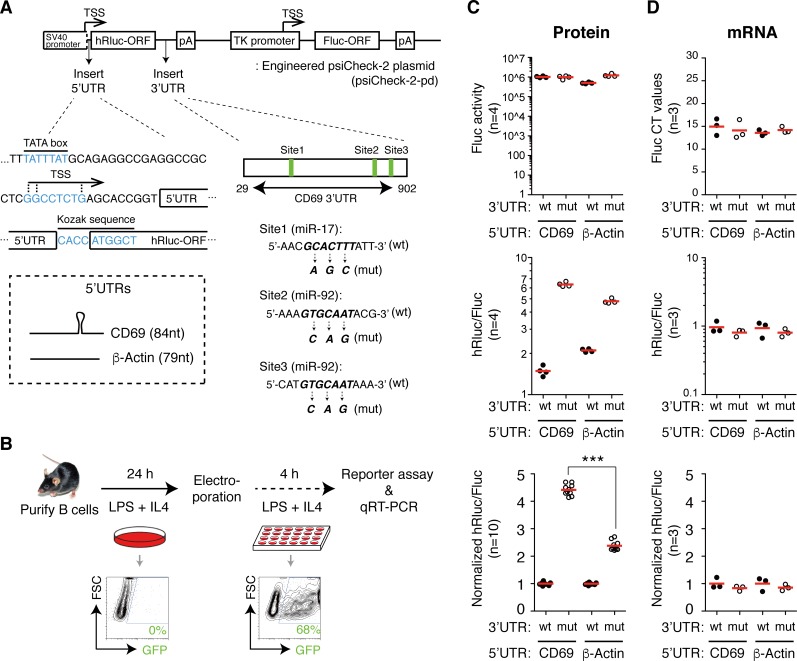
Regulation of target gene sensitivity to miRNA suppression by 5’UTR. (**A**) An engineered psiCheck2 vector (psiCheck-2-pd) for investigating the effect of 5’UTR and 3’UTR on reporter gene expression. TSS, transcription start site. (**B**) Experimental scheme of reporter assays in primary B cells. FACS plots show electroporation efficiency using a GFP-expressing plasmid. (**C,D**) Dual luciferase reporter assay to determine the effect of 5’UTR and 3’UTR on the reporter gene protein (luciferase activity) (**C**) and mRNA (qRT-PCR) levels (**D**). Closed and open circles indicate reporters with wild-type (wt) and mutated (mut) *CD69* 3’UTR, respectively. A comparison of renilla luciferase activity normalized to firefly luciferase activity (hRluc/Fluc) between psiCheck-2-pd containing mut and wt *CD69* 3’UTR reveals the sensitivity of the renilla luciferase mRNA (hRluc) to miR-17~92-mediated suppression. Results of normalized hRlcu/Fluc (n = 10) are from three independent experiments. Each experiment contained 3–4 replicates.

We investigated the functional contribution of *CD69* 5’UTR to its regulation by miR-17~92 using a modified form of the dual luciferase reporter psiCheck-2 (**Fig 7A**) [102]. In this plasmid (termed psiCheck-2-pd), the firefly luciferase gene (Fluc) controlled by the human thymidine kinase (TK) gene promoter is used as an internal reference for transfection efficiency. We placed the *CD69* 3’UTR downstream of the renilla luciferase gene (hRluc). The wild type *CD69* 3’UTR (wt) contains three binding sites for miR-17~92 miRNAs (one for miR-17 subfamily and two for miR-92 subfamily). We introduced 3nt mutations into these binding sites to abolish their interactions with miR-17~92 miRNAs to generate a mutated form of *CD69* 3’UTR (mut). A comparison of the renilla/firefly luciferase activity ratio (hRluc/Fluc) between psiCheck-2-pd containing wt and mut *CD69* 3’UTR should reveal the sensitivity of the renilla luciferase mRNA to miR-17~92-mediated suppression.

To examine the role of *CD69* 5’UTR in regulating translation rate and sensitivity to miRNA suppression, we inserted *CD69* 5’UTR between the transcription start site of (TSS) of the SV40 promoter and the Kozak sequence of the renilla luciferase gene (**Fig 7A**). The β-*Actin* 5’UTR contains no obvious secondary structures, exhibited minimal accumulation of ribosome footprints in TKO, WT, and TG B cells (**Fig 6B**), and was used as a control. We performed dual-luciferase reporter assays in *in vitro* activated WT B cells to closely imitate the experimental conditions of ribosome profiling and polysome profiling (**Fig 7B**). As expected, the firefly luciferase activity remained as a constant (**Fig 7C**). The renilla luciferase reporter containing *CD69* 5’UTR showed a 4.4 fold de-repression when miR-17~92 binding sites in its 3’UTR were mutated, very similar to the fold de-repression of the endogenous *CD69* gene in TKO B cells (**Figs 6D and 7C**). Replacing *CD69* 5’UTR with β-*Actin* 5’UTR significantly reduced the sensitivity of the renilla luciferase reporter gene to miR-17~92 suppression (**Fig 7C**). qRT-PCR analysis of renilla and firefly luciferase mRNAs showed that the ratio between these two mRNAs was not affected by changes in 5’UTR or 3’UTR, excluding any substantial contributions from mRNA changes (**Fig 7D**). We next performed similar reporter assays in TG, WT, and TKO B cells, using the psiCheck-2-pd reporter with wild type *CD69* 5’UTR and 3’UTR. Consistent with CD69 expression in B cells of these three genotypes (**Fig 2E**), the expression of renilla luciferase was more sensitive to miR-17~92 depletion than overexpression (**S20A Fig**).

To understand the functional contribution of the putative hairpin and two sub-optimal start codons in *CD69* 5’UTR to the sensitivity of *CD69* mRNA to miR-17~92 suppression, we deleted the left arm of the hairpin (ΔHP) or mutated these two sub-optimal start codons (Mut-uORF), and performed reporter assays in WT B cells. Deletion of the left arm of the hairpin reduced the sensitivity of renilla luciferase to miR-17~92 suppression, but no significant effect was observed for mutating the two sub-optimal start codons (**S20B Fig**). Taken together, these results demonstrate that structural components in 5’UTR play an important role in regulating the sensitivity of target mRNA to translational repression by miRNAs.

## Discussion

### Target gene sensitivity and the key target gene model

This study provides mechanistic insights into the functional specificity of miRNAs and the key target gene model, which postulates that miRNAs exert their specific functions by suppressing the expression of a small number of key target genes [44]. Our findings, together with previously published studies [41–43], suggest that key target genes emerge from a pool of hundreds of target genes *via* multiple mechanisms. That is, there are mechanisms that regulate miRNA binding to target mRNAs, the consequences of miRNA binding, and cellular responses to reduced target gene protein levels (**S21 Fig**).

First, there are more binding sites than miRNA molecules and only a fraction of binding sites are occupied by miRNA-containing RISC complexes at any given time. Target mRNAs often associate with RNA-binding proteins (RBPs) and exhibit certain secondary and tertiary structures, which interfere with the recruitment of RISC and result in differential accessibility and affinity to miRNA [30, 103–106]. When hundreds of target mRNA species compete for a limited amount of miRNA molecules, binding sites with easy accessibility and high affinity are preferentially occupied. Increasing the cellular concentration of miRNA molecules leads to saturation of the most favorable binding sites and occupation of additional binding sites with lower accessibility and affinity. Consistent with our view, a previous study demonstrated that target accessibility is a critical determinant of miRNA-mediated translational repression in the cellular context where miRNAs do not cause target mRNA degradation [107]. Therefore, the accessibility and affinity of binding sites, as well as the presence of competing target mRNA species, establish the threshold and saturation levels of miRNA for a given target mRNA (**Fig 2G**).

Second, miRNA binding does not necessarily warrant functional consequence. There are mechanisms that determine whether miRNA binding leads to changes in target gene protein levels. This study shows that 5’UTR is a part of the mechanisms regulating target gene sensitivity to miRNA suppression at the translation initiation stage.

Third, there are mechanisms that regulate cellular responses to changes in target gene protein levels. We speculate that changes in the protein levels of many target genes brought about by a miRNA are functionally inconsequential, as shown by many examples of genetic mutant mice with no observable phenotypes [108]. Nevertheless, there are a small number of target genes that are functionally sensitive to reduced protein levels in a given cellular context, as documented by the pathologies arising from haploinsufficiency [109–111]. These dosage sensitive target genes likely serve as critical mediators of miRNA functions and are the key target genes in that particular cellular context (**S21 Fig**).

### A quantitative perspective of the key target gene model

How many key targets are there to mediate the function of a miRNA in a given cellular context? Our global analysis of miR-17~92 target genes in primary B cells provide insights into this question. Among the 868 experimentally identified targets with conserved miR-17~92 binding sites [40], 780 are significantly transcribed and 641 are significantly translated. When the cutoff is set at 1.4 fold change in ribosome footprint abundance, only 80 of them are suppressed by the WT levels of miR-17~92 and qualify as responsive targets, amounting to 9% of experimentally identified targets. As discussed above, it is likely that only a fraction of these 80 target genes are relevant for the function of miR-17~92 in B cells. Therefore, the number of key target genes is further reduced to a few percent of the 868 targets. For miRNA genes encoding a single mature miRNA, which often has 100–200 putative target genes, this would translate into only a few key targets for a given cellular context (**S21 Fig**). Consistent with this estimation, recent genetic studies showed that mutation of miRNA binding sites in a single target gene phenocopied defects caused by miRNA deficiency in a cell context-dependent manner, demonstrating that individual miRNA-target mRNA interactions can play critical roles in mediating the function of miRNAs in animals [41–43].

### 5’UTR contributes to translational repression by miRNAs

For most mRNAs, translation initiation occurs by a cap-dependent scanning mechanism, which requires the binding of the trimeric complex eIF4F (comprised of eIF4E, eIF4A, and eIF4G) to the m^7^G cap structure, followed by recruitment of the preinitiation complex (PIC) and scanning of PIC to the first AUG codon positioned within a good context [112] (**S22A Fig**). The secondary structures in their 5’UTRs play important roles in regulating translation initiation [113]. Scanning through these secondary structures require additional factors and ATP, and this requirement depends on the position and stability of secondary structures [114, 115]. RNA helicases such as eIF4A are required for unwinding these secondary structures and for facilitating the scanning of PIC [116].

Recent studies suggested that miRNAs require eIF4As to regulate translation of their target mRNAs [19, 117, 118]. While two studies demonstrated miRNAs repress target gene translation by facilitating dissociation of eIF4As from target mRNAs [117, 118], a third one proposed they repress target mRNAs by recruiting eIF4AII [19]. Even though the detailed molecular mechanisms by which eIF4As mediate miRNA function are contradictory in these reports, the requirement of eIF4As in miRNA function during PIC scanning is consistent with other studies that utilized reporter constructs whose translational initiation bypasses the PIC scanning process. These reporter genes were immune to miRNA-mediated repression, suggesting that miRNA repression takes place during PIC scanning [23, 119]. It should also be noted that other studies demonstrated that miRISC and the CCR4-NOT complex can silence target mRNA in an eIF4A-independent manner, suggesting the eIF4A dependency can be context-specific [120].

Our study suggests that the most sensitive targets (such as ribo-upregulated TKO targets) contain more structured 5’UTRs. In the absence of miR-17~92 family miRNAs (in TKO B cells), eIF4As or other RNA helicases facilitate the unwinding of these secondary structures, allowing PIC to scan through and to initiate translation. In the presence of WT levels of miR-17~92 family miRNAs, RISC complexes are recruited to these target mRNAs through their cognate binding sites in the 3’UTRs, and dissociate RNA helicases from the 5’UTRs. This results in stabilization of secondary structures and accumulation of PIC (and ribosome footprints in ribosome profiling experiments) in the 5’UTRs, repression of translation initiation, and a reduction in protein output (**S22B Fig**). When miR-17~92 expression is further increased to the TG levels, less sensitive targets (such as ribo-downregulated TG targets) that do not respond to WT levels of miR-17~92 become responsive at this higher level. Our reporter assays demonstrate that specific structural components in 5’UTR indeed regulate miRNA-mediated translational repression, but the detailed molecular interactions between miRNA, 5’UTR, and the translation initiation machinery warrant future investigation.

### Functional implications of differential target gene sensitivity

Our findings also provide a straightforward explanation for the recent observations that deletion and overexpression of the same miRNA gene can lead to unrelated phenotypes [121, 122]. As a representative example, early studies have shown that overexpression of members of the miR-34 family miRNAs has potent tumor suppressor function downstream of p53 [121]. However, mice carrying target deletion of all miR-34 genes display normal p53 responses to a variety of cellular insults, including ionizing radiation and oncogenic stress [123]. Another study reported that mice deficient of all the six miRNAs in the miR-34/449 family exhibited postnatal mortality, infertility and strong respiratory dysfunction caused by defective mucociliary clearance, resulting from a significant decrease in cilia length and number [122]. Our study suggests that different functions of miR-34 family miRNAs in these overexpression and deletion studies can be explained by different sensitivity of target genes to miR-34 suppression. When these miRNAs are expressed at WT levels, target genes regulating cilia assembly (i.e. *Cp110*) are among the most sensitive and their expression is suppressed. Deletion of all miR-34/449 family genes results in de-repression of these genes and impaired cilia assembly [122]. When miR-34 family miRNAs are overexpressed at levels much higher than WT levels, another group of target genes, which are less sensitive and only respond to higher than WT levels of miR-34, are suppressed. This group contains positive regulators of cell cycle and DNA-damage responses (i.e. *Cdk4*, *Ccne2*, and *Met*), whose suppression bestows anti-tumor functions to miR-34 family miRNAs [121]. Therefore, different sensitivity of these two groups of target genes, one regulating cilia assembly and the other regulating cell cycle and DNA damage response, to miR-34 suppression underlies the different phenotypic consequences brought about by overexpression and deletion of this family of miRNAs.

### miRNA tips the balance between translationally active and inactive target mRNAs

A recent study investigating real-time translation of single mRNA molecules in live mammalian cells revealed surprising heterogeneity in the translation of individual mRNAs from the same gene within the same cell, including rapid and reversible transitions between translationally active and inactive states [124]. The same study showed that the long form 5’UTR of the *Emi1* gene, when placed upstream of a GFP reporter gene, caused a 40-fold reduction in the GFP protein level. While a great majority of GFP mRNAs containing the long form 5’UTR of the *Emi1* gene were strongly translationally repressed, a small subset of these mRNAs still escaped repression and underwent robust translation. These results suggest that cis-elements in the long form 5’UTR of the *Emi1* gene drastically shifted, but did not completely shut off, the GFP mRNAs from translationally active states into inactive states. This is quite similar to our polysome profiling analysis of miR-17~92 target genes in WT and TG B cells, which showed that transgenic miR-17~92 expression shifted only a fraction of its target mRNAs from rapid translation states into slow translation states. A previous study investigating the effect of endogenous Let-7 miRNA on a reporter target gene in HeLa cells came to similar conclusions [23]. The authors further proposed that the translationally repressed reporter mRNAs, as well as Let-7 miRNAs, are localized in processing bodies, a subcellular structure for mRNA storage or degradation. Considering our study together with these other studies, it is likely that miRNAs repress target gene expression by tipping the dynamic balance between translationally active and inactive states.

### Translational repression versus target mRNA degradation

Similar to the heterogeneity in the translation of individual mRNAs from the same gene within the same cell, emerging evidence suggests that mechanisms of miRNA action are also heterogeneous. A recent survey of studies investigating miRNA effect on functionally important target genes in 77 strains of miRNA mutant mice found that miRNA-target interaction can lead to translational repression, target mRNA degradation, or both [2]. It remains unclear what determines the relative contribution of these two modes of miRNA action to target gene suppression. Previous studies suggest that this could be both cellular context- and miRNA-dependent [1, 2, 125]. In this study, we showed that most functional target genes of miR-17~92 are suppressed at the translational level, but some target genes are suppressed by mRNA degradation, either completely or partially. This target gene-dependency became more clear when we applied the same transcriptome and translatome analyses to miR-155-deficient B cells. Our unpublished results showed that in the same cellular context, miR-155 suppresses its target gene expression by translational repression, mRNA degradation, or both, and this is completely target-gene dependent. Future investigation is warranted to identify cellular factors, as well as *cis*-elements in both miRNAs and target mRNAs, that determine the molecular consequence of individual miRNA-target mRNA interactions.

In summary, we conducted an integrated analysis of miR-17~92 family miRNAs, their target genes, and the functional consequences of these miRNA-target gene interactions in primary B cells expressing miR-17~92 family miRNAs at three different physiological levels. We present evidence showing that there are more binding sites than miRNA molecules, that target genes exhibit differential sensitivity to miRNA suppression, and that only a small fraction of target genes are actually suppressed by a given concentration of miRNA. Transgenic expression and deletion of the same miRNA gene regulate largely distinct sets of target genes. miR-17~92 regulates functional target gene expression mainly through translational repression in activated B cells and 5’UTR plays an important role in regulating target gene sensitivity to miRNA suppression. These findings provide mechanistic insights into the key target gene model in which the specific function of a miRNA is achieved by regulating a small number of key target genes.

## Materials and methods

### Ethics statement

All mice were used in accordance with guidelines from the Institutional Animal Care and Use Committees of The Scripps Research Institute and Xiamen University.

### Mice

The generation of miR-17~92 Tg (Jax stock 008517), miR-17~92^fl/fl^ (Jax stock 008458), miR-106a~363^-/-^ mice (Jax stock 008461), miR-106b~25^-/-^ mice (Jax stock 008460), CD19-Cre (Jax stock 006785) was previously reported [49, 126, 127]. MiR-17~92 Tg mice were crossed with CD19-Cre mice to generate miR-17~92 Tg/Tg;CD19Cre (TG) mice [40]. miR-17~92^fl/fl^ mice were crossed with miR-106a~363^-/-^ mice, miR-106b~25^-/-^ mice and CD19-Cre mice to generate miR-17~92^fl/fl^;miR-106a~363^-/-^;miR-106b~25^-/-^;CD19-Cre (TKO) mice. miR-155-/- were obtained from The Jackson Laboratory (Jax stock 007745) [87].

### Purification of primary B cells and *in vitro* stimulation

Spleen and peripheral lymph nodes were collected from 2~3 month old TG, TKO and wild type (WT) mice. WT and TG B cells were purified by depleting cells positive for AA4.1 (CD93), CD43 and CD5, while TKO B cells were purified by depleting cells positive for AA4.1 (CD93), CD43 and CD9 using MACS LD columns (Miltenyi Biotec) following manufacturer’s instructions. Purified B cells were cultured at a density of 5x10^6^ cells/ml for indicated amounts of time in B cell medium plus LPS (25μg/ml) and IL-4 (5ng/ml) in 37°C incubator, unless indicated otherwise. At the time of harvest, live cells were purified by Ficoll (GE Healthcare, 17-1440-02) to achieve a purity of >90% live cells and >98% B220^+^CD19^+^ B cells before further analysis. B cell medium was made of DMEM GlutaMAX (Gibco 10569) plus 10%v/v FCS, 1x non-essential amino acids (Corning, 25-025-CI), 10mM HEPES (Gibco, 15630), 50μM β-ME (Gibco, 21985), 1x Penn/Strep.

### Statistics

P values were determined by using two-tailed Student’s t-test. Statistical significance is displayed as *P < 0.05, **P < 0.01 and ***P < 0.001.

### Ribosome profiling, ERCC-RNA-seq, polysome profiling and microarray

Detailed procedures and analysis methods are present as a supplementary material. Please see “**S1 Methods”**

### Accession numbers

Microarray, RNA-Seq, and ribosome profiling data are available at NCBI Gene Expression Omnibus through the accession numbers GSE56379, GSE83734, and GSE83684.

## Supporting information

S1 Fig Genomic organization of the miR-17~92 family miRNAs in mice. Colors denote miRNA subfamilies. Members in each subfamily share the same seed region. Chr, chromosome.
(PDF)

S2 Fig Experimental approaches used in this study to investigate B cell transcriptome and translatome. (**A**) ERCC-RNA-seq to determine mRNA copy numbers. Pre-determined amounts of ERCC control RNA [52] and WT B cells were mixed together before RNA extraction and RNA-seq analysis. Normalized read counts (RPKM) of each mRNA species were compared to those of ERCC control RNA to calculate their copy numbers per cell. (**B**) Microarray to determine the impact of miR-17~92 on target transcriptome. Genes were analyzed only when they are significantly expressed (greater that 0.5 copy per cell) based on ERCC-RNA-seq results. (**C**) Schematic representation of ribosome profiling analysis of the translatome of activated B cells. (**D**) Schematic representation of polysome profiling analysis of activated B cells. The cytosolic compartment of activated B cells was separated into 20 fractions on a sucrose gradient (15%~45%), and the distribution of miRNAs and target mRNAs in these fractions was determined by qRT-PCR. Numbers in the graph indicate that number of ribosomes associated with mRNA. (**C-D**) B cells were activated for 25.5h. Fr, fraction number. CHX, cycloheximide.
(PDF)

S3 Fig Absolute quantification of mRNA abundance in B cells. (**A-B**) WT B cells were stimulated with LPS and IL-4 for indicated amounts of time (Naïve, 13.5h and 25.5h), spiked in with pre-determined amounts of ERCC control RNAs, and analyzed by RNA-seq. RPKM values of biological replicates were plotted against each other to show the high reproducibility of datasets (**A**). Each dot represents a unique gene. RPKM values of ERCC control RNAs were plotted against their copy numbers per cells (**B**). Blue lines indicate the linear regression, while gray areas represent the range of standard error. Note that the abundance of ERCC RNAs spans six orders of magnitude and is sufficient to cover the dynamic range of all endogenous mRNAs.
(PDF)

S4 Fig miR-17~92 expression levels and activities of major signaling pathways during B cell activation. (**A**) The induction and termination of the MAP kinase (indicated by pErk) and PI3K (indicated by pS6) pathways during B cell activation by 2μg/ml anti-IgM. (**B,C**) Northern blot analysis of miR-17~92 family miRNA expression in WT, TG, and TKO B cells. Purified B cells were stimulated with LPS and IL-4 for indicated amounts of time.
(PDF)

S5 Fig Microarray analysis of TKO, WT and TG B cells with target genes subsetted according to individual subfamily of miR-17~92. **(A-B)** PAR-CLIP identified miR-17~92 targets [40] were subsetted according to individual subfamily of miR-17~92. Results from different time points of activation of TG vs WT (A) and TKO vs WT (B) B cells were presented. Only significantly transcribed genes were analyzed. (**C-D**) Investigation of the top predicted target genes based on context++ scores from TargetScan 7.0 [55]. 128 top target genes were selected for each miRNA miR-17~92 subfamily, and the ones transcribed at greater than 0.5 copy per cell were analyzed. Numbers in parenthesis indicate the numbers of genes analyzed.
(PDF)

S6 Fig A summary of immunoblot analysis of miR-17~92 target genes in TG B cells. Among the 63 targets examined, quality immunoblots were obtained and quantified for 47 targets, while the other 16 were discarded due to poor antibody quality. Among the 47 targets quantified, only 13 showed reduced protein levels in TG B cells (**S7A Fig**), while the other 34 targets were either up-regulated or showed no change (**S7B and S7C Fig**). Notably, the majority of targets investigated has been previously validated as direct miR-17~92 targets in various cellular contexts (**S4 Table**). The 13 downregulated targets include negative regulators of the PI3K and NF-κB pathways, as well as five additional tumor suppressor genes. This is consistent with the previous observation that TG mice spontaneously developed B cell lymphoma with high penetrance [40].
(PDF)

S7 Fig Immunoblot analysis of 47 target gene protein levels in TG B cells. (**A**) The protein levels of 13 target genes showing reduced protein levels in TG B cells as determined by immunoblot. (**B,C**) The impact of transgenic miR-17~92 expression on the protein levels of the other 34 target genes. 28 targets showed little or time- and isoform dependent changes in their protein levels (**B**), while 6 targets were up-regulated in TG B cells (**c**). Note that *Bcl2l11* (Bim) and *E2f3* were suppressed in naïve but not activated B cells [40]. Two *Fbxw7* isoforms were detected and they were differentially regulated. Cell surface expression of *Tgfbr2* was quantified by FACS. Target gene protein levels were normalized to β-Actin, and their protein level in WT naïve B cells was arbitrarily set as 1.0. n.s., non-specific band.
(PDF)

S8 Fig Immunoblot analysis of 16 translation regulators that lack miR-17~92 binding sites. (**A**) Immunoblot analysis of 16 translation regulators in TG B cells. β-Actin was used as an internal control. (**B**) Quantification of protein and mRNA levels as measured by immunoblot and microarray, respectively.
(PDF)

S9 Fig Changes in ribosome footprint abundance highly correlates with changes in protein abundance. (**A**) Scatter plots evaluating the reproducibility of biological replicates of ribosome profiling. (**B,C**) Changes in protein expression of 47 miR-17~92 targets as determined by immunoblot (**S7 Fig**) were compared to changes in ribosome footprint abundance (**B**) and mRNA abundance (**C**). Note that changes in ribosome footprint abundance correlate with changes in protein abundance significantly better than with changes in mRNA abundance. Targets with significant protein reduction in TG B cells (13 targets from **S7A Fig**) were plotted as green dots, while other targets with no change or up-regulated in TG B cells (34 targets from **S7B and S7C Fig**) were plotted as open circles. The 6 targets whose protein levels were up-regulated in TG B cells (**S7C Fig**) were marked with gene names. (**D-E**) Changes in ribosome footprint abundance, protein and mRNA of the 13 targets suppressed in TG B cells (green dots in panel **B**) were further examined by ribosome profiling, immunoblot, and microarray. The relative contribution of translational repression and mRNA degradation to miR-17~92 regulation of these 13 target genes was approximately 4:1. *CD19* and *Actb* were used as positive and negative control, respectively. Linear regression (red lines) is constrained to intersect the negative control *Actb*. Slope of the linear regression is indicated in figures. TG mice are heterozygous for *CD19* and TG B cells express reduced levels of CD19 mRNA and protein [127].
(PDF)

S10 Fig Global analysis of the impact of miR-17~92 on target gene expression. (**A**) Transcribed and translated targets were determined by ERCC-RNA-seq and ribosome profiling analysis of 25.5h activated B cells. (**B**) Only a fraction of translated miR-17~92 targets were suppressed by transgenic miR-17~92 expression by 1.4 fold or more, as determined by changes in ribosome footprint abundance (termed ribo-downregulated TG targets). (**C**) The global impact of transgenic miR-17~92 expression on the mRNA levels and translation rates of translated targets. Dashed gray lines indicate median value of all translated targets, while red lines indicate median value of ribo-downregulated TG targets. (**D**) Only a fraction of translated miR-17~92 targets were suppressed by WT levels of miR-17~92 family miRNAs by 1.4 fold or more, as determined by changes in ribosome footprint abundance (termed ribo-upregulated TKO targets). (**E**) The global impact of miR-17~92 family miRNA deletion on mRNA levels and translation rates of translated targets. Dashed-gray lines indicate median value of all translated targets, while red lines indicate median value of ribo-upregulated TKO targets.
(PDF)

S11 Fig The impact of miR-17~92 family miRNA deletion on the target gene protein levels. (**A**) Immunoblot analysis of 13 targets suppressed in TG B cells (**S7A Fig**). (**B**) Quantification of the protein and mRNA levels of the 13 targets suppressed in TG B cells. (**C**) Immunoblot analysis of 10 targets de-repressed in TKO B cells. (**D**) Quantification of the protein and mRNA levels of the 10 targets de-repressed in TKO B cells. A summary is presented in **Fig 2E and 2F**. β-Actin was used as an internal control.
(PDF)

S12 Fig Direct regulation of group 1 target genes by miR-17~92 in wild type B cells. (**A**) Experimental scheme of reporter assays in primary B cells. (**B**) psiCheck-2 reporters with wild type 3’UTR or miR-17~92 binding site mutated 3’UTR were transfected into wild type B cells by electroporation and luciferase assay was performed as described in **Fig 7B**. Luciferase activity was normalized to psiCheck-2 reporters with wild type 3’UTR. When multiple miR-17~92 binding sites (BS) are present in a target gene 3’UTR and are far away from each other, multiple reporter constructs were generated, with each construct harboring one binding site. These reporter constructs were tested separately.
(PDF)

S13 Fig Polysome profiling directly captures changes in translational rate. A summary of currently available methods to assess the relative contribution of translational repression and mRNA degradation to miRNA regulation of target gene expression. The overall effect of miRNA on target gene expression can be divided into mRNA degradation (black arrow) and translational repression (red arrow). While the contribution of translational repression can be estimated by subtracting mRNA changes from protein changes, polysome profiling directly captures translational changes independent of mRNA changes.
(PDF)

S14 Fig Polysome profiles of WT and miRNA mutant B cells. Polysome profiles of activated B cells of indicated genotypes. Note that the overall A254 profiles of TKO, WT, TG, and miR-155 KO B cells were almost identical.
(PDF)

S15 Fig Absolute quantification of miR-155 and its binding sites, and polysome profiling analysis of miR-155 KO B cells. (**A**) Quantitative Northern blot to determine miR-155 copy number in 25.5h activated B cells. A summary of miR-155 copy number and the number of conserved miR-155 binding sites. Note that there are 7-fold more miR-155 binding sites than miR-155 molecules. The miR-155 binding sites were defined from previous PAR-CLIP analysis [128]. (**B**) Distribution of miR-155 and miR-21 in the sucrose gradient. Note that miR-21 was enriched in monosomes [32, 85], while miR-155 was enriched in light polysomes. (**C**) Distribution of previously validated miR-155 (AID, PU.1, Jarid2 and Peli1) and miR-17~92 target mRNAs in the sucrose gradient [88–91]. Deletion of miR-155 shifted miR-155 target mRNAs from fractions 10–11 to fractions 14–16, but had almost no significant effect on the distribution *Actb* and miR-17~92 target mRNAs.
(PDF)

S16 Fig Poly-RNA-seq analysis miR-17~92 targets in TG B cells and miR-155 targets in miR-155 KO B cells (**A**) Schematic representation of poly-RNA-seq analysis of activated B cells. Experimental approach is equivalent to that of polysome profiling, but collected specific fractions (Fr.10-11 and Fr. 14–16) for downstream RNA-seq analysis. (**B-C**) Consistent with polysome profiling results (**Fig 4 and S15 Fig**), miR-17~92 target mRNAs were enriched in fractions 10–11 in TG B cells, and miR-155 target mRNA were enriched in fractions 14–16 in miR-155 KO B cells.
(PDF)

S17 Fig Contribution of UTR length, number of binding sites, and seed type to target gene sensitivity to miRNA suppression. (**A**) The distribution of length of 5’UTR, CDS, and 3’UTR among miR-17~92 targets. (**B**) Location of miRNA binding sites in miR-17~92 targets. (**C**) Average number of conserved miR-17~92 binding sites in miR-17~92 targets. (**D**) The distribution of seed types among miR-17~92 binding sites. The conserved miR-17~92 binding sites were identified by PAR-CLIP analysis of human B cells [40].
(PDF)

S18 Fig Ribosome footprint distribution in translated miR-17~92 targets in TKO, WT, and TG B cells. Color lines depict relative ribosome occupancy in B cells of indicated genotypes. Grey shade represents the distribution of mapped reads from RNA-seq analysis of WT B cells. The first and last nucleotides of CDS are set as position 0 for 5’UTR and 3’UTR, respectively.
(PDF)

S19 Fig Molecular features of the *CD69* 5’UTR. The sequence of *CD69* 5’UTR and its molecular features. The locations of ribosome footprint, sub-optimal start codons, and the potential hairpin are indicated.
(PDF)

S20 Fig Molecular dissection of cis-elements of *CD69* 5’UTR in determining its sensitivity to suppression by miR-17~92. (**A**) A psiCheck-2-pd reporter with wt CD69 5’UTR and wt CD69 3’UTR was transfected into primary B cells expressing miR-17~92 at three different levels. Consistent with the endogenous CD69 gene (**Fig 2E**), the reporter gene was more sensitive to miR-17~92 depletion than to transgenic miR-17~92 expression. Results from both luciferase assay (protein) and qRT-PCR (mRNA) were shown. (**B**) Molecular dissection of cis-elements of CD69 5’UTR. Experiments were performed as described in **Fig 7B**. Luciferase activity was normalized to wt 3’UTR constructs. ΔHP, the left arm of the putative hairpin was deleted. Mut-uORF, two sub-optimal start codons were mutated.
(PDF)

S21 Fig The key target gene model. Key target genes emerge from a pool of hundreds of target genes via multiple mechanisms. There are mechanisms that regulate miRNA binding to target mRNAs, the consequences of miRNA binding, and cellular responses to reduced target gene protein levels. First, there are more binding sites than miRNA molecules and only a fraction of binding sites are occupied by miRNA-containing RISC complexes at any given time. Which binding sites are occupied by miRNA is determined by accessibility and affinity of binding sites to miRNA, as well as cellular concentrations of target mRNAs and miRNA. Second, miRNA binding does not necessarily warrant functional consequence. There are mechanisms that determine whether miRNA binding leads to changes in target gene protein levels and, if so, the amplitude of changes. 5’UTR is a part of the mechanisms regulating target gene sensitivity to miRNA suppression. Third, there are mechanisms that regulate cellular responses to changes in target gene protein levels. We speculate that reductions in the protein levels of many target genes brought about by a miRNA are functionally inconsequential, while a small number of target genes are sensitive to reduced protein levels in a given cellular context, as documented by the pathologies arising from haploinsufficiency. These target genes are therefore only a few percent of target genes with miRNA binding sites and serve as critical mediators of miRNA functions. They are the key target genes (**See** discussion). Numbers in figure indicate hypothetical target gene numbers in each category.
(PDF)

S22 Fig 5’UTR regulates target gene sensitivity to miRNA suppression. (**A**) Translation initiation occurs by a cap-dependent scanning mechanism, which requires binding of a trimeric complex eIF4F (consisting of 4E, 4G, 4A) to the m7G cap structure, followed by recruitment of the preinitiation complex (PIC) and scanning of PIC to the first AUG codon. The interaction between PABP and eIF4G circularizes the mRNA, and brings 3’UTR in close proximity to 5’UTR of the mRNA. This makes it possible for miRNA-containing RISCs associated with 3’UTR to directly regulate translation initiation at 5’UTR. (**B**) Our data suggest that miRNAs and secondary structures in 5’UTR cooperate to regulate translation initiation. For target mRNAs harboring secondary structures in 5’UTR, eIF4A or other RNA helicases are required to unwind these secondary structures, allowing PIC to efficiently scan through and to initiate translation. miRNA-containing RISCs may facilitate the dissociation of RNA helicases from 5’UTR, thereby stabilizing secondary structures and resulting in PIC accumulation in 5’UTR, repression of translation initiation, and a reduction in protein output [117, 118]. RISC, RNA-induced silencing complex. UTR, untranslated region. 4A, 4E, 4G, 4F, eukaryotic initiation factors (eIFs). PIC contains 40S ribosome subunit, Met-tRNAi, and eIFs 1, 1A, 2, 3, and 5.
(PDF)

S1 Table mRNA copy number per cell as determined by ERCC-RNA-seq. (XLSX)

S2 Table Microarray analysis of miR-17~92 target gene mRNA levels in TKO, WT, and TG B cells. (XLSX)

S3 Table Predicted miR-17~92 target genes with the highest context++ scores based on the most recent TargetScan 7.0 algorithm. (XLSX)

S4 Table miR-17~92 target genes investigated in this study. A list of miR-17~92 target genes investigated by immunoblot analysis. The experimentally identified miR-17~92 binding sites by human B cell PAR-CLIP [40], Burkitt’s lymphoma cell HITS-CLIP [79], HEK293 PAR-CLIP [35] and TargetScan 7.0 [55] were indicated. Gene names in parentheses are commonly known aliases. n.d., miR-17~92 family miRNA binding sites not detected in the CLIP dataset. References in which these genes were implicated as direct targets for miR-17~92 miRNAs were included [40, 45, 49, 56, 75–77, 80, 126, 129–197].
(XLSX)

S5 Table Ribosome footprint abundance in 25.5 h activated TKO, WT and TG B cells. (XLSX)

S6 Table Ribosome footprint density of ribo-downregulated TG targets and ribo-upregulated TKO targets in 25.5h activated TKO, WT and TG B cells. (XLSX)

S7 Table Absolute miRNA copy numbers in naïve and activated B cells as determined by quantitative Northern blot analysis. (XLSX)

S8 Table Target gene mRNA copy number and the number of conserved miR-17~92 binding sites on each target mRNA. (XLSX)

S1 Methods Supplemental experimental procedures including Northern blot, immunoblot, flow cytometry, qRT-PCR, cloning, reporter assay, ribosome profiling, ERCC-RNA-seq, polysome profiling, Microarray and bioinformatic analysis. (PDF)
