# Supplementary figures and images for: Differential Sensitivity of Target Genes to Translational Repression by miR-17~92
Source: PLoS Genet. 2017 Feb 27;13(2):e1006623. doi: 10.1371/journal.pgen.1006623 (PMC5348049; doi:10.1371/journal.pgen.1006623)

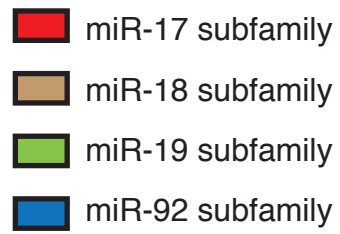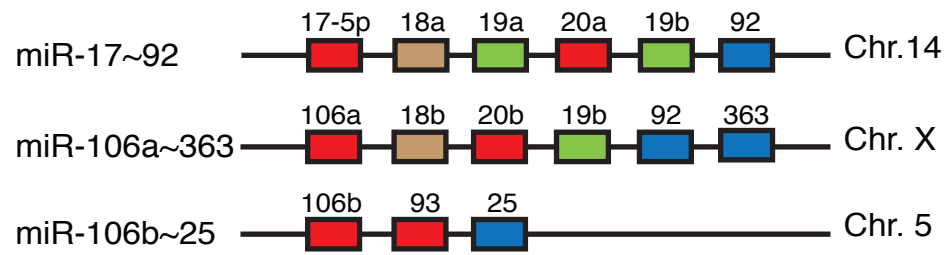

Supplement: S1 Fig — Colors denote miRNA subfamilies. Members in each subfamily share the same seed region. Chr, chromosome. (PDF) [file pgen.1006623.s001.pdf]

**A**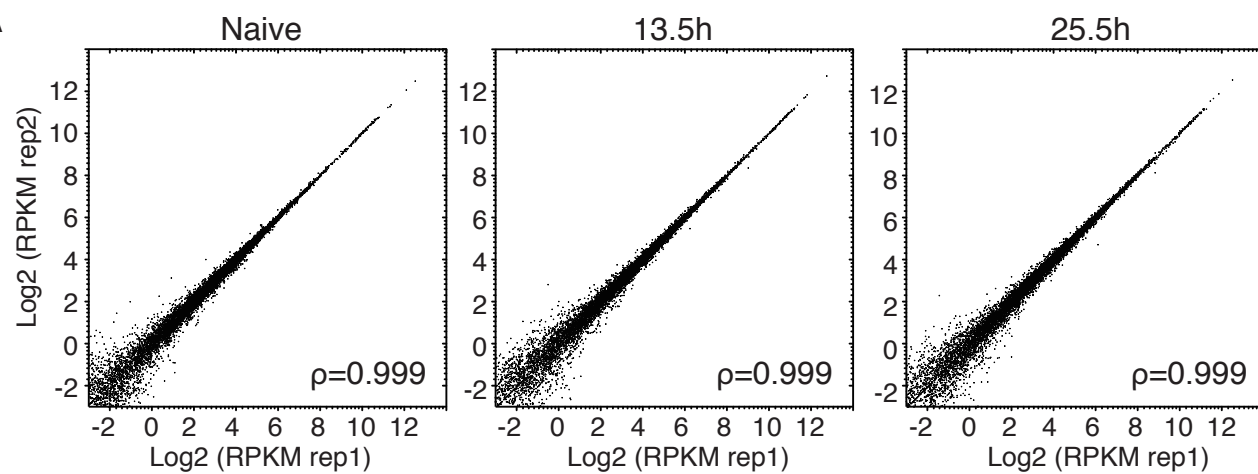**B**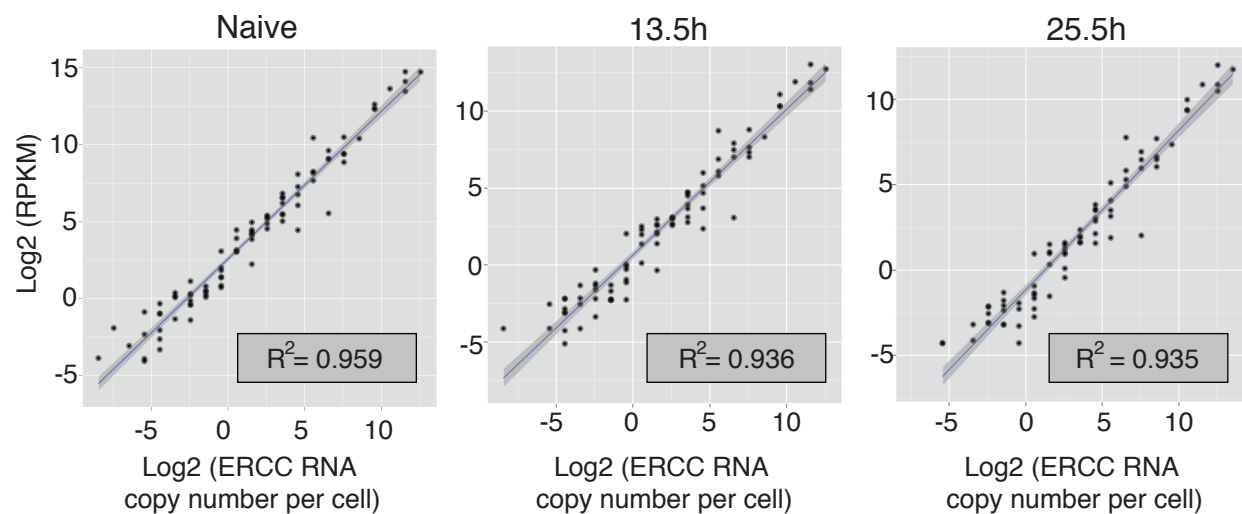

Supplement: S3 Fig — (A-B) WT B cells were stimulated with LPS and IL-4 for indicated amounts of time (Naïve, 13.5h and 25.5h), spiked in with pre-determined amounts of ERCC control RNAs, and analyzed by RNA-seq. RPKM values of biological replicates were plotted against each other to show the high reproducibility of datasets (A). Each dot represents a unique gene. RPKM values of ERCC control RNAs were plotted against their copy numbers per cells (B). Blue lines indicate the linear regression, while gray areas represent the range of standard error. Note that the abundance of ERCC RNAs spans six orders of magnitude and is sufficient to cover the dynamic range of all endogenous mRNAs. (PDF) [file pgen.1006623.s003.pdf]

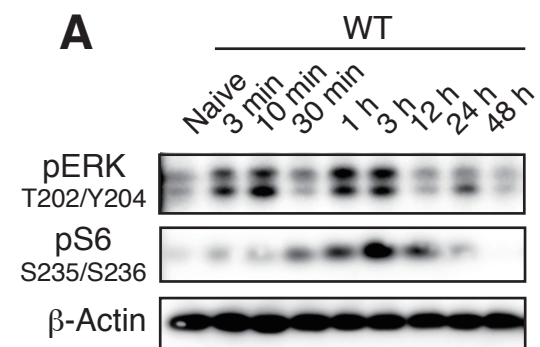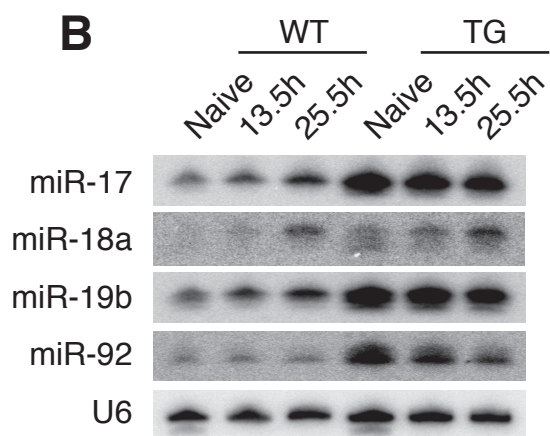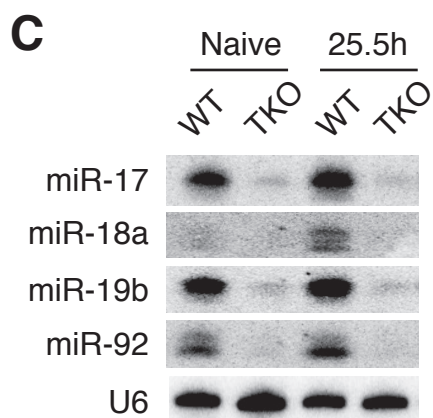

Supplement: S4 Fig — (A) The induction and termination of the MAP kinase (indicated by pErk) and PI3K (indicated by pS6) pathways during B cell activation by 2μg/ml anti-IgM. (B,C) Northern blot analysis of miR-17~92 family miRNA expression in WT, TG, and TKO B cells. Purified B cells were stimulated with LPS and IL-4 for indicated amounts of time. (PDF) [file pgen.1006623.s004.pdf]

**A**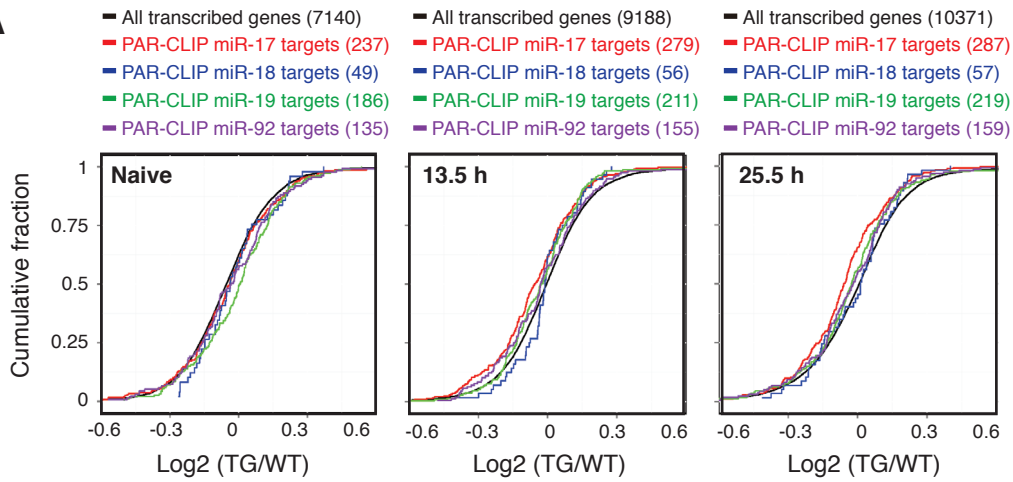**B**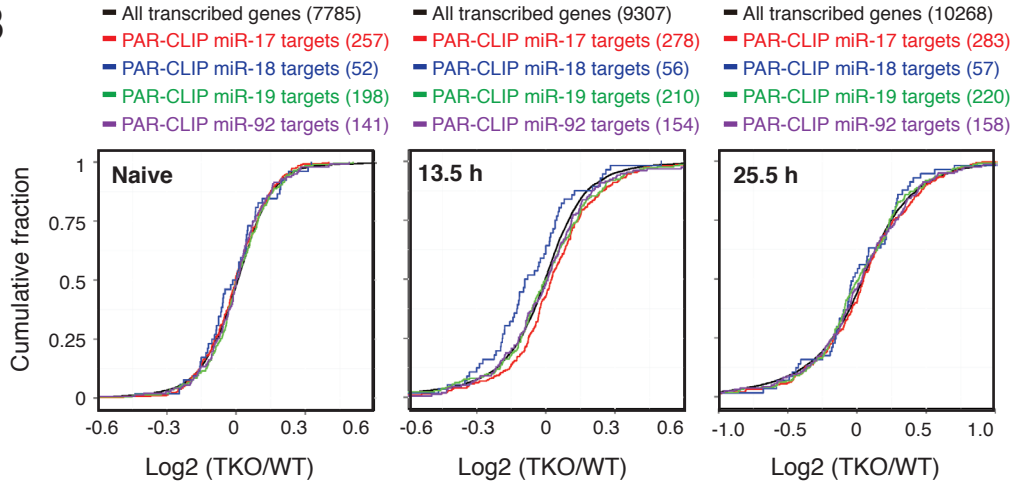**C**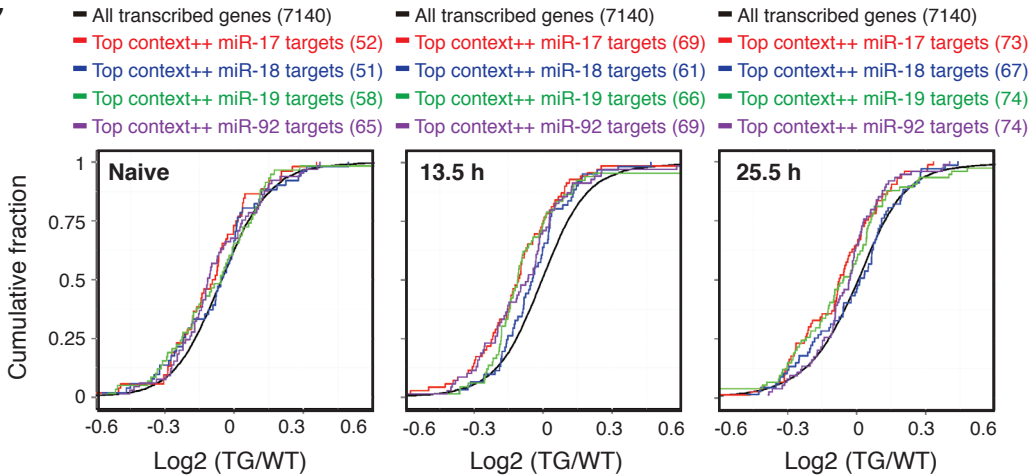**D**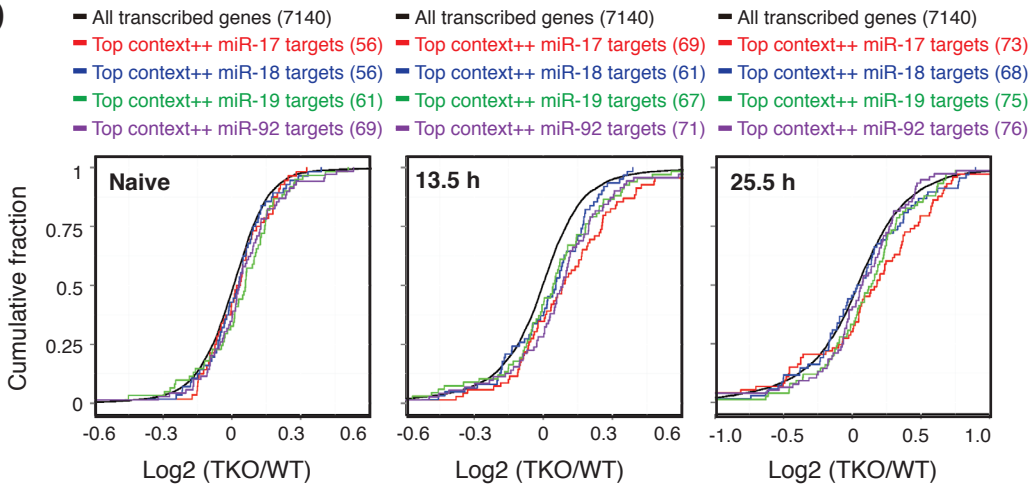

Supplement: S5 Fig — (A-B) PAR-CLIP identified miR-17~92 targets [40] were subsetted according to individual subfamily of miR-17~92. Results from different time points of activation of TG vs WT (A) and TKO vs WT (B) B cells were presented. Only significantly transcribed genes were analyzed. (C-D) Investigation of the top predicted target genes based on context++ scores from TargetScan 7.0 [55]. 128 top target genes were selected for each miRNA miR-17~92 subfamily, and the ones transcribed at greater than 0.5 copy per cell were analyzed. Numbers in parenthesis indicate the numbers of genes analyzed. (PDF) [file pgen.1006623.s005.pdf]

miR-17~92 targets examined by immunoblot in TG B cells (63)

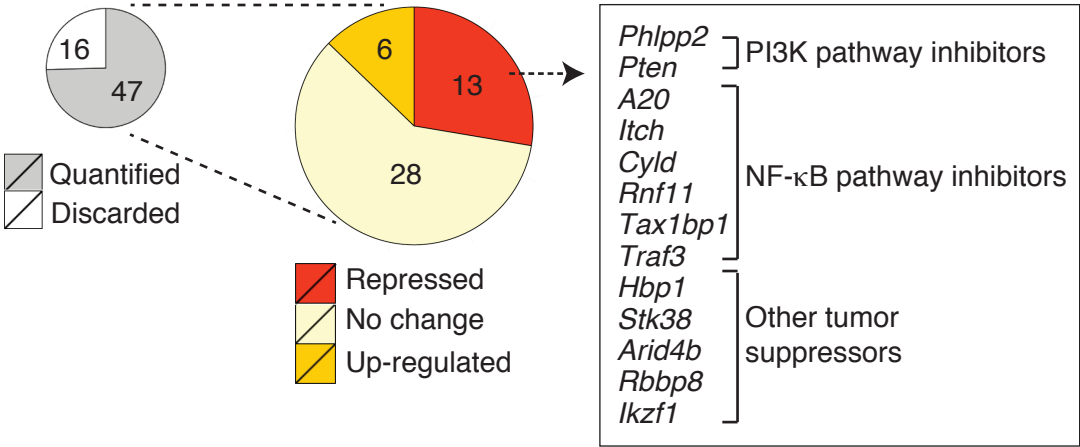

Supplement: S6 Fig — Among the 63 targets examined, quality immunoblots were obtained and quantified for 47 targets, while the other 16 were discarded due to poor antibody quality. Among the 47 targets quantified, only 13 showed reduced protein levels in TG B cells (S7A Fig), while the other 34 targets were either up-regulated or showed no change (S7B and S7C Fig). Notably, the majority of targets investigated has been previously validated as direct miR-17~92 targets in various cellular contexts (S4 Table). The 13 downregulated targets include negative regulators of the PI3K and NF-κB pathways, as well as five additional tumor suppressor genes. This is consistent with the previous observation that TG mice spontaneously developed B cell lymphoma with high penetrance [40]. (PDF) [file pgen.1006623.s006.pdf]

**A**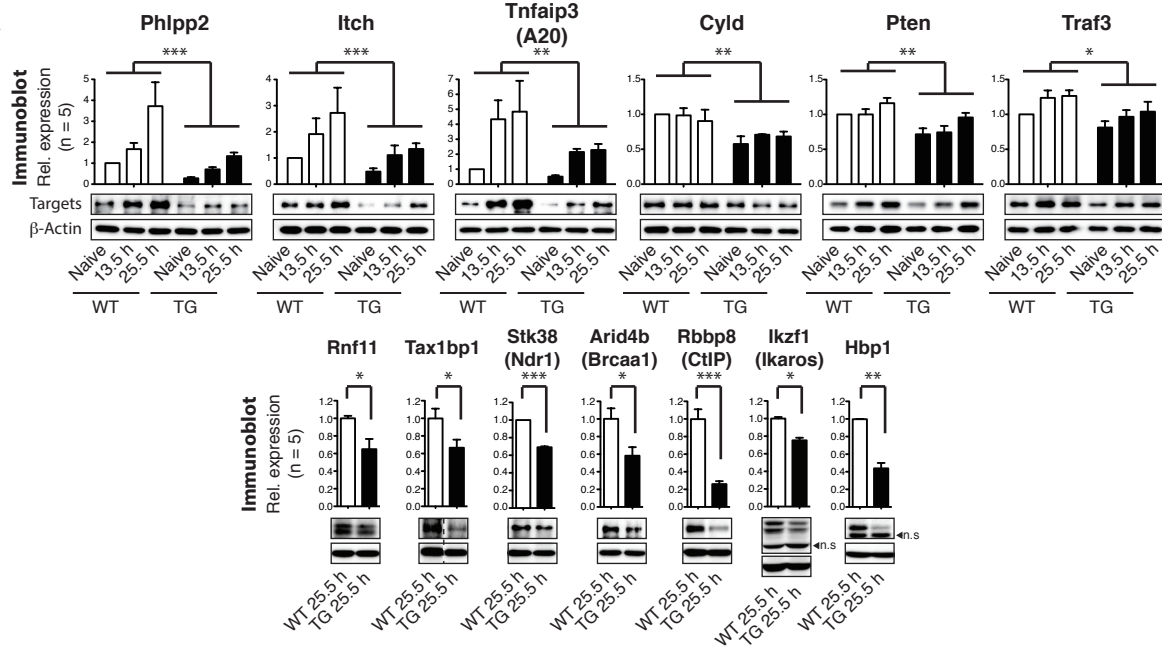**B**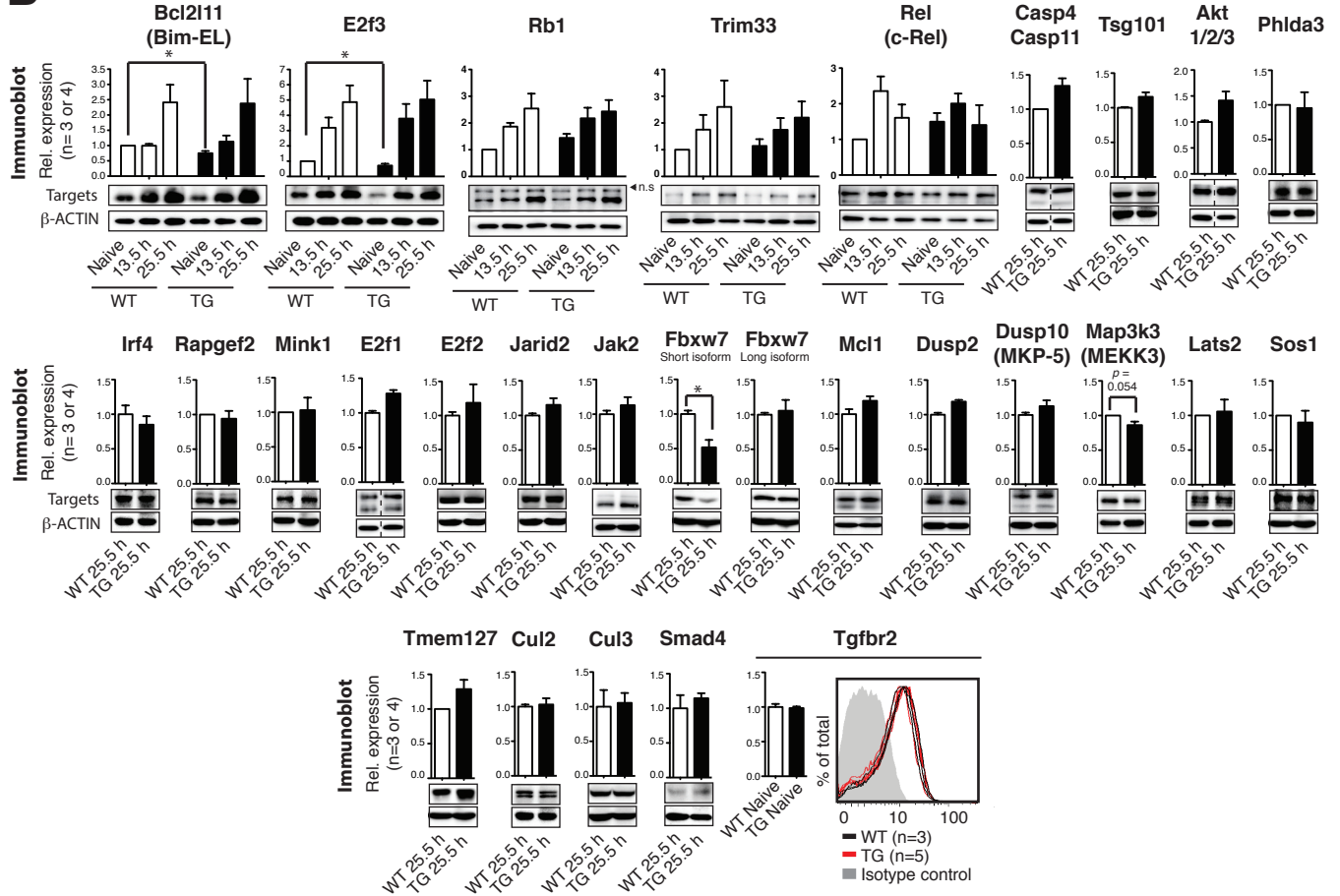**C**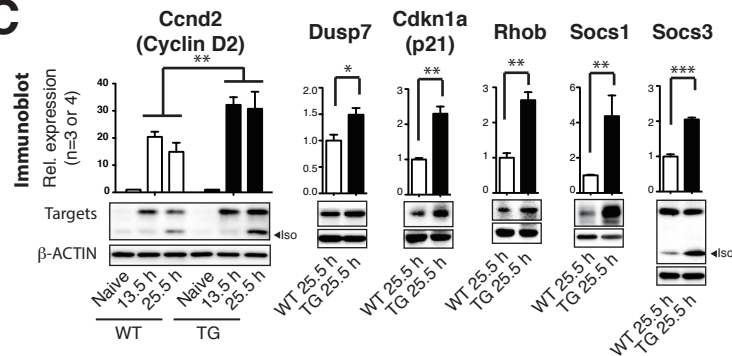

Supplement: S7 Fig — (A) The protein levels of 13 target genes showing reduced protein levels in TG B cells as determined by immunoblot. (B,C) The impact of transgenic miR-17~92 expression on the protein levels of the other 34 target genes. 28 targets showed little or time- and isoform dependent changes in their protein levels (B), while 6 targets were up-regulated in TG B cells (c). Note that Bcl2l11 (Bim) and E2f3 were suppressed in naïve but not activated B cells [40]. Two Fbxw7 isoforms were detected and they were differentially regulated. Cell surface expression of Tgfbr2 was quantified by FACS. Target gene protein levels were normalized to β-Actin, and their protein level in WT naïve B cells was arbitrarily set as 1.0. n.s., non-specific band. (PDF) [file pgen.1006623.s007.pdf]

**A**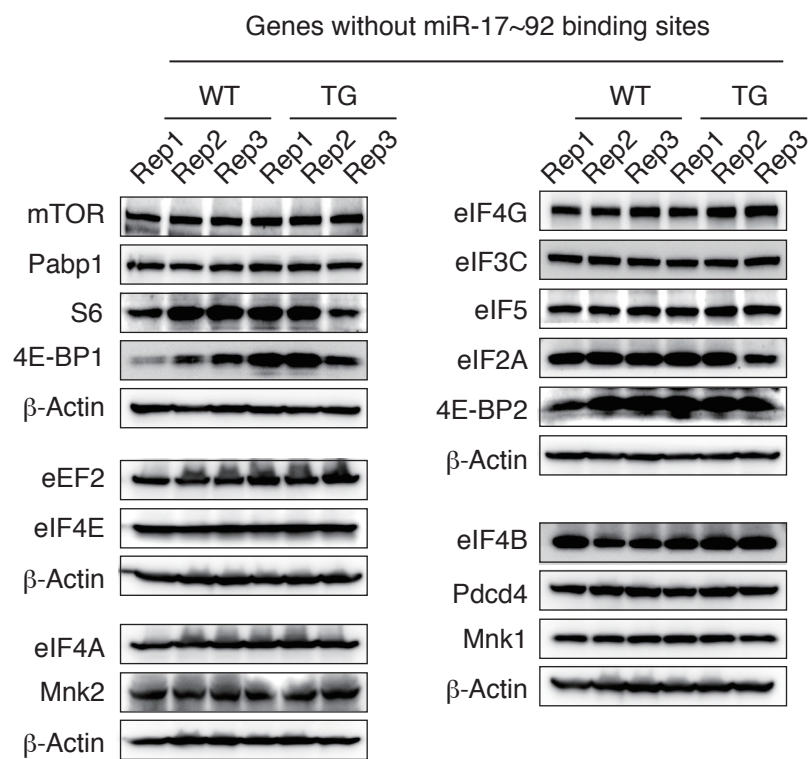**B**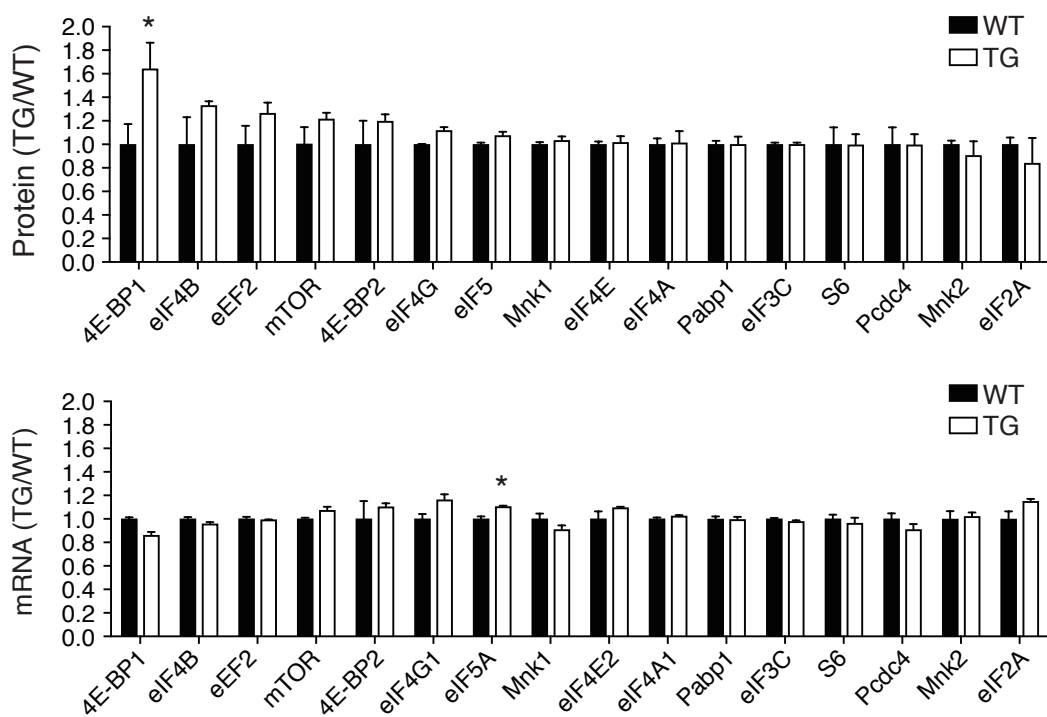

Supplement: S8 Fig — (A) Immunoblot analysis of 16 translation regulators in TG B cells. β-Actin was used as an internal control. (B) Quantification of protein and mRNA levels as measured by immunoblot and microarray, respectively. (PDF) [file pgen.1006623.s008.pdf]

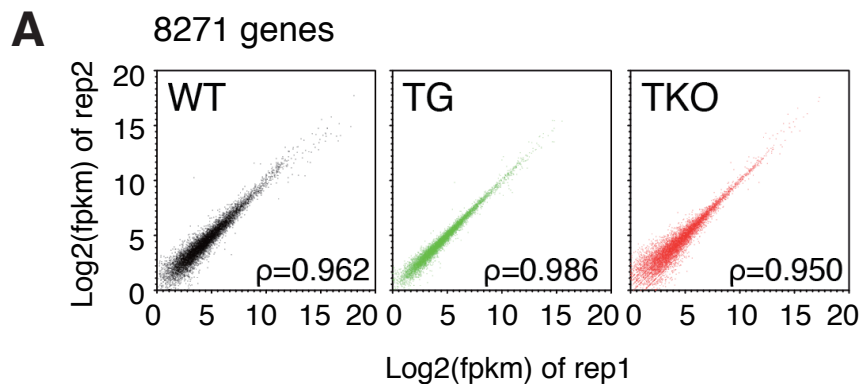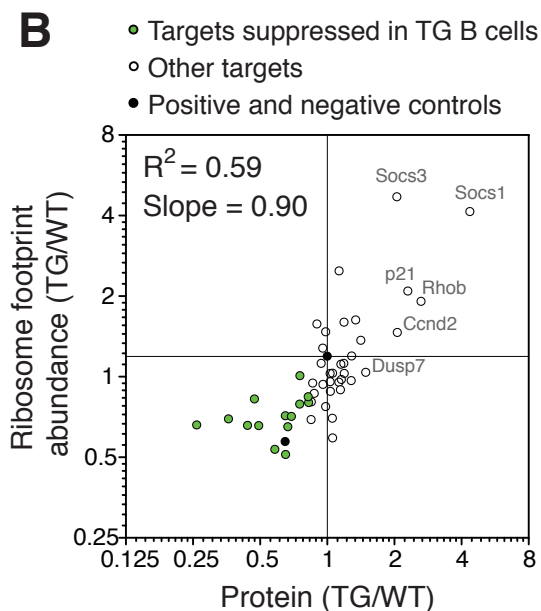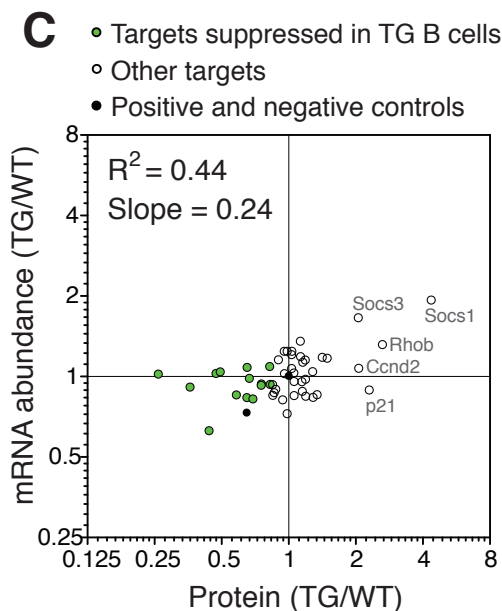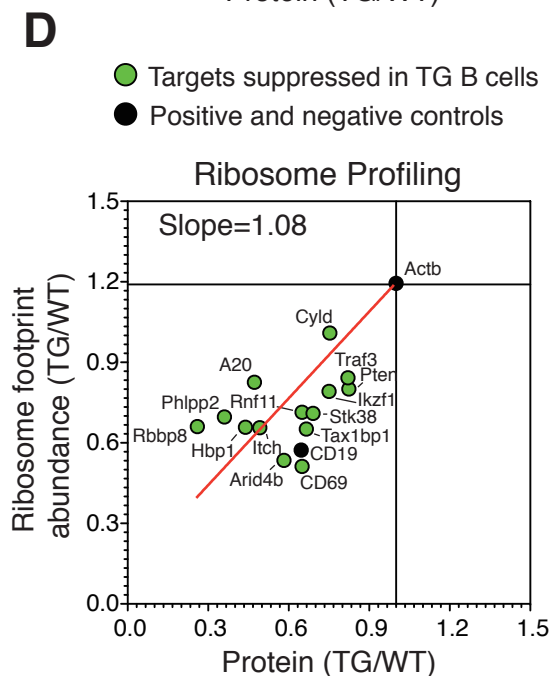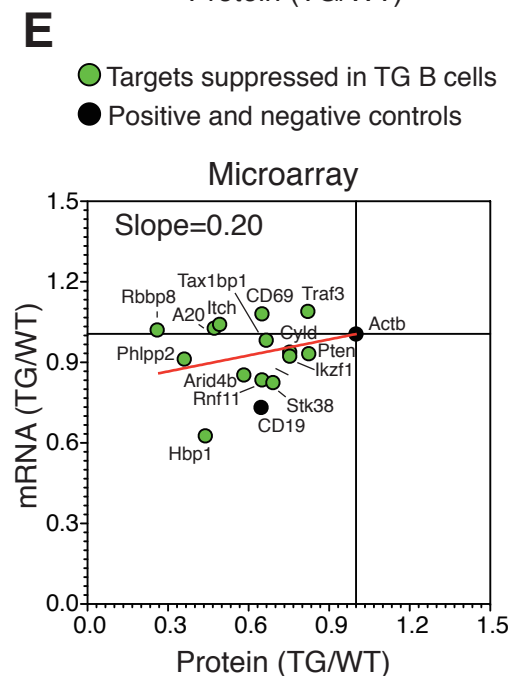

Supplement: S9 Fig — (A) Scatter plots evaluating the reproducibility of biological replicates of ribosome profiling. (B,C) Changes in protein expression of 47 miR-17~92 targets as determined by immunoblot (S7 Fig) were compared to changes in ribosome footprint abundance (B) and mRNA abundance (C). Note that changes in ribosome footprint abundance correlate with changes in protein abundance significantly better than with changes in mRNA abundance. Targets with significant protein reduction in TG B cells (13 targets from S7A Fig) were plotted as green dots, while other targets with no change or up-regulated in TG B cells (34 targets from S7B and S7C Fig) were plotted as open circles. The 6 targets whose protein levels were up-regulated in TG B cells (S7C Fig) were marked with gene names. (D-E) Changes in ribosome footprint abundance, protein and mRNA of the 13 targets suppressed in TG B cells (green dots in panel B) were further examined by ribosome profiling, immunoblot, and microarray. The relative contribution of translational repression and mRNA degradation to miR-17~92 regulation of these 13 target genes was approximately 4:1. CD19 and Actb were used as positive and negative control, respectively. Linear regression (red lines) is constrained to intersect the negative control Actb. Slope of the linear regression is indicated in figures. TG mice are heterozygous for CD19 and TG B cells express reduced levels of CD19 mRNA and protein [127]. (PDF) [file pgen.1006623.s009.pdf]

**A**

Targets suppressed in TG B cells

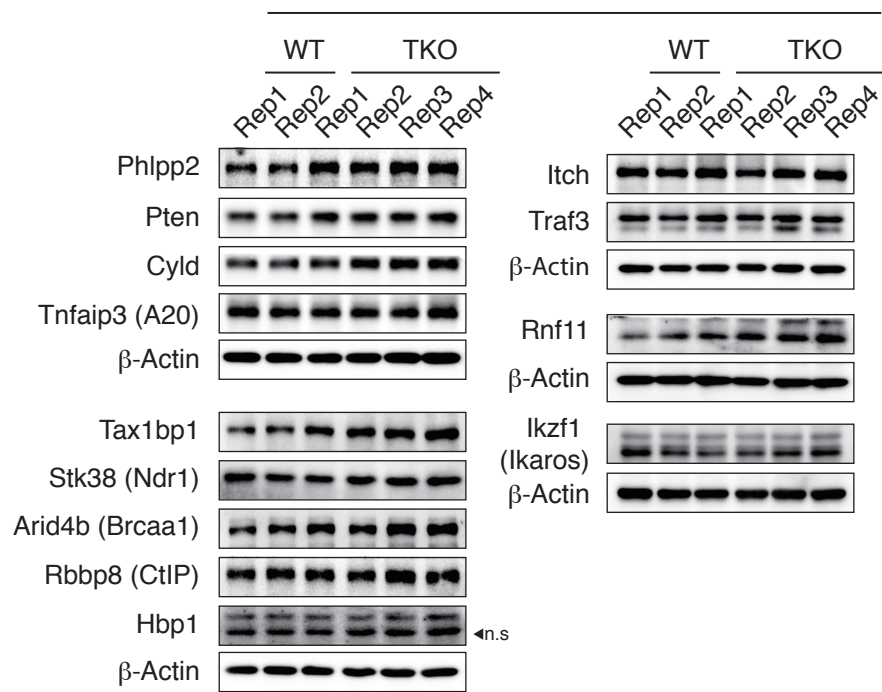**B**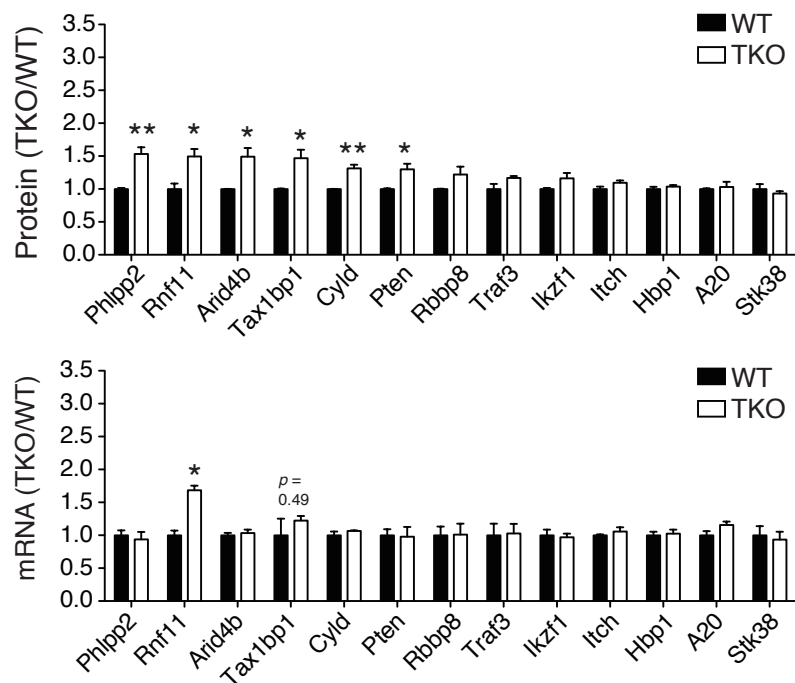**C**

Targets upregulated in TKO B cells

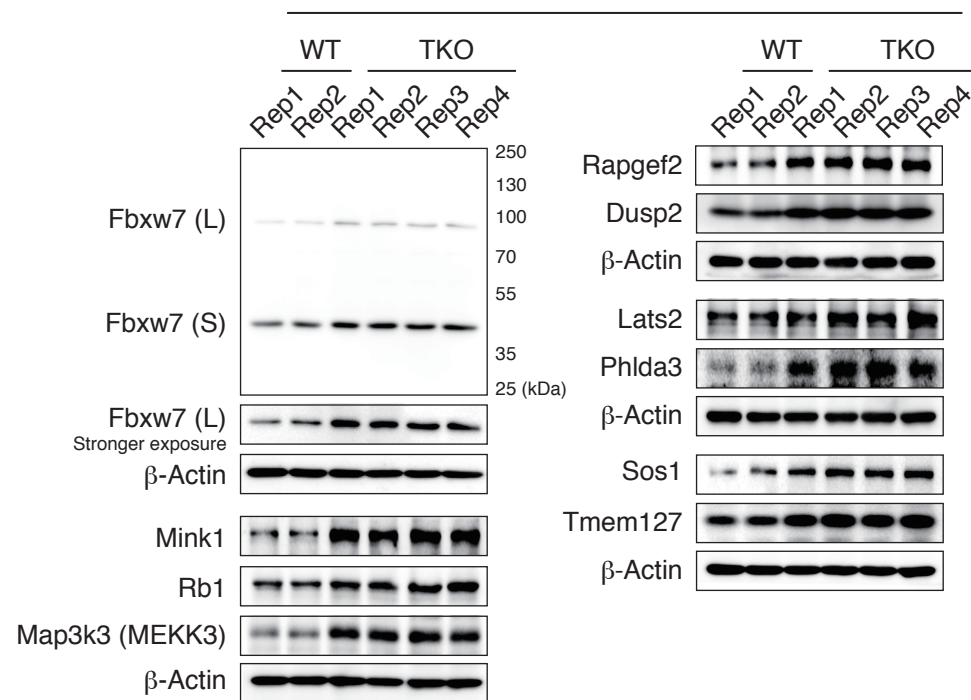**D**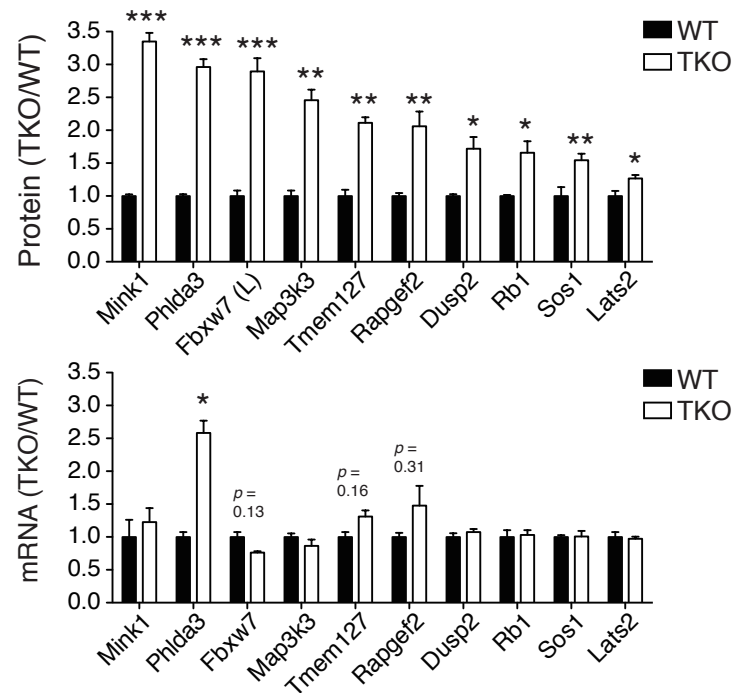

Supplement: S11 Fig — (A) Immunoblot analysis of 13 targets suppressed in TG B cells (S7A Fig). (B) Quantification of the protein and mRNA levels of the 13 targets suppressed in TG B cells. (C) Immunoblot analysis of 10 targets de-repressed in TKO B cells. (D) Quantification of the protein and mRNA levels of the 10 targets de-repressed in TKO B cells. A summary is presented in Fig 2E and 2F. β-Actin was used as an internal control. (PDF) [file pgen.1006623.s011.pdf]

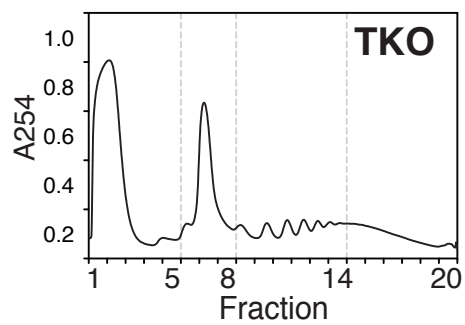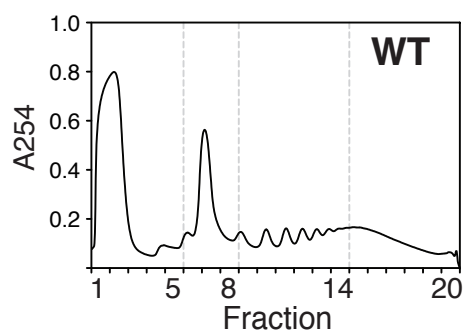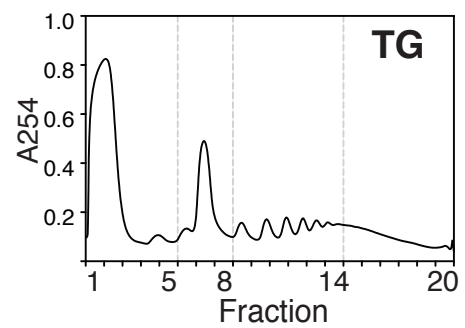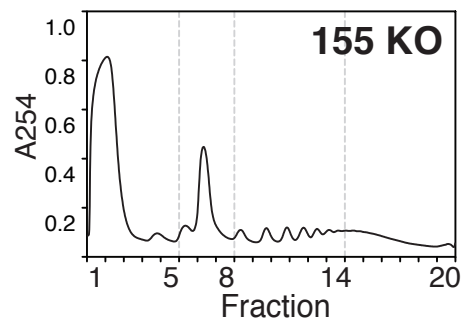

Supplement: S14 Fig — Polysome profiles of activated B cells of indicated genotypes. Note that the overall A254 profiles of TKO, WT, TG, and miR-155 KO B cells were almost identical. (PDF) [file pgen.1006623.s014.pdf]

**A**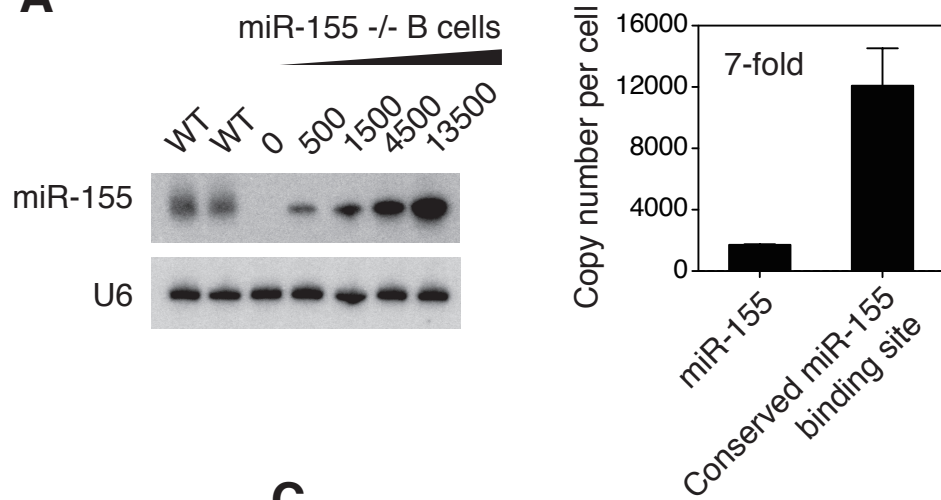**B**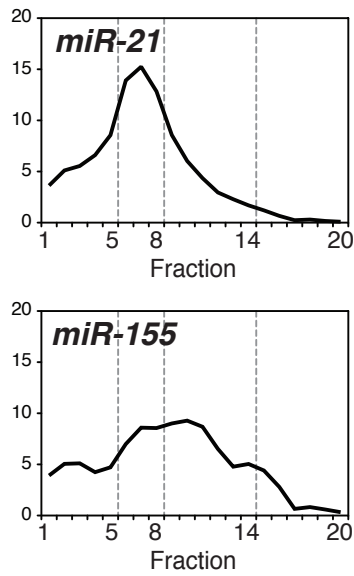**C**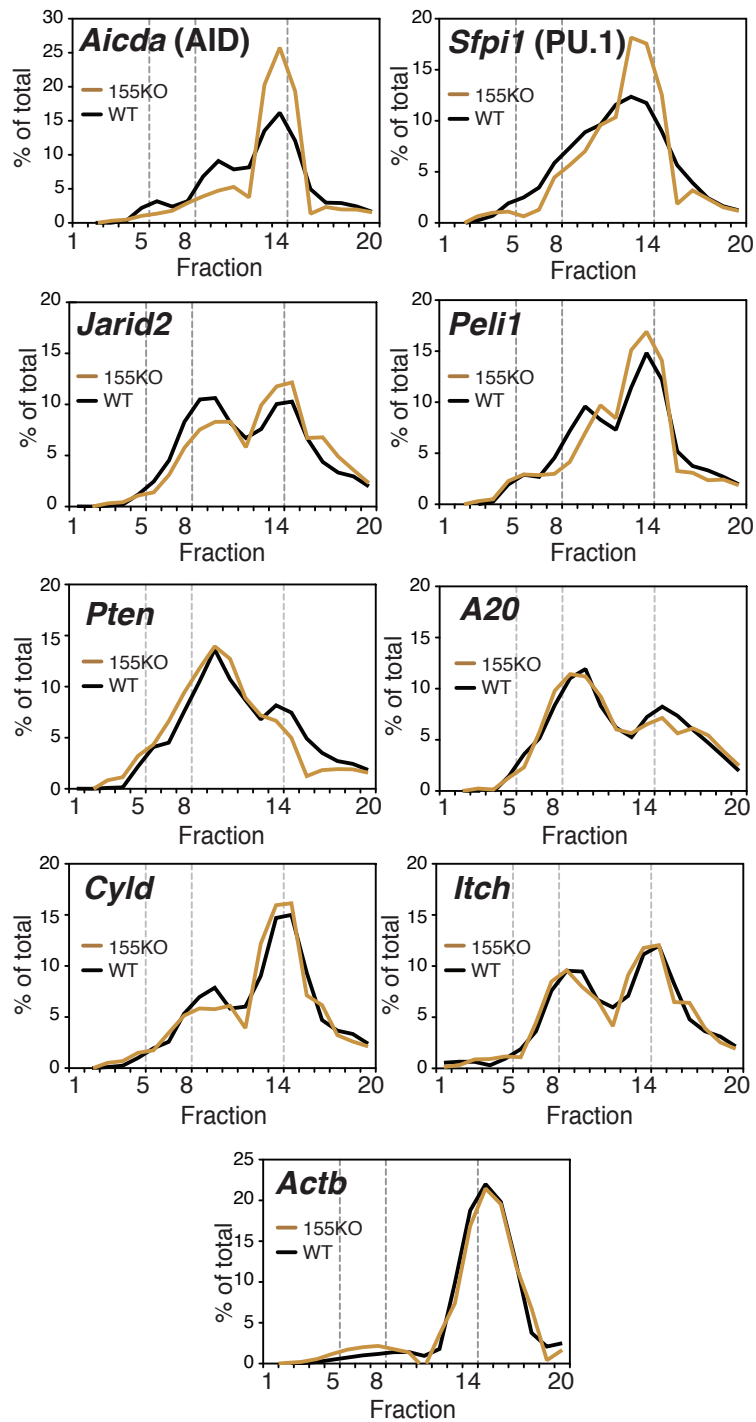

miR-155 targets

miR-17~92 targets

Control

Supplement: S15 Fig — (A) Quantitative Northern blot to determine miR-155 copy number in 25.5h activated B cells. A summary of miR-155 copy number and the number of conserved miR-155 binding sites. Note that there are 7-fold more miR-155 binding sites than miR-155 molecules. The miR-155 binding sites were defined from previous PAR-CLIP analysis [128]. (B) Distribution of miR-155 and miR-21 in the sucrose gradient. Note that miR-21 was enriched in monosomes [32, 85], while miR-155 was enriched in light polysomes. (C) Distribution of previously validated miR-155 (AID, PU.1, Jarid2 and Peli1) and miR-17~92 target mRNAs in the sucrose gradient [88–91]. Deletion of miR-155 shifted miR-155 target mRNAs from fractions 10–11 to fractions 14–16, but had almost no significant effect on the distribution Actb and miR-17~92 target mRNAs. (PDF) [file pgen.1006623.s015.pdf]

**A**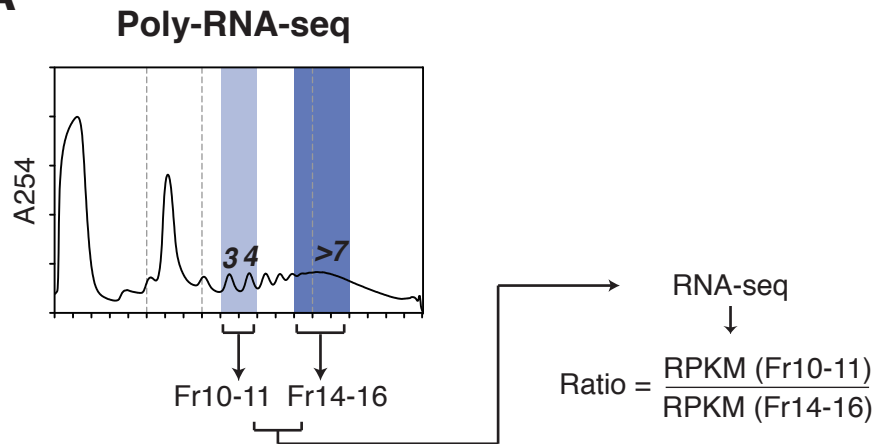**B**

miR-17~92 targets in TG B cells

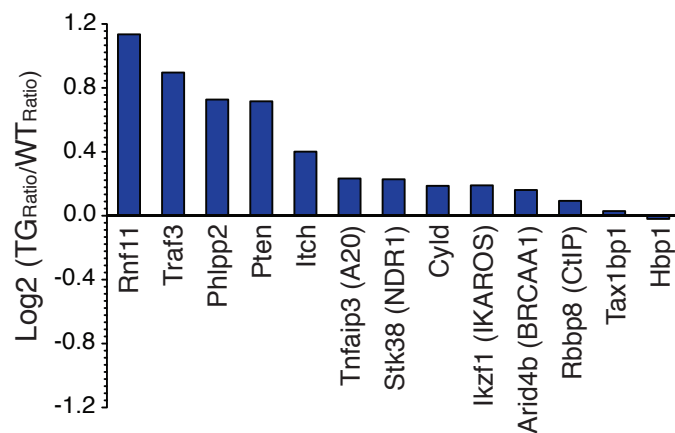**C**

miR-155 targets in miR-155 KO B cells

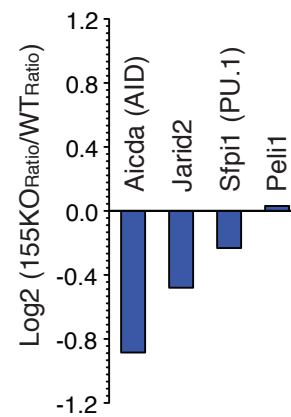

Supplement: S16 Fig — (A) Schematic representation of poly-RNA-seq analysis of activated B cells. Experimental approach is equivalent to that of polysome profiling, but collected specific fractions (Fr.10-11 and Fr. 14–16) for downstream RNA-seq analysis. (B-C) Consistent with polysome profiling results (Fig 4 and S15 Fig), miR-17~92 target mRNAs were enriched in fractions 10–11 in TG B cells, and miR-155 target mRNA were enriched in fractions 14–16 in miR-155 KO B cells. (PDF) [file pgen.1006623.s016.pdf]

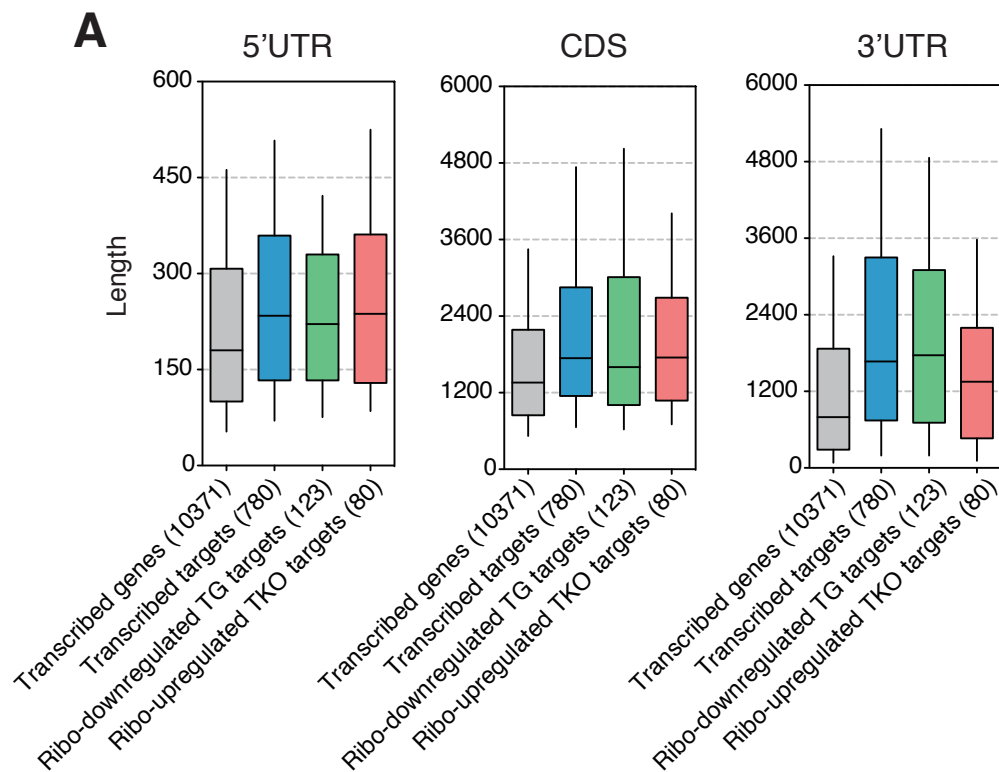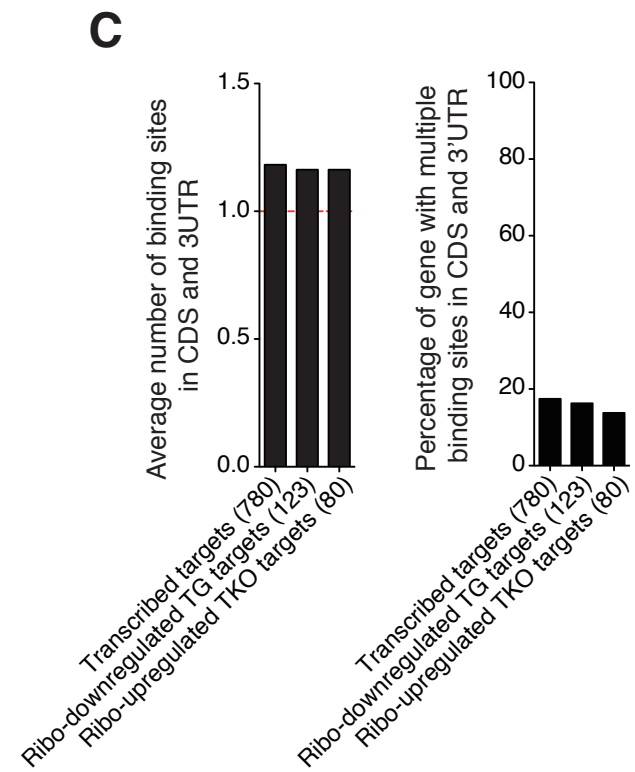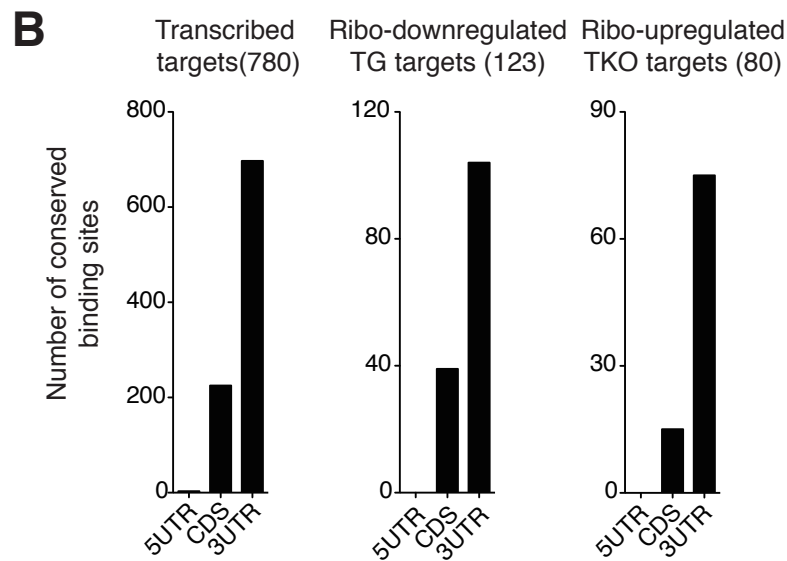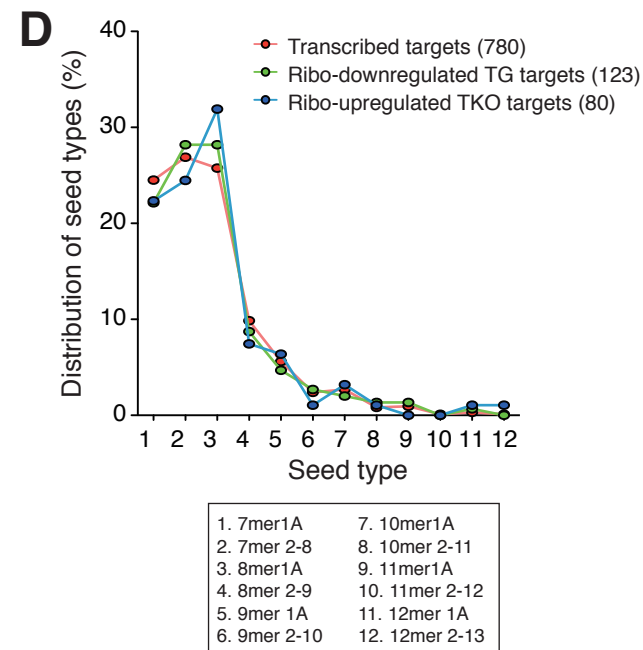

Supplement: S17 Fig — (A) The distribution of length of 5’UTR, CDS, and 3’UTR among miR-17~92 targets. (B) Location of miRNA binding sites in miR-17~92 targets. (C) Average number of conserved miR-17~92 binding sites in miR-17~92 targets. (D) The distribution of seed types among miR-17~92 binding sites. The conserved miR-17~92 binding sites were identified by PAR-CLIP analysis of human B cells [40]. (PDF) [file pgen.1006623.s017.pdf]

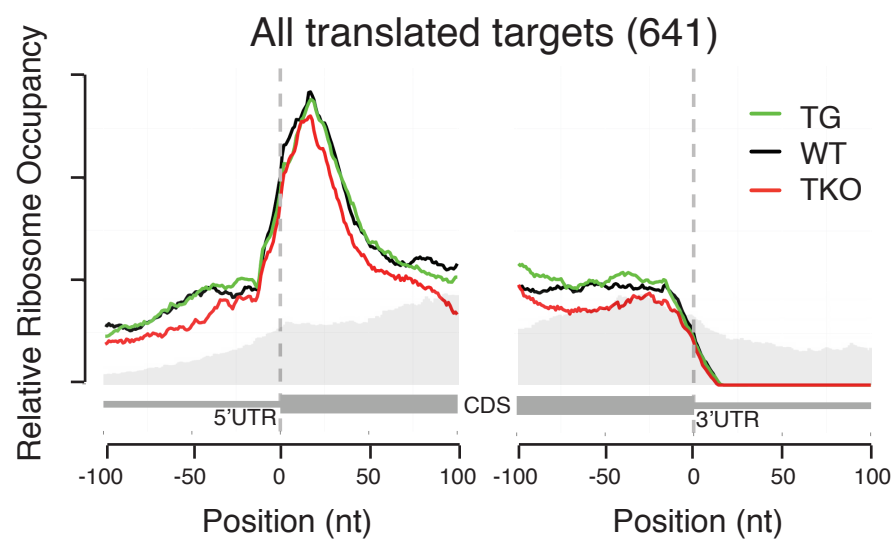

Supplement: S18 Fig — Color lines depict relative ribosome occupancy in B cells of indicated genotypes. Grey shade represents the distribution of mapped reads from RNA-seq analysis of WT B cells. The first and last nucleotides of CDS are set as position 0 for 5’UTR and 3’UTR, respectively. (PDF) [file pgen.1006623.s018.pdf]

*CD69* 5'UTR (84 nt)

**Sub-optimal  
start codons**

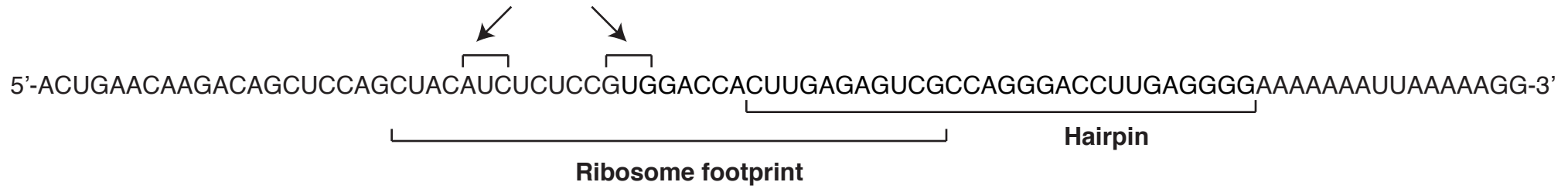

Supplement: S19 Fig — The sequence of CD69 5’UTR and its molecular features. The locations of ribosome footprint, sub-optimal start codons, and the potential hairpin are indicated. (PDF) [file pgen.1006623.s019.pdf]

**A**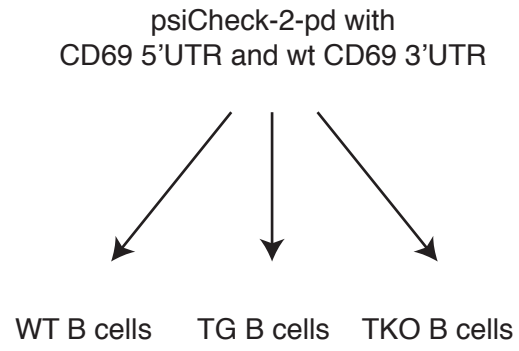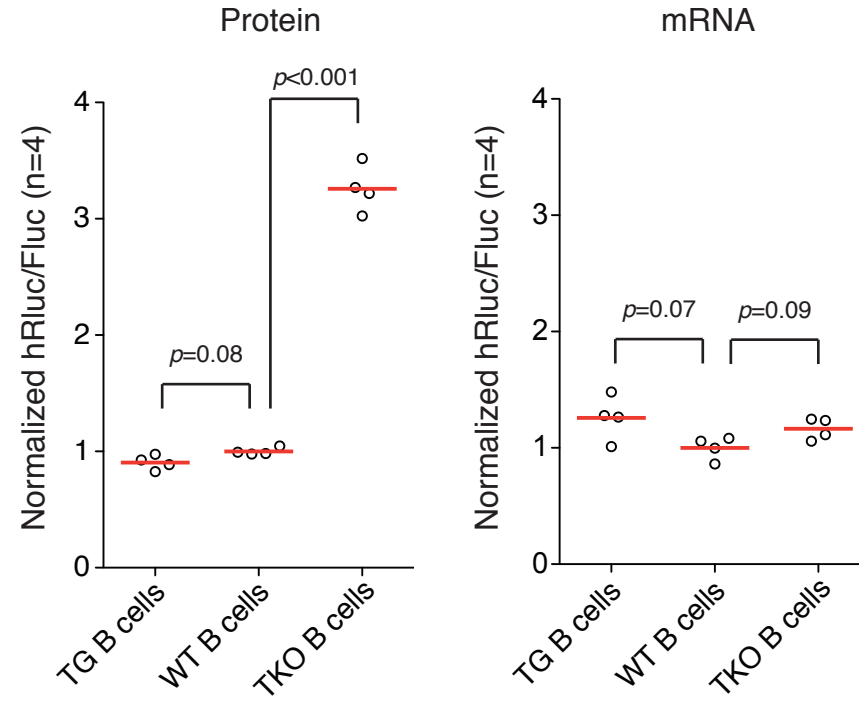**B**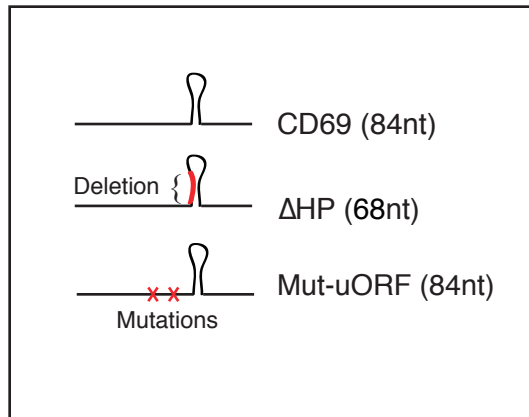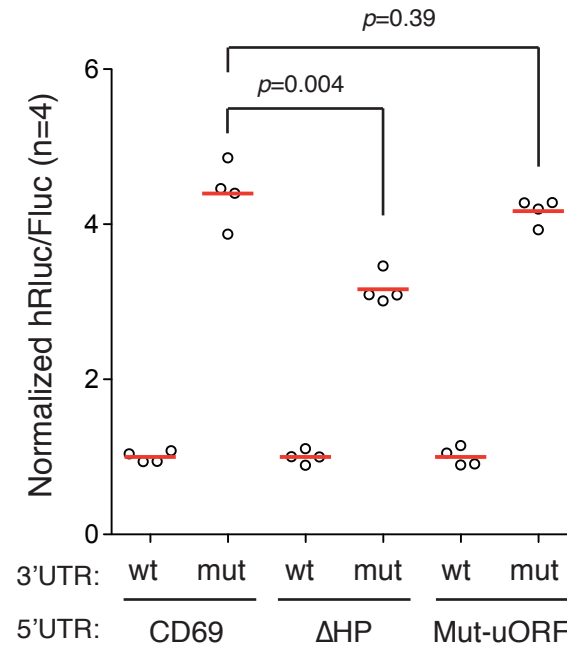

Supplement: S20 Fig — (A) A psiCheck-2-pd reporter with wt CD69 5’UTR and wt CD69 3’UTR was transfected into primary B cells expressing miR-17~92 at three different levels. Consistent with the endogenous CD69 gene (Fig 2E), the reporter gene was more sensitive to miR-17~92 depletion than to transgenic miR-17~92 expression. Results from both luciferase assay (protein) and qRT-PCR (mRNA) were shown. (B) Molecular dissection of cis-elements of CD69 5’UTR. Experiments were performed as described in Fig 7B. Luciferase activity was normalized to wt 3’UTR constructs. ΔHP, the left arm of the putative hairpin was deleted. Mut-uORF, two sub-optimal start codons were mutated. (PDF) [file pgen.1006623.s020.pdf]

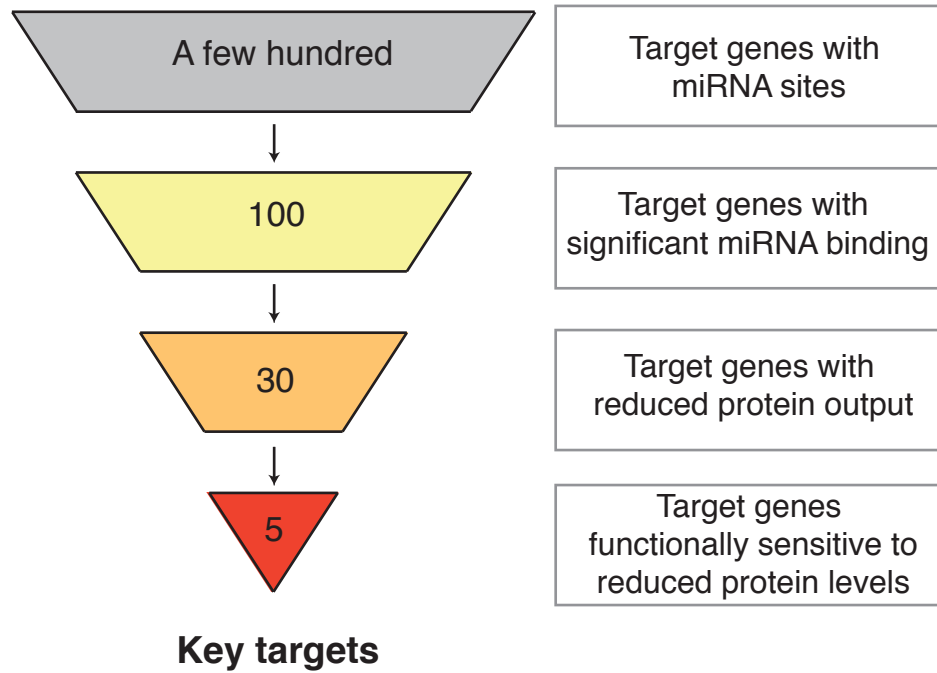

Supplement: S21 Fig — Key target genes emerge from a pool of hundreds of target genes via multiple mechanisms. There are mechanisms that regulate miRNA binding to target mRNAs, the consequences of miRNA binding, and cellular responses to reduced target gene protein levels. First, there are more binding sites than miRNA molecules and only a fraction of binding sites are occupied by miRNA-containing RISC complexes at any given time. Which binding sites are occupied by miRNA is determined by accessibility and affinity of binding sites to miRNA, as well as cellular concentrations of target mRNAs and miRNA. Second, miRNA binding does not necessarily warrant functional consequence. There are mechanisms that determine whether miRNA binding leads to changes in target gene protein levels and, if so, the amplitude of changes. 5’UTR is a part of the mechanisms regulating target gene sensitivity to miRNA suppression. Third, there are mechanisms that regulate cellular responses to changes in target gene protein levels. We speculate that reductions in the protein levels of many target genes brought about by a miRNA are functionally inconsequential, while a small number of target genes are sensitive to reduced protein levels in a given cellular context, as documented by the pathologies arising from haploinsufficiency. These target genes are therefore only a few percent of target genes with miRNA binding sites and serve as critical mediators of miRNA functions. They are the key target genes (See discussion). Numbers in figure indicate hypothetical target gene numbers in each category. (PDF) [file pgen.1006623.s021.pdf]

**A**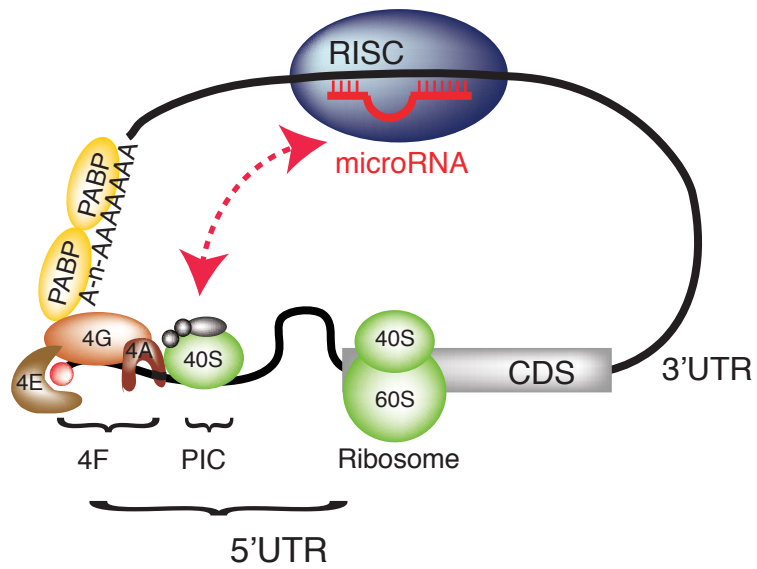**B**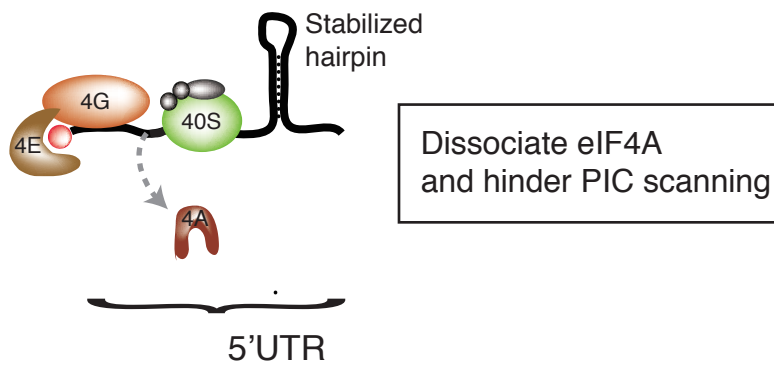

Supplement: S22 Fig — (A) Translation initiation occurs by a cap-dependent scanning mechanism, which requires binding of a trimeric complex eIF4F (consisting of 4E, 4G, 4A) to the m7G cap structure, followed by recruitment of the preinitiation complex (PIC) and scanning of PIC to the first AUG codon. The interaction between PABP and eIF4G circularizes the mRNA, and brings 3’UTR in close proximity to 5’UTR of the mRNA. This makes it possible for miRNA-containing RISCs associated with 3’UTR to directly regulate translation initiation at 5’UTR. (B) Our data suggest that miRNAs and secondary structures in 5’UTR cooperate to regulate translation initiation. For target mRNAs harboring secondary structures in 5’UTR, eIF4A or other RNA helicases are required to unwind these secondary structures, allowing PIC to efficiently scan through and to initiate translation. miRNA-containing RISCs may facilitate the dissociation of RNA helicases from 5’UTR, thereby stabilizing secondary structures and resulting in PIC accumulation in 5’UTR, repression of translation initiation, and a reduction in protein output [117, 118]. RISC, RNA-induced silencing complex. UTR, untranslated region. 4A, 4E, 4G, 4F, eukaryotic initiation factors (eIFs). PIC contains 40S ribosome subunit, Met-tRNAi, and eIFs 1, 1A, 2, 3, and 5. (PDF) [file pgen.1006623.s022.pdf]
