# Supplementary material for: Differential Sensitivity of Target Genes to Translational Repression by miR-17~92
Source: PLoS Genet. 2017 Feb 27;13(2):e1006623. doi: 10.1371/journal.pgen.1006623 (PMC5348049; doi:10.1371/journal.pgen.1006623)
Supplement: S2 Fig — (A) ERCC-RNA-seq to determine mRNA copy numbers. Pre-determined amounts of ERCC control RNA [52] and WT B cells were mixed together before RNA extraction and RNA-seq analysis. Normalized read counts (RPKM) of each mRNA species were compared to those of ERCC control RNA to calculate their copy numbers per cell. (B) Microarray to determine the impact of miR-17~92 on target transcriptome. Genes were analyzed only when they are significantly expressed (greater that 0.5 copy per cell) based on ERCC-RNA-seq results. (C) Schematic representation of ribosome profiling analysis of the translatome of activated B cells. (D) Schematic representation of polysome profiling analysis of activated B cells. The cytosolic compartment of activated B cells was separated into 20 fractions on a sucrose gradient (15%~45%), and the distribution of miRNAs and target mRNAs in these fractions was determined by qRT-PCR. Numbers in the graph indicate that number of ribosomes associated with mRNA. (C-D) B cells were activated for 25.5h. Fr, fraction number. CHX, cycloheximide. (PDF) [file pgen.1006623.s002.pdf]

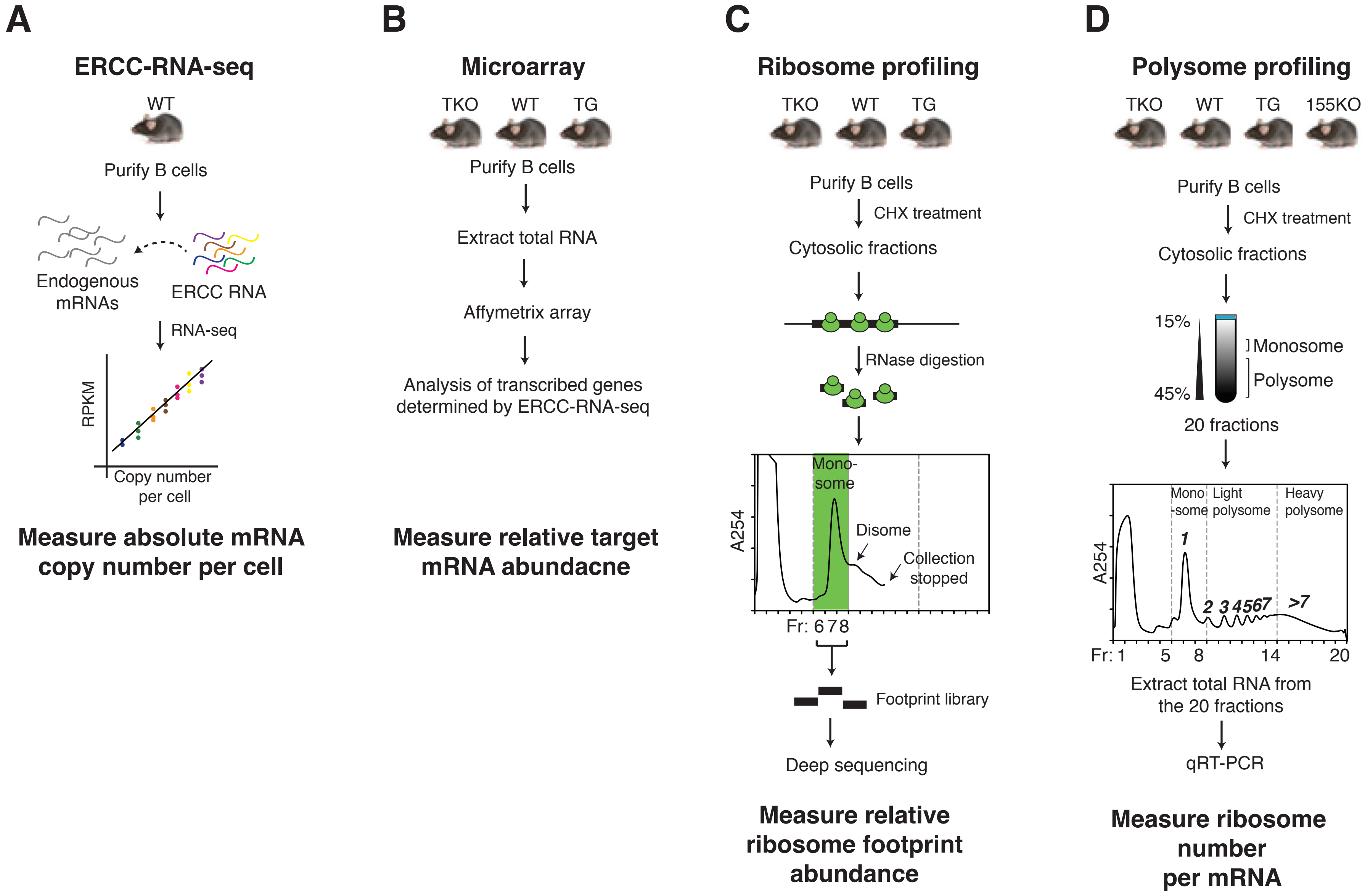
A
ERCC-RNA-seq
WT
Purify B cells
Endogenous mRNAs
ERCC RNA
RNA-seq
RPKM
Copy number per cell
Measure absolute mRNA copy number per cell
B
Microarray
TKO
WT
TG
Purify B cells
Extract total RNA
Affymetrix array
Analysis of transcribed genes determined by ERCC-RNA-seq
Measure relative target mRNA abundacne
C
Ribosome profiling
TKO
WT
TG
Purify B cells
CHX treatment
Cytosolic fractions
RNase digestion
A254
Mono-some
Disome
Collection stopped
Fr: 6 7 8
Footprint library
Deep sequencing
Measure relative ribosome footprint abundance
D
Polysome profiling
TKO
WT
TG
155KO
Purify B cells
CHX treatment
Cytosolic fractions
15%
45%
Monosome
Polysome
20 fractions
A254
Mono -some
Light polysome
Heavy polysome
1
2 3 4 5 6 7
>7
Fr: 1 5 8 14 20
Extract total RNA from the 20 fractions
qRT-PCR
Measure ribosome number per mRNA
