# Supplementary material for: Differential Sensitivity of Target Genes to Translational Repression by miR-17~92
Source: PLoS Genet. 2017 Feb 27;13(2):e1006623. doi: 10.1371/journal.pgen.1006623 (PMC5348049; doi:10.1371/journal.pgen.1006623)
Supplement: S10 Fig — (A) Transcribed and translated targets were determined by ERCC-RNA-seq and ribosome profiling analysis of 25.5h activated B cells. (B) Only a fraction of translated miR-17~92 targets were suppressed by transgenic miR-17~92 expression by 1.4 fold or more, as determined by changes in ribosome footprint abundance (termed ribo-downregulated TG targets). (C) The global impact of transgenic miR-17~92 expression on the mRNA levels and translation rates of translated targets. Dashed gray lines indicate median value of all translated targets, while red lines indicate median value of ribo-downregulated TG targets. (D) Only a fraction of translated miR-17~92 targets were suppressed by WT levels of miR-17~92 family miRNAs by 1.4 fold or more, as determined by changes in ribosome footprint abundance (termed ribo-upregulated TKO targets). (E) The global impact of miR-17~92 family miRNA deletion on mRNA levels and translation rates of translated targets. Dashed-gray lines indicate median value of all translated targets, while red lines indicate median value of ribo-upregulated TKO targets. (PDF) [file pgen.1006623.s010.pdf]

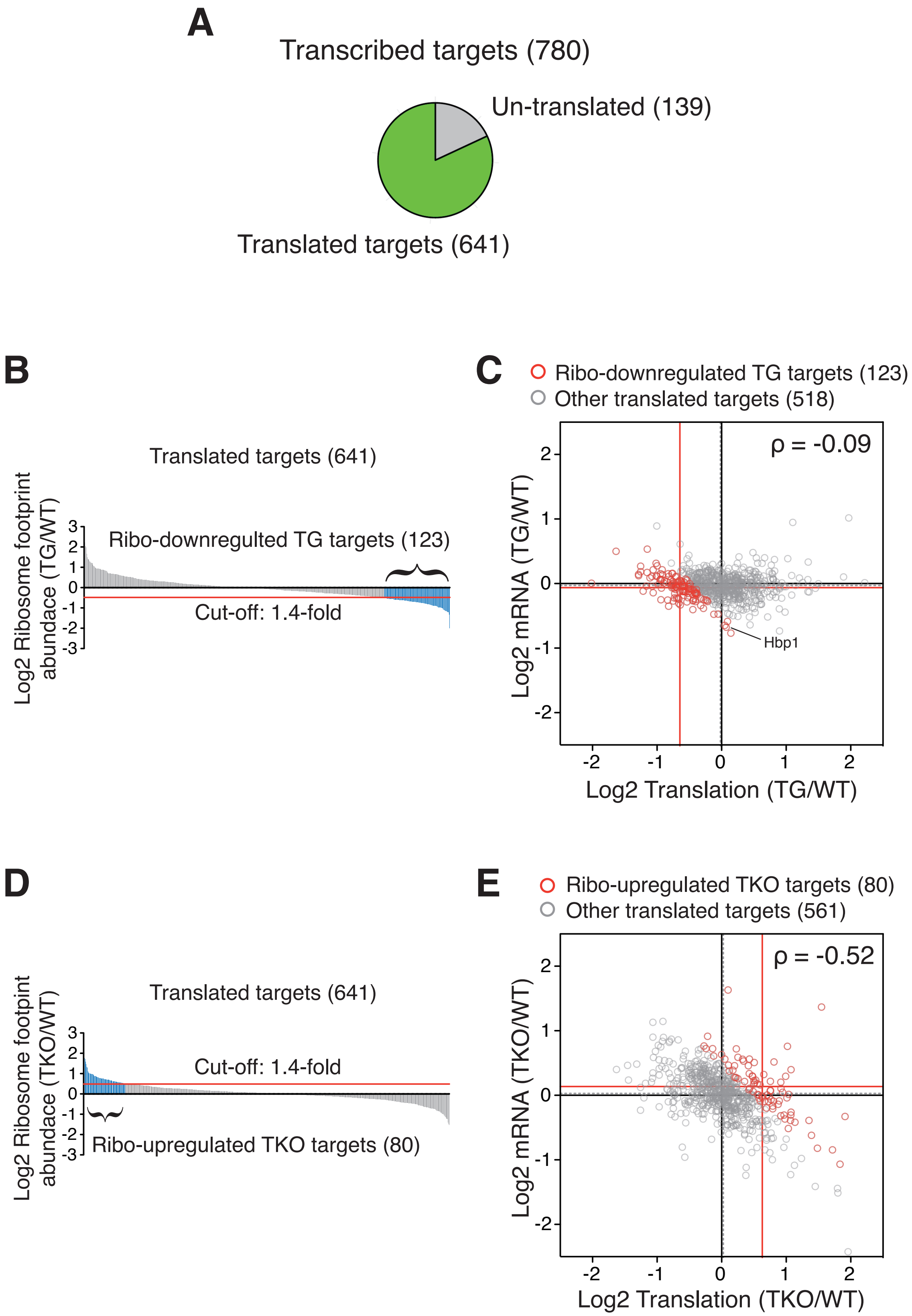
A
Transcribed targets (780)
Un-translated (139)
Translated targets (641)
B
Translated targets (641)
Ribo-downregulted TG targets (123)
Cut-off: 1.4-fold
Log2 Ribosome footprint abundace (TG/WT)
C
Ribo-downregulated TG targets (123)
Other translated targets (518)
ρ = -0.09
Hbp1
Log2 mRNA (TG/WT)
Log2 Translation (TG/WT)
D
Translated targets (641)
Cut-off: 1.4-fold
Ribo-upregulated TKO targets (80)
Log2 Ribosome footprint abundace (TKO/WT)
E
Ribo-upregulated TKO targets (80)
Other translated targets (561)
ρ = -0.52
Log2 mRNA (TKO/WT)
Log2 Translation (TKO/WT)
