# Supplementary material for: Differential Sensitivity of Target Genes to Translational Repression by miR-17~92
Source: PLoS Genet. 2017 Feb 27;13(2):e1006623. doi: 10.1371/journal.pgen.1006623 (PMC5348049; doi:10.1371/journal.pgen.1006623)
Supplement: S12 Fig — (A) Experimental scheme of reporter assays in primary B cells. (B) psiCheck-2 reporters with wild type 3’UTR or miR-17~92 binding site mutated 3’UTR were transfected into wild type B cells by electroporation and luciferase assay was performed as described in Fig 7B. Luciferase activity was normalized to psiCheck-2 reporters with wild type 3’UTR. When multiple miR-17~92 binding sites (BS) are present in a target gene 3’UTR and are far away from each other, multiple reporter constructs were generated, with each construct harboring one binding site. These reporter constructs were tested separately. (PDF) [file pgen.1006623.s012.pdf]

A

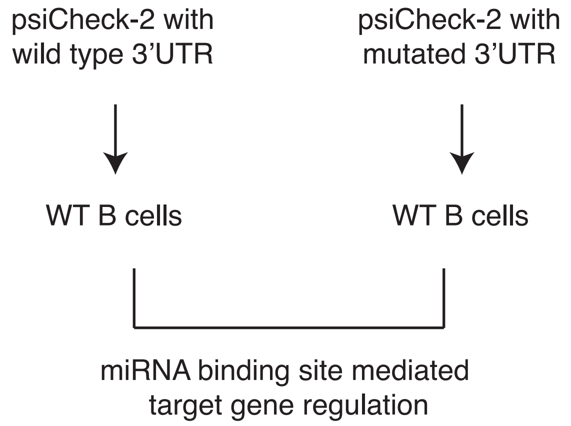
psiCheck-2 with
wild type 3'UTR
psiCheck-2 with
mutated 3'UTR
WT B cells
WT B cells
miRNA binding site mediated
target gene regulation

B

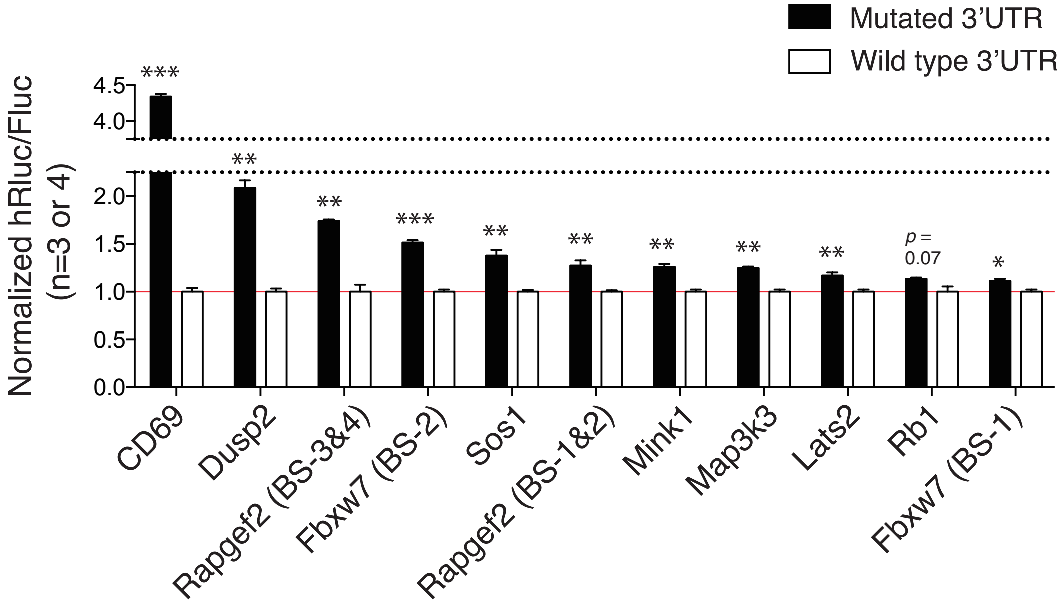
Mutated 3'UTR
Wild type 3'UTR
Normalized hRluc/Fluc
(n=3 or 4)
4.5
4.0
2.0
1.5
1.0
0.5
0.0
***
**
**
***
**
**
**
**
**
p =
0.07
*
CD69
Dusp2
Rapgef2 (BS-3&4)
Fbxw7 (BS-2)
Sos1
Rapgef2 (BS-1&2)
Mink1
Map3k3
Lats2
Rb1
Fbxw7 (BS-1)
