# Supplementary material for: Differential Sensitivity of Target Genes to Translational Repression by miR-17~92
Source: PLoS Genet. 2017 Feb 27;13(2):e1006623. doi: 10.1371/journal.pgen.1006623 (PMC5348049; doi:10.1371/journal.pgen.1006623)
Supplement: S13 Fig — A summary of currently available methods to assess the relative contribution of translational repression and mRNA degradation to miRNA regulation of target gene expression. The overall effect of miRNA on target gene expression can be divided into mRNA degradation (black arrow) and translational repression (red arrow). While the contribution of translational repression can be estimated by subtracting mRNA changes from protein changes, polysome profiling directly captures translational changes independent of mRNA changes. (PDF) [file pgen.1006623.s013.pdf]

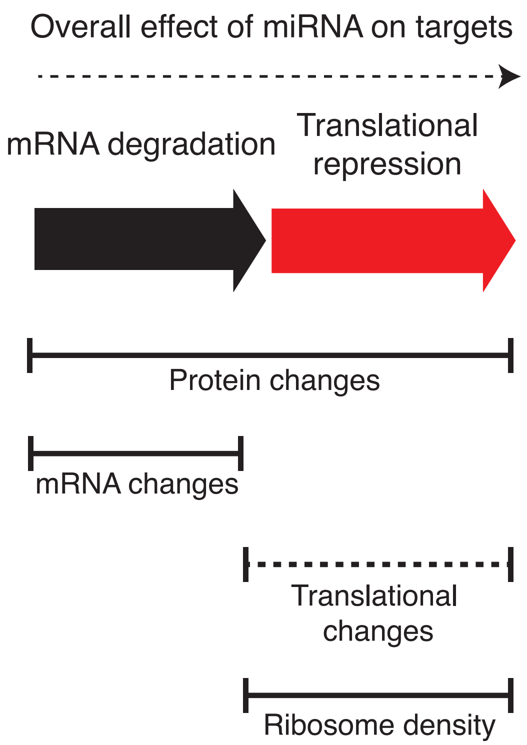

Method	Direct vs. Indirect
Immunoblot<br>Proteomics<br>Ribosome Profiling	Direct
qRT-PCR<br>Microarray<br>RNA-seq	Direct
Subtract mRNA changes from protein changes	Indirect estimation
**Polysome Profiling**	**Direct**
