# Supplementary material for: Differential Sensitivity of Target Genes to Translational Repression by miR-17~92
Source: PLoS Genet. 2017 Feb 27;13(2):e1006623. doi: 10.1371/journal.pgen.1006623 (PMC5348049; doi:10.1371/journal.pgen.1006623)
Supplement: S1 Methods — (PDF) [file pgen.1006623.s031.pdf]

Differential sensitivity of target genes to translational repression by miR-17~92

Jin et al., PLOS Genetics (2017)
DOI: 10.1371/journal.pgen.1006623

Supplemental Experimental Procedures:

Northern blot and Probes

MiRNA Northern blotting was done as previously described [1, 2]. Briefly, 10~20μg total RNA extracted using TRIzol Reagent (Invitrogen, 15596-026) was separated on 10% denaturing polyacrylamide gels and electrotransferred to nylon membrane. DNA oligonucleotides antisense to mature miRNAs end-labeled with ^{32}P were used as probes. U6 snRNA was used as internal control. To determine the absolute copy numbers of miR-17~92 family miRNAs, graded amounts of chemically synthesized mature miRNAs (miR-17, miR-18a, miR-19b miR-92 and miR-155 from Dharmacon) were added into known numbers of TKO B cells before RNA extraction. These samples were compared with WT B cells by Northern blot, using a probe mix containing equal amounts of probes corresponding to each member of a miRNA subfamily. For example, to detect miR-17 subfamily miRNAs, an equal molar mixture of probes corresponding to miR-17-5p, miR-20a, miR-106a, miR-20b, miR-106b, and miR-93 was used. The sequences of probes used in this study are:

miRNA subfamily	Mature miRNAs	Northern blot probes (Seed sequences are underlined)
miR-17 subfamily:	mmu-miR-17-5p	ACT ACC TGC ACT GTA A*GC ACT TT*G
	mmu-miR-20a	CTA CCT GCA CTA TAA *GCA CTT T*A
	mmu-miR-106a	CTA CCT GCA CTG TTA *GCA CTT T*G
	mmu-miR-20b	CTA CCT GCA CTA TGA *GCA CTT T*G
	mmu-miR-106b	ATC TGC ACT GTC A*GC ACT TT*A

	mmu-miR-93	CTA CCT GCA CGA ACA *GCA CTT T*G
miR-18 subfamily:	mmu-miR-18a	CTA TCT GCA CTA GAT *GCA CCT T*A
	mmu-miR-18b	CTA ACA GCA CTA GAT *GCA CCT T*A
miR-19 subfamily:	mmu-miR-19a	TCA GTT TTG CAT AGA *TTT GCA C*A
	mmu-miR-19b	TCA GTT TTG CAT GGA *TTT GCA C*A
miR-92 subfamily:	mmu-miR-92	CAG GCC GGG ACA A*GT GCA AT*A
	mmu-miR-363	TAC AGA TGG ATA CC*G TGC AAT* T
	mmu-miR-25	TCA GAC CGA GAC AA*G TGC AAT* G
miR-155	mmu-miR -155	CCC CTA TCA CAA TT*A GCA TTA* A
U6 snRNA (control)		TAT GTG CTG CCG AAG CGA GCA C

Immunoblot and Antibodies

B cells were lysed in buffer containing 1% Nonidet P-40, 150 mM NaCl, 50 mM Tris-Cl (pH 8.0), 1 mM sodium orthovanadate, 1 mM DTT and proteinase/phosphatase inhibitors (Thermo, #78442). 10 μg protein lysates were subjected to 8% - 16 % SDS-PAGE and Immunoblot, depending on molecular weight of genes of interest. Primary antibodies were incubated overnight at 4°C. Antibodies used for Immunoblot in this study are:

Providers	Gene name and catalog number
Cell Signaling	anti-4EBP1 (cst-9644), anti-4EBP2 (cst-2845), anti-Bim (cst-2933), anti-Cul3 (cst-2759), anti-eEF2 (cst-2332), anti-eIF2A (cst-9722), anti-eIF4A (cst-2013), anti-eIF4B (cst-3592), anti-eIF4E (cst-2067), anti-eIF4G (cst-2498), anti-eIF5 (cst-2480), anti-Irf4 (cst-4694), anti-Jak2 (cst-3230), anti-mTOR (cst-2983), anti-MAP3K3 (MEKK3) (cst-5727), anti-Mnk1 (cst-

	2195), anti-Pabp1 (cst-4992), anti-Pdcd4 (cst-9535), anti-Phlda3 (cst-4294), anti-Pten (cst-9559), anti-Smad4 (cst-9515), anti-Socs3 (cst-9172), anti-RhoB (cst-2098), anti-Traf3 (cst-4729), anti-S6 (cst-2317), anti-Trim33 (cst-8972), phospho-Erk (MAPK44/42, cst-9101), and anti-phospho-S6 (cst-4856)
Santa Cruz Biotechnology	anti-Cyld (sc-137139), anti-Tnfaip1(A20) (sc-16692), anti-Stk38 (Ndr1) (sc-46184), anti-Dusp10 (sc-135201), anti-Mcl1 (sc-819), anti-Mnk2 (sc-6964), anti-Ccnd2 (sc-593), anti-c-Rel (sc-71), anti-E2f1 (sc-193), anti-E2f2 (sc-633), anti-Ikzf1(Ikaros) (sc-9859), anti-E2f3 (sc-879), anti-Rb1 (sc-50), and anti-Akt1/2/3 (sc-8312)
Abcam	anti-Rnf11 (ab57180), anti-Tax1bp1 (ab22049), anti-Cul2 (ab2135) and anti-Dusp2 (ab95159)
Bethyl Laboratories	anti-Phlpp2 (A300-661A-1), anti-Lats2 (A200-479A), anti-Mink1 (A302-191A), anti-Rapgef2 (PDZ-GEF1) (A301-966A), anti-Sos1 (A301-890A), anti-Tmem127 (A303-450A) and anti-Arid4b (A302-233A).
Sigma-Aldrich	anti-Tsg101 (T5701) and anti-β-Actin (A2228).
Novus Biologicals	anti-Casp11/Casp4 (NB120-10454), anti-eIF3C (NB100-511) and anti-Jarid2 (NB100-2214)
Others	anti-Hbp1 (Proteintech, 11746-1-AP), anti-Itch (BD Transduction Laboratories, 611199), anti-Dusp7 (Bioss, 7928R), anti-Rbbp8 (CtIP) (Active Motif 61141), anti-Socs1 (MBL, K0175-3), and anti-Cdkn1a (P21) (BD Pharmingen, 556430).

Flow Cytometry

Cell surface staining and flow cytometric analysis were performed following established protocols. Stained cells were analyzed on FACSCalibur or LSR II (BD Biosciences). Data were analyzed with FlowJo software (Tree Star, Ashland, OR). Antibodies and reagents with the following specificities were used for staining: anti-CD19 (1D3, BD Bioscience), anti-Tgfbr2 (FAB532A, R&D Systems) and anti-CD69 (104508, BioLegend)

Quantitative RT-PCR and Primers

B cells were purified as described. Total RNA was extracted using TRIzol Reagent (Invitrogen, 15596-026). The first cDNA strand was synthesized by reverse transcription using a primer mix of oligo-dT and random hexamers following manufacturer's instructions (Bio-Rad 170-8890) and used for quantitative real time PCR (Thermo Scientific, K0222) following the manufacturer's protocol. Analysis was performed on technical triplicates for each biological sample. β-Actin (*Actb*) was used for normalization. hRluc and Fluc primers are modified from a previous publication [3]. The following primers were used in this study:

Gene name	Forward primers	Reverse primers
Actb	5'-CTA AGG CCA ACC GTG AAA G- 3'	5'-ACC AGA GGC ATA CAG GGA CA -3'
Pten	5'-TGG GGA AGT AAG GAC CAG AG-3'	5'-GGC AGA CCA CAA ACT GAG GA-3'
Phlpp2	5'-ATC TGG TCA GGA GAC TGG A-3'	5'-GGA TTC GAT CCA GAT GAT CCA-3'
Cyld	5'-TGG GAT GGA AGG TTT GAT GG-3'	5'-CAT AAA GGC AAG TTT GGG AGG-3'
Itch	5'-AAC ATT CCC TCC GAA ATT ACC-3'	5'-CAT CAA TAA GAC AGC AAA CCT G-3'
Tnfaip3 (A20)	5'-TTT AAA TTC CGC TGG CAG C-3'	5'-ATT CCA GTT CCG AGT GTC G-3'
Traf3	5'-AGG AGA AGT TTG TGA AGA CGG-3'	5'-TGT GCA TTT TGG ACT GGA GG-3'
Sfpi1 (PU.1)	5'-GTC TGA TGG AGA AGC TGA TGG-3'	5'-AGA TGC TGT CCT TCA TGT CG-3'

Aicda (AID)	5'-TCG GGA TCA TGA CCT TCA AAG-3'	5'-TCG TAC AAG GGC AAA AGG ATG-3'
CD69	5'-GAC ATG GAA ATG GGC AAA TGG-3'	5'-CGT CAT CTG GAG GGC TTG-3'
Tax1bp1	5'-TGC CAT TAC ACC TTA ACT CCG-3'	5'-CAA TTG ACT GTT GAT CCT TCC AC-3'
Stk38 (NDR1)	5'-AGG AGC ATT TGG TGA GGT G-3'	5'-CTG CCT CCA CTA GAA TGT CAC-3'
Arid4b	5'-ACA GCA AGT CTC CAG CAA G-3'	5'-CAG CAC TTG TCA TAT TTT CCA GG-3'
Rnf11	5'-AAC TGC CTC AAA TCC CCG-3'	5'-TGA TAG ATG GGA ACA GGA ACT TG-3'
Rbbp8 (CtIP)	5'-CCA AGC AAC CAA GAT ACG TCC-3'	5'-TCC ATG TCC ACT GTT TCT CC-3'
Ikzf1 (IKAROS)	5'- AGT TCT CAT CCC ATA TCA CGC-3'	5'- GCA AAT CCA CTC CCA ACA TTG-3'
Hbp1	5'- CGT GAA GAT GAG GTG GAC TG-3'	5'- GGC GTG CAT AGG AAT GTA AAC-3'
hRluc	5'- TGC AAG CAA ATG AAC GTG CTG GAC -3'	5'- ATT CCG ATC AGA TCA GGG ATG ATG -3'
Fluc	5'- CTG CTA ACG ACA TTT ACA ACG AGC -3'	5'- CGA ATG TGT ACA TGC TCT GGA AGC -3'

Absolute quantification mRNA abundance (ERCC-RNA-seq)

RNA extraction and ERCC control spike-in

Activated WT B cells were prepared as described. 3 x 10^6 cells were harvested at indicated time points (naïve, 1.5h, 4.5h, 13.5hr and 25.5hr). We determined the amount of ERCC control needed to cover the whole dynamic range of endogenous mRNA abundance at each time point in a pilot experiment, and added the following amounts of ERCC control into 3 x 10^6 cells before RNA extraction: naïve, 1uL or 0.1uL; 1.5h and 4.5h, 0.5uL; 13.5hr, 1uL; and 25.5hr, 2uL. Note that ERCC control contains a mix of 92 exogenous RNAs of various length and GC content as well as abundance that spans six orders of magnitude [4]. Total RNA was extracted using the RNeasy Mini Kit (Qiagen, 74104) according to manufacturer's instructions and poly(A) containing mRNA species were further isolated before sequencing analysis.

RNA deep sequencing and data analysis

100bp reads were generated by the HISeq Analyzer 2000 at the TSRI Next Generation Sequencing Core. The Genome Analyzer Pipeline Software (currently Casava v1.8.2) was used to perform the early data analysis of a sequencing run, which does the image analysis, base calling, and demultiplexing. The program called cutadapt (http://journal.embnet.org/index.php/embnetjournal/article/view/200/479) was used to trim the adapter and low-quality ends. The remaining reads shorter than 35bp were filtered out. For mRNA-Seq alignment, TopHat v2.0.8b (http://tophat.cbcb.umd.edu/) with Bowtie2 was used to align the reads to genome using the genome reference (mouse mm10) with very sensitive alignment and other default parameters. Bowtie2 was used to align the reads to the ERCC sequences and then the coverage was quantitated with bedtools (http://code.google.com/p/bedtools/) software. Partek Genomics Suite 6.6 (Partek Inc., St. Louis, MO, USA, http://www.partek.com/) was used to quantitate the exon/gene counts. Linear model fitting plots of log2 RPKM values of ERCC to log2 ERCC copy numbers were further generated with ggplot2 packages in R, using detected ERCC control mix in each replicates separately. Log2 RPKM values of endogenous mRNA were directly converted to log2 copy number per cell using the equation of the plot. Genes were discarded from analysis when the calculated mRNA copy number per cell was lower then 0.5.

Microarray

B cells were purified and activated as described. Total RNA was extracted using the RNeasy Mini Kit (Qiagen, 74104). Microarray analysis was performed at the TSRI DNA Array Core Facility using the Affymetrix GeneChip Mouse Gene 1.0 ST Array or the Affymetrix GeneChip Mouse Gene 1.1 ST Array. Data normalization was performed using RMA Express 1.0 (http://rmaexpress.bmbolstad.com) with quantile normalization, median polish and background adjustment. The Limma package in the R

software was used to find transcripts with differential expression. The fold changes and standard errors were estimated by fitting a linear model for each gene and empirical Bayes smoothing was applied to the standard errors. Results are presented between two or more experimental conditions as a fold change in expression level, the moderated t-statistic, the p-value, and the adjusted p-value. The adjusted p-value is the p-value adjusted for multiple testing using the Benjamini and Hochberg's method to control the false discovery rate of 0.1 or less. The transcripts identified as differentially expressed were those with fold changes more than 1.4 fold. Microarray data reported in the paper have been deposited in NCBI Gene Experssion Omnibus. The GEO accession number is GSE56379.

Polysome profiling and Poly-RNA-seq analysis of primary B cells

0.8 -1.2 x 10^8 B cells were purified from 6-8 mice and activated with LPS and IL-4 for 25.5 hours as described. Fifteen minutes before harvesting, cells were treated with 100µg/ml of cyclohexamide (CHX) to block ribosome elongation during extraction preparation. Cells were first swelled in 10ml of hypotonic buffer (1.5ml KCl, 10mM MgCl2, 5mM Tris-HCl pH 7.4, and 100µg/ml cyclohexamide), and cytosolic fractions were extracted with total 600ml of 1:1 mix of hypotonic buffer and hypotonic lysis buffer (2% sodium deoxycholate, 2% Triton X-100, 2.5mM DTT, 10 units of RNase Inhibitors/ml, and 100µg/ml cyclohexamide). After 20 minutes of incubation on ice, nuclei were removed by centrifugation at 2,300g for 10 minutes. Supernatant was centrifuged through 15%-45% (w/w) of RNase inhibitor-containing sucrose gradients at 40,000 rpm for 1.5 hours using a Beckman SW41 rotor. The resulting gradient was separated into 20 fractions from top and A254 was monitored with recording spectrophotometer in parallel. Equal amounts of exogenous RNA control (Thermo Scientific, Solaris RNA spike control, K-002200-C1) were directly added into each fraction, and total RNA was extracted using TRIzol-LS solution following manufacturer's instructions. The first cDNA strand was synthesized by reverse transcription using a primer mix of oligo-dT and random hexamers

following manufacturer's instructions (Bio-Rad 170-8890). Quantitative PCR was performed as described. CT values were normalized to the spiked-in Solaris RNA to control for any technical variations that might occur during RNA extraction, cDNA synthesis, and PCR steps. Smoothing lines were generated using Prism (GraphPad Software Inc) with smoothing option (two neighbor to average and 2nd order of smoothing polynomial) using the average "% of total" value of technical replicates generated from a representative biological replicate.

RNA-seq analysis of polysome profiling samples (Roly-RNA-seq) were prepared in a same way, but only fraction 10-11 (corresponding to fractions associated with 3-4 ribosomes) and fraction 14-16 (corresponding to more than 7 ribosomes) were collected for calculating RPKM ratio of fraction 10-11 to fraction 14-16 of miRNA targets. Total RNA was extracted using Trizol-LS solution according to manufacturer's instruction and RNA abundance was calculated as described.

miRNA qRT-PCR

Total RNA from each sucrose gradient fraction was extracted as described above. After total RNA were eluted using the same volume of water for each fraction, equal volume of extracted RNA from each fraction was used to synthesize miRNA specific cDNA following manufacturer's instructions (Applied Biosystem, 4366596) with the following RT primers (Applied Biosystem, 4437975: miR-17, ID-002308; miR-18a, ID-002422; miR-19b, ID-000396; miR-92, ID-000430; let-7a, ID-000377; and miR-21, ID-000397). Equal volumes of the RT reaction products were used for quantitative PCR following manufacturer's instructions (Applied Biosystem, 4440040) using RT-primer matched Taqman Small RNA assay (Applied Biosystem, 4427975).

Ribosome profiling analysis of primary B cells

<u>Ribosome footprint library generation</u>

Ribosome footprint library were generated as previously described with modifications [5, 6]. Major modifications are: (1) Used RNA purified from cytosolic fractions, instead of whole cell lysate with DNase treatment procedure [6]. (2) RNase I treated footprints were purified using sucrose gradient and excluded disome fractions [5], instead of the sucrose cushion method [6]. (3) rRNAs were depleted using rRNA depletion kit (Epicentre RZH110424 or Epicentre MRZH11124) after footprint recovery and before the dephosphorylation and linker ligation steps. Indexed footprint library was generated with 4 or 6 cycles of PCR using index library primers, and avoided broad, slower migrating smear bands [6]. Numbers of cycles were increased only when 4 to 6 cycles did not give enough PCR products for downstream sequencing analysis.

Index library primers used in this study [6]:

Barcode_Reverse_01_ACGACT 5'-

caagcagaagacggcatacgagatAGTCGTgtgactggagttcagacgtgtgctcttccgatct-3'

Barcode_Reverse_02_ATCAGT 5'-

caagcagaagacggcatacgagatACTGATgtgactggagttcagacgtgtgctcttccgatct-3'

Barcode_Reverse_03_CAGCAT 5'-

caagcagaagacggcatacgagatATGCTGgtgactggagttcagacgtgtgctcttccgatct-3'

Barcode_Reverse_04_CGACGT 5'-

caagcagaagacggcatacgagatACGTCGgtgactggagttcagacgtgtgctcttccgatct-3'

Barcode_Reverse_05_GCAGCT 5'-

caagcagaagacggcatacgagatAGCTGCgtgactggagttcagacgtgtgctcttccgatct-3'

Barcode_Reverse_06_TACGAT 5'-

caagcagaagacggcatacgagatATCGTAgtgactggagttcagacgtgtgctcttccgatct-3'

Barcode_Reverse_07_CTGACG 5'-

caagcagaagacggcatacgagatCGTCAGgtgactggagttcagacgtgtgctcttccgatct-3'

Barcode_Reverse_08_GCTACG 5'-

caagcagaagacggcatacgagatCGTAGCgtgactggagttcagacgtgtgctcttccgatct-3'

Barcode_Reverse_09_TGCAGC 5'-

caagcagaagacggcatacgagatGCTGCAgtgactggagttcagacgtgtgctcttccgatct-3'

Differential expression analysis of ribosome footprints

We followed the previously described analysis scheme [6]. Briefly, the raw sequencing data were separated into individual samples using index primer sequences. Then, the miRNA linker and the first nucleotide of 5'-end are clipped and trimmed using fastx toolkit (http://hannonlab.cshl.edu/fastx_toolkit/). Reads obtained from rRNAs are discarded using Bowtie v1.0.0 using 23 seed flag [7]. Illumina iGenome (mm10) were used as a reference. The non-rRNA sequencing reads were further aligned to the reference genome (mm10) using TopHat v2.0.11 with added option, "no-novel-juncs" [8]. Only the perfect-match aligned reads were further extracted from accepted hits (accepted_hits.bam) to reduce potential noise derived from relatively short ribosome footprint length [6]. Differential ribosome footprint levels in different genotypes were further quantified using Cuffdiff (v 2.2.1) [9, 10]. To accept only translated genes, we took cut-off of ribosome footprints at fragments per kilobase per million (FPKM) > 1, and only selected genes that were quantified in all three biological replicates. This cut-off includes all 48 target proteins detected in Immunoblot or FACS analysis (A list of targets detected and quantified by immunoblot and FACS can be found in **Supplemental Table 3**).

Ribosome footprint coverage plot

Footprint reads were aligned to exon regions of a unique, canonical transcript defined by KnownCanonical geneset from UCSC genome browser using CoverageBed operation of BEDtools suite [11]. "-split" flag was used to include reads that were split by spliced regions. Coverage values of

each nucleotide position was divided by read counts of a given gene of individual samples to normalize area under footprint coverage to reveal ribosome distribution changes inter-gene levels independent from overall ribosome quantity changes found in miRNA target genes among different genotypes [12]. They were further aligned by start codon or stop codon and averaged values of biological triplicates of each nucleotide position of given gene was compared. Coverage plot of a set of genes (i.e. 641 miR-17~92 targets) are generated from trimmed average (10%) values of the coverage values from each nucleotide position.

Estimation of relative contribution of translation changes and mRNA changes, and calculation of ribosome density

Impact of miRNAs on transcribed target mRNAs were directly measured by comparing relative mRNA abundance determined by microarray (log2[$mRNA_{miR}$/$mRNA_{WT}$]). Impact of miRNAs on translation efficiency (termed Translation) was estimated by comparing normalized ribosome footprint abundance (FPKM) to the mRNA level changes (log2[$Ribo_{miR}$/$Ribo_{WT}$] – log2[$mRNA_{miR}$/$mRNA_{WT}$]). Ribosome density of WT were calculated by comparing the normalized ribosome footprint abundance (FPKM) from ribosome profiling to absolute mRNA levels from ERCC-RNA-seq in WT B cells (log2[$Ribo_{WT}$/$mRNAcopy_{WT}$]). Ribosome density of other genotypes (i.e. TKO or TG) was estimated by relative mRNA changes between different genotypes and normalized ribosome footprint abundance (FPKM) measured by Ribosome profiling in different genotype (log2[$Ribo_{miR}$/$mRNAcopy_{WT}$]-log2[$mRNA_{miR}$/$mRNA_{WT}$]).

Calculation of 5'UTR, CDS and 3'UTR length

The reference sequence (RefSeq) database including gene accession ID and exon start/stop sites were downloaded from UCSC genome browser (http://genome.ucsc.edu/, Assembly Hg19). To analyze length of UTR and CDS of messenger RNAs, datasets only starting from prefix 'MN_' were extracted.

Then the length of each exon was summated if single accession ID consists of multiple exon regions. When multiple RefSeq accession ID corresponds to single gene, only a representative value was selected for a unique gene value. The resulting length distribution was plotted using Prism (Graph Pad Software Inc) or “ggplot2” package in “R”.

5’UTR GC contents and structures

Murine 5’UTR sequences were retrieved from Ensembl BioMart database (Ensembl Genes78, Mus musculus). Aligned 5’UTR sequence from its 3’-end, cropped the 100 nucleotides (nts), and discarded sequences longer then 100nt. Occurrence of GC% of given genesets was calculated in each nucleotide position and generated loess smooth line using ggplot2 package in R with stat_smooth() function (span=0.2).

5’UTR secondary structures and free energy of nucleotide positions were predicted by directly querying the whole 5’UTR sequences of individual mRNAs into RNAfold. The centeroid secondary structure was presented in the figure (http://rna.tbi.univie.ac.at/cgi-bin/RNAfold.cgi) [13]. The murine CD69 5’UTR sequence used in this study is:

Murine CD69 5’UTR (ENSMUSG00000030156)

5’ACUGAACAAGACAGCUCCAGCUACAUCUCUCCGUGGACCACUUGAGAGUCGCCAGGG
ACCUUGAGGGGAAAAAAAUUAAAAAGG-3’

Cloning of the 3’UTR and 5’UTR of target genes

Fragments of target 3’UTRs were cloned into psiCHECK^{TM}-2 Vector using the XhoI and NotI enzyme sites in the multiple cloning region and miR-17~92 binding sites were mutated as previously described [14]. For *CD69,* the murine *CD69* 5’UTR and its variants were additionally clone into a position downstream to the SV40 promoter and upstream of hRluc, thereby generating a hRluc reporter gene

flanked by the engineered 5’UTR and 3’UTR. The sequence downstream to the transcription start site (TSS) of the SV40 promoter was trimmed to minimize the number of additional nucleotides in the engineered 5’UTRs. The TATA box and Kozak sequence remain intact. The 5’UTR sequences were synthesized by IDT (Integrated DNA technology) and cloned into the psiCheck2 vector.

Cloned 3’UTR

Underlined sequences and bolded sequence indicate miR-17~92 binding sites and their mutations, respectively.

CD69 (936 nts):

TAACTGGATACTGCTCTGCAGTTGAATTTTCCACAAAGACTGCACAAACCAACTTTACATC
ATCCTGGATCAACTCCAGATAGAACTGTGGGACATGAGGAAGAAGATCCAGGAAGATCC
CTTGACCAGGAGCTGAAACTGTCACCAACTGACTGCTAATCACATCAAGGATGAGAGGAT
GGCTATGAAGCCTCATCAAC<u>GCACTTT</u>ATTTTTTTTTAAACCTAGAATGAATAGAACTGAC
TAGCTCTAGAGTTACTATTTATTGCCGAATGACTGCTACAGCCAGTGCCTTTACGCATTTG
CACTATTTGGAGGGGTTTCAGTGGTAGGAAGAGCTGAATGTAGGTACAGGAAGATTTGAC
TGATTAATGATTTTCTTAAATTCAAAAAGCATTCACAAATAGAACAATGCTTATGAAACC
AAGCTTTGCAGTAACTCCATCTATTTACAGAAACTTTGCCTCATTATTTTGTCAATTGTTCT
CCCCCAAAGAATAAGAGTGGCCTTTTCTTTAACTTCCTCCGTGTAGACAGCTAATTTTCAA
TGGTACATATTTCTTAACCTTTAAAAACCTCTGTAGCGTATTTCAAGGAAGAAAACAAAA
GCACAGCATATGAGTAATATTTGTAGAGCAGATTTCAAAGTGCTGGAAAGAAAA<u>GTGCAA</u>
<u>T</u>ACGTGTAGTGGCAGATCTCTGTCAGGACACACCCTGTGGTTTGACCTTGGAATAACTCCA
GTCTTGTCACATAACCTGTTTGTGTCTTTCCCAGTCCTTTGTCCAAAATATTTCCCAT<u>GTGC</u>
<u>AAT</u>AAAGTGTTTATATATTTGTTTGTGTTTTAAAACCCACTATAAAGTTTAAGCTGTTTC

Mutated CD69 (936 nts):

TAACTGGATACTGCTCTGCAGTTGAATTTTCCACAAAGACTGCACAAACCAACTTTACATC

ATCCTGGATCAACTCCAGATAGAACTGTGGGACATGAGGAAGAAGATCCAGGAAGATCC

CTTGACCAGGAGCTGAAACTGTCACCAACTGACTGCTAATCACATCAAGGATGAGAGGAT

GGCTATGAAGCCTCATCAACG**A**A**G**T**C**TATTTTTTTTTAAACCTAGAATGAATAGAACTGA

CTAGCTCTAGAGTTACTATTTATTGCCGAATGACTGCTACAGCCAGTGCCTTTACGCATTT

GCACTATTTGGAGGGGTTTCAGTGGTAGGAAGAGCTGAATGTAGGTACAGGAAGATTTGA

CTGATTAATGATTTTCTTAAATTCAAAAAGCATTCACAAATAGAACAATGCTTATGAAACC

AAGCTTTGCAGTAACTCCATCTATTTACAGAAACTTTGCCTCATTATTTTGTCAATTGTTCT

CCCCCAAAGAATAAGAGTGGCCTTTTCTTTAACTTCCTCCGTGTAGACAGCTAATTTTCAA

TGGTACATATTTCTTAACCTTTAAAAACCTCTGTAGCGTATTTCAAGGAAGAAAACAAAA

GCACAGCATATGAGTAATATTTGTAGAGCAGATTTCAAAGTGCTGGAAAGAAAAG**C**G**A**A

GTACGTGTAGTGGCAGATCTCTGTCAGGACACACCCTGTGGTTTGACCTTGGAATAACTCC

AGTCTTGTCACATAACCTGTTTGTGTCTTTCCCAGTCCTTTGTCCAAAATATTTCCCATG**C**G

AA**G**TAAAGTGTTTATATATTTGTTTGTGTTTTAAAACCCACTATAAAGTTTAAGCTGTTTC

Lats2 (635 nts):

CTTTTCAGAAGACAAACTCAAGCTTAGGAATCCTTCATTTTTAGTTCTGGTAAATGGGCAA

CAGGAAGAGTCAACATGATTTCAAATTAGCCCTCTGAGGACCTTCACTGCATTAAAACAG

TATTTTTAAAAAATTAGTACAGTATGGAAAGAGCACTTATTTTGTGGATACCCATCTTTTT

CTTACTAAATTATAAGGACTGACTGTGAGAAACCATGCTGCTGTTATTTCCATGTGTGTTG

TATCTGATAGCACTTGTCCACAGCTAGAAAAGGAAGAGCTGGAGAGCGTGAGGCAAGAC

GTCTGTGCAATAAGAGAGGATGAGGCGACGGAGCTCTGCTCAAGTCACGAGGACCGCTTA

TCTACACAGTGGCTTAGTTTTGTATTTTCCCACATTTCAAATTGTGATGTAATGTTTGAAAG
CTGCTTTTGTATTTTCTCCTTTGCATATTATAGTTCCTAGAAAGAGTGAGCAGAGAGCTGG
TGGGTGTGACTCCGGTGTCTGGTGTGGAGAGTACTGCATGAGCAGGGGTTTCTAGTATAA
AATACCGTATCTTGCCATTCACAGCAGGTCCTGTGAATACGTTTTTAAATGAGGTATTCTA
GACAGTGTGCTGATAATGTATTGTGTGGA

Mutated Lats2 (635 nts):

CTTTTCAGAAGACAAACTCAAGCTTAGGAATCCTTCATTTTTAGTTCTGGTAAATGGGCAA
CAGGAAGAGTCAACATGATTTCAAATTAGCCCTCTGAGGACCTTCACTGCATTAAAACAG
TATTTTTAAAAAATTAGTACAGTATGGAAAG<u>A**C**C**T**C**A**T</u>ATTTTGTGGATACCCATCTTTTT
CTTACTAAATTATAAGGACTGACTGTGAGAAACCATGCTGCTGTTATTTCCATGTGTGTTG
TATCTGAT<u>A**C**C**T**C**A**T</u>GTCCACAGCTAGAAAAGGAAGAGCTGGAGAGCGTGAGGCAAGAC
GTCT<u>G**A**G**G**A**T**T**T**</u>AGAGAGGATGAGGCGACGGAGCTCTGCTCAAGTCACGAGGACCGCTT
ATCTACACAGTGGCTTAGTTTTGTATTTTCCCACATTTCAAATTGTGATGTAATGTTTGAAA
GCTGCTTTTGTATTTTCTCCTTTGCATATTATAGTTCCTAGAAAGAGTGAGCAGAGAGCTG
GTGGGTGTGACTCCGGTGTCTGGTGTGGAGAGTACTGCATGAGCAGGGGTTTCTAGTATA
AAATACCGTATCTTGCCATTCACAGCAGGTCCTGTGAATACGTTTTTAAATGAGGTATTCT
AGACAGTGTGCTGATAATGTATTGTGTGGA

Rb1 (669 nts):

GAAACAGATTGTTACAAACTGGGAAAAAGTACTAATTTCTACACATTGGACTATTTTAAT
ATTAGAATCTAATGTGCCGTGTGTTTGTTTCACATATTTTACTTTTGAAATACAAGCAAAA
AAAGTATAATCATATGATACTGTCTTACTACTGACACAGATTTCATACCTCAGACCCTCTA
AGAACCGATTCTTTTATTCACCCAACACATGCTTTGAACTGAAGACTATTGATAATACTCC
GAGGTTGTTTTTTTCTTTCAATCATTCAGATCACTGAATTTATAAGTACCCATGTAGTACTT

GAAAGTCAAGTTTGGCCACAACTGTGCTTAAGAGGACCCTAGTACAGTACAACCCAAGTG
CACTTTTTAATGTTTCTGGGTCCTGAAGAATCAAGATACAAATTAATTGTGATTTACAAGC
AGACTGTTAACTATAGAAGCCTTCAGTTTTTTTCCCTCATAGACGTGTCTAATTACATCTC
AACAGTTTACTCTGTTCTTCTACATCTGGGGATGTTTGTGTTCTCTCTGGAATGGTACATCT
TCCAGGGTCTTTTGAACTTGCAGTTATCTATTTTTTAAGCCAATCTGGTCTAATAACTCTGG
CTTCTTCAAAGCCACACCATTTCTAGTCCAGCTGTGCAGAAACTTCAGATGAAAACAG

Rb1 (669 nts):

GAAACAGATTGTTACAAACTGGGAAAAAGTACTAATTTCTACACATTGGACTATTTTAAT
ATTAGAATCTAATGTGCCGTGTGTTTGTTTCACATATTTTACTTTTGAAATACAAGCAAAA
AAAGTATAATCATATGATACTGTCTTACTACTGACACAGATTTCATACCTCAGACCCTCTA
AGAACCGATTCTTTTATTCACCCAACACATGCTTTGAACTGAAGACTATTGATAATACTCC
GAGGTTGTTTTTTTCTTTCAATCATTCAGATCACTGAATTTATAAGTACCCATGTAGTACTT
GAAAGTCAAGTTTGGCCACAACTGTGCTTAAGAGGACCCTAGTACAGTACAACCCAAGTG
GA**G**T**A**TTTAATGTTTCTGGGTCCTGAAGAATCAAGATACAAATTAATTGTGATTTACAAG
CAGACTGTTAACTATAGAAGCCTTCAGTTTTTTTCCCTCATAGACGTGTCTAATTACATCTC
AACAGTTTACTCTGTTCTTCTACATCTGGGGATGTTTGTGTTCTCTCTGGAATGGTACATCT
TCCAGGGTCTTTTGAACTTGCAGTTATCTATTTTTTAAGCCAATCTGGTCTAATAACTCTGG
CTTCTTCAAAGCCACACCATTTCTAGTCCAGCTGTGCAGAAACTTCAGATGAAAACAG

Sos1 (619 nts):

TTCCTCTAAGCTGGGATAGTTTCCTAGCCCCCAGATCCATTGCTGGCAATGGATGCACTGA
ACATGCCAGCACTGGGGAGTTCAAATGAGAACTCCAAACACTAACGACTCTACTTCACGA
TGTAGTATAAGACAATGAGTTTTAACCTACATGGAATTATGGAATAAAATGGTATTCCAG
CTTAGAATGTGGAAACTGATTGCACCTGGAAATCACGTGAAGGGACTTTTCTGGCCATTG

GGCAGGAGTCCTCATATTGTGAAGTGATCTTTATCATTAAAGGGATGGAAAACAGTCTAA

TGTCCAACAAGCCCATATGTTGACAGTTTTTGTAATTCAAAATATTAT<u>GCACTTT</u>TAAAAA

ATCTTAAACAGGGATCTCCTCCTTTGTTTTCCTTTGCTTTACTCTTCTACTTTAGAATATTTT

CGTAAAAGTTATTCAGAGGACTGTGAGAAAAGGCTGTGGTACCTGACCTTGTTGAAATCA

AGGCCCAGCACTGTACTACAGTCCTGTTTACAGATTATTACAGTGATCTGAATGGGTACCG

AGGCTTCACCAAAGAGGTACTTTTTGTTATTGTTATTGTTTTAAGAATAATTATGCCAATTT

TAAGAACATCC

Mutated Sos1 (619 nts):

TTCCTCTAAGCTGGGATAGTTTCCTAGCCCCCAGATCCATTGCTGGCAATGGATGCACTGA

ACATGCCAGCACTGGGGAGTTCAAATGAGAACTCCAAACACTAACGACTCTACTTCACGA

TGTAGTATAAGACAATGAGTTTTAACCTACATGGAATTATGGAATAAAATGGTATTCCAG

CTTAGAATGTGGAAACTGA<u>T**A**G**G**A**G**</u>CTGGAAATCACGTGAAGGGACTTTTCTGGCCATTG

GGCAGGAGTCCTCATATTGTGAAGTGATCTTTATCATTAAAGGGATGGAAAACAGTCTAA

TGTCCAACAAGCCCATATGTTGACAGTTTTTGTAATTCAAAATATTAT<u>G**G**A**G**T**A**T</u>TAAAAA

ATCTTAAACAGGGATCTCCTCCTTTGTTTTCCTTTGCTTTACTCTTCTACTTTAGAATATTTT

CGTAAAAGTTATTCAGAGGACTGTGAGAAAAGGCTGTGGTACCTGACCTTGTTGAAATCA

AGGCCCAGCACTGTACTACAGTCCTGTTTACAGATTATTACAGTGATCTGAATGGGTACCG

AGGCTTCACCAAAGAGGTACTTTTTGTTATTGTTATTGTTTTAAGAATAATTATGCCAATTT

TAAGAACATCC

Dusp2 (535 nts):

ACAGCTCTGGCTTTGACTGACTCCTGGGGGAAGCTGCTGTGGCCCAGCTCCTCCCTCCTCA

TCTTGCCGTCCCACTGGGGTGCTAGGAAATCCAGGATGACGGCTGCTCTGATATGGTGCTC

TTCTGAGGTGGCATAAGGGCTGGCCCTTATTTGCTCTCCCTCCTTCGACTTGCAGAAATTA

ATTTAATTAATTAATTTACTATATTAAAGCCCTTGGTCACCCAAGGGCTCAGAAAACAAGC
TGTGACAAGCAGAAACCATGTCTAGGGGTGTGCAGCCCTTGACCCAAAGCTTAAGCCTTG
TGCTCCCAGGGGAGCCAGGAAGTGACGTGTGTGTCATGTTACGGACATCTGACTTTTGTGT
GTGTGTGTGTGGGCATCTCGCTGTAATTGGTGCTGAAAAGTTATTTGTGTTCAACTGACAT
TTAACGCTCTCTCCCCAACTTCCTCCCGGCCCTGTGGGCCAGGGAGGGGCGTTGGAAACA
GCACTTTATATTTATATAGAACATTGAGGTTGTGTCAATAAAAACAGTG

Mutated Dusp2 (535 nts):

ACAGCTCTGGCTTTGACTGACTCCTGGGGGAAGCTGCTGTGGCCCAGCTCCTCCCTCCTCA
TCTTGCCGTCCCACTGGGGTGCTAGGAAATCCAGGATGACGGCTGCTCTGATATGGTGCTC
TTCTGAGGTGGCATAAGGGCTGGCCCTTATTTGCTCTCCCTCCTTCGACTTGCAGAAATTA
ATTTAATTAATTAATTTACTATATTAAAGCCCTTGGTCACCCAAGGGCTCAGAAAACAAGC
TGTGACAAGCAGAAACCATGTCTAGGGGTGTGCAGCCCTTGACCCAAAGCTTAAGCCTTG
TGCTCCCAGGGGAGCCAGGAAGTGACGTGTGTGTCATGTTACGGACATCTGACTTTTGTGT
GTGTGTGTGTGGGCATCTCGCTGTAATTGGTGCTGAAAAGTTATTTGTGTTCAACTGACAT
TTAACGCTCTCTCCCCAACTTCCTCCCGGCCCTGTGGGCCAGGGAGGGGCGTTGGAAACA
G**G**A**G**T**A**T**T**TATTTATATAGAACATTGAGGTTGTGTCAATAAAAACAGTG

Rapgef2 (BS-1&2) (773 nts):

CTTCTTCTCCCCTTCTTCCTTTCCCCTTTGCATGTGAAATACTGTGAAGAAATTGCCCTGGC
ACTTTGCAGACTTGTTGCTTGAAATGCACAGCCCAGCAGCCCCTGAGCTGCTGCCTGCCAC
GTCACGCAGTATCATTCCAAATTCCAAGATCGTCACAAGGCGATTCCCTCTGGCGGCCCTT
CTCCATGCCTGGAAGGAGATTCCAATCTTCCTCTTAGATTTCAATCCAGTCCTAGCCCTCG
ATCTCATTTGGAAGATGAGAAAAGCTAGCCTTTGAACTACCCGGGGTCTCGAACCCACCA
AGGAAGACAAAGACTGATATGAAATCCTTTGAGTACAGTGCTTGTCCACTTGTTTACAAT

ATCCTTTAAAAAAAAATAAGTTTAAAGATTGTGTTCAGAGAGTAAATGTTATATCCATTTT

ATGATTACAGTATTATTTTGAACCTTAAGTAGGATTGCCAGCCTGGGTTTCTGAAAAACCA

AATATGCCGGACAGGGTTTGGCCGCACCAAGGGAAGACCTGGGCAGTGACCCTGTCTTCC

CATGTCCTTCTGGCCTTGCCCGTGAAGTGCCCTATCCCAGAAGCATCAAACGTTAGCCAAT

TACTTAAATACCAAGACTCCTCACCTGCCCCTTCCCCAGTGGGTGAGGCTCTAATGTAAAA

CAGTTTGCACATGGCCAGGGGAGGGAACTAGGACTTGTGTGTCCTGTCTGAGCCTTATGA

GGCAGGATGGTGTTATTTAAGAGTGTGTCCTGCTCAATTACAAT

Mutated Rapgef2 (BS-1&2) (773 nts):

CTTCTTCTCCCCTTCTTCCTTTCCCCTTTGCATGTGAAATACTGTGAAGAAATTGCCCTGG**G**

A**G**T**A**TGCAGACTTGTTGCTTGAAATGCACAGCCCAGCAGCCCCTGAGCTGCTGCCTGCCA

CGTCACGCAGTATCATTCCAAATTCCAAGATCGTCACAAGGCGATTCCCTCTGGCGGCCCT

TCTCCATGCCTGGAAGGAGATTCCAATCTTCCTCTTAGATTTCAATCCAGTCCTAGCCCTC

GATCTCATTTGGAAGATGAGAAAAGCTAGCCTTTGAACTACCCGGGGTCTCGAACCCACC

AAGGAAGACAAAGACTGATATGAAATCCTTTGAGTACAGTGCTTGTCCACTTGTTTACAA

TATCCTTTAAAAAAAAATAAGTTTAAAGATTGTGTTCAGAGAGTAAATGTTATATCCATTT

TATGATTACAGTATTATTTTGAACCTTAAGTAGGATTGCCAGCCTGGGTTTCTGAAAAACC

AAATATGCCGGACAGGGTTTGGCCGCACCAAGGGAAGACCTGGGCAGTGACCCTGTCTTC

CCATGTCCTTCTGGCCTTGCCCGTGAAGTGCCCTATCCCAGAAGCATCAAACGTTAGCCAA

TTACTTAAATACCAAGACTCCTCACCTGCCCCTTCCCCAGTGGGTGAGGCTCTAATGTAAA

ACAGT**A**T**C**C**T**CATGGCCAGGGGAGGGAACTAGGACTTGTGTGTCCTGTCTGAGCCTTATG

AGGCAGGATGGTGTTATTTAAGAGTGTGTCCTGCTCAATTACAAT

Rapgef2 (BS-3&4) (731 nts):

GGCACCAGAGAGTATGTCTTTAAACATGATTAAAGGTCACAAACATGGTTAAATACGCCA

GCCTTTGCAAAATGGTTAGTCACCAAAGGCTGACCTGTAAGTGGCTTTGGGGACGCTGCA

TATGGAGAAGGCCAAGTGTAGCGCCCGTCTGCTCATAGCTGCTGCTAGCCCTAGAATGAC

TGAAAATGACCCCGCCGGTCTATTCTTGTGTTGTT<u>TTTGCAC</u>AGACTCCGGAAAAGTGAAG

GCTGCCAATCCGAGTAGTACTCAGATGTGAGGGGCTGCTGATCTTGGATTTTTCCATTAAA

TTCAGCTGATCATATTGATCAGTAGATAAACGTAAATAGCTTCAAATTTTAAAGGTCGAAT

TGCAGTGTTTTTTCACTGTGTCAAACAATGTCAGTGCTTTATTTAATAATTCTCTTCTATGT

CATGGCGTTTGTCTACTTGTGTATCACATTGTCACTTACGCATTTGTAATTTTCCATGTAAT

ATGCATTATTGCCAGTTTTACTCTATAGGCTATGGACCTCATGTGCATATAGAAAGACAAA

CCTCTAGCTCTACCACAAA<u>TTGCAC</u>AGATGTTATCTAAGCATTAAGTAATTGTAGAACATA

GGACTGCTAATCTCAGTTCGCTCTGTGATGTCAAGTGCAGAATGTACAATTAACTGGTGAT

TTCCTCATGCTTTTGATACCACTTGTACCTGTATGTCTTTTAGAAAGACATTGGTGGAGTC

Mutated Rapgef2 (BS-3&4) (731 nts):

GGCACCAGAGAGTATGTCTTTAAACATGATTAAAGGTCACAAACATGGTTAAATACGCCA

GCCTTTGCAAAATGGTTAGTCACCAAAGGCTGACCTGTAAGTGGCTTTGGGGACGCTGCA

TATGGAGAAGGCCAAGTGTAGCGCCCGTCTGCTCATAGCTGCTGCTAGCCCTAGAATGAC

TGAAAATGACCCCGCCGGTCTATTCTTGTGTTGTT<u>T**A**T**C**C**T**C</u>AGACTCCGGAAAAGTGAAG

GCTGCCAATCCGAGTAGTACTCAGATGTGAGGGGCTGCTGATCTTGGATTTTTCCATTAAA

TTCAGCTGATCATATTGATCAGTAGATAAACGTAAATAGCTTCAAATTTTAAAGGTCGAAT

TGCAGTGTTTTTTCACTGTGTCAAACAATGTCAGTGCTTTATTTAATAATTCTCTTCTATGT

CATGGCGTTTGTCTACTTGTGTATCACATTGTCACTTACGCATTTGTAATTTTCCATGTAAT

ATGCATTATTGCCAGTTTTACTCTATAGGCTATGGACCTCATGTGCATATAGAAAGACAAA

CCTCTAGCTCTACCACAAA<u>T**A**G**G**A**G**</u>AGATGTTATCTAAGCATTAAGTAATTGTAGAACAT

AGGACTGCTAATCTCAGTTCGCTCTGTGATGTCAAGTGCAGAATGTACAATTAACTGGTG

ATTTCCTCATGCTTTTGATACCACTTGTACCTGTATGTCTTTTAGAAAGACATTGGTGGAGT
C

Mink1 (770 nts):

CGAGGCCCCTGGGCTGGGGCTCCCCACATGGACCCAGCTCTCTTCCCACAGCCAGACTAC
CCAGGCCGCCCCTGCCCTCCCACCCCCTTGGGCTTTTGCTTTTACTGGTTTGATTCACTGGA
GCCTGCTGGGAACGTGACCTCATATCCCTGAAGCTTTCGTGATCACGTGACCACCCTTTCC
CCCAACATTCTCAAAACTGTGCCTTTCCCCAACTTGTGGGGAATGACACAGCTTCCTTCCC
CTTACCAGGAATTGAGTGGGACTTGCTCCTCCCCTTTTCTCCACAGAAGAGGAGAGCGCTT
GGGGCTTGGACCCCTTACCCCATTGCTGCTGAATGAGCAGGGCCCTGACCCCTTTATTTGC
ACGCCAGGGGAGCCGGCTCCCCCCTTGAATGTACCAGACTCTGGGGGGGGGGTGTTCACT
GGGCCCTGGGTTCTGGAGGCGGGGCGGTCACCAGCCACTCCAGGGGCAGGGACCATTCCT
CATTTTCTGAAAGCACTTTAATGATTCCCCCCCAACCCCCAGGGAATGGAGGAGGGACCC
CATAGCCAAAACATTCCCCCCTTTCCTCCCCACTCCCACCCCTTCTAGCCTCTCCCCTTCCC
TAGAAGGAGAGAGCTCAGAGCTCCCAGCCTTTATCCTCCCTTGCTTGCATCTGTATATAGT
GTGAGCAGCAAGTAGCCCTTCTCCCTCCTGTATCCTTTCTCAATGTAGTGGCCTTGGATAT
ATCCCCTTTGTTAATAAAGACAATTCAACCAGCTTCCACC

Mutated Mink1 (770 nts):

CGAGGCCCCTGGGCTGGGGCTCCCCACATGGACCCAGCTCTCTTCCCACAGCCAGACTAC
CCAGGCCGCCCCTGCCCTCCCACCCCCTTGGGCTTTTGCTTTTACTGGTTTGATTCACTGGA
GCCTGCTGGGAACGTGACCTCATATCCCTGAAGCTTTCGTGATCACGTGACCACCCTTTCC
CCCAACATTCTCAAAACTGTGCCTTTCCCCAACTTGTGGGGAATGACACAGCTTCCTTCCC
CTTACCAGGAATTGAGTGGGACTTGCTCCTCCCCTTTTCTCCACAGAAGAGGAGAGCGCTT
GGGGCTTGGACCCCTTACCCCATTGCTGCTGAATGAGCAGGGCCCTGACCCCTTTAT**A**T**C**C

TCGCCAGGGGAGCCGGCTCCCCCCTTGAATGTACCAGACTCTGGGGGGGGGGTGTTCACT

GGGCCCTGGGTTCTGGAGGCGGGGCGGTCACCAGCCACTCCAGGGGCAGGGACCATTCCT

CATTTTCTGAAAG**G**A**G**T**A**TAATGATTCCCCCCAACCCCCAGGGAATGGAGGAGGGACCC

CATAGCCAAAACATTCCCCCCTTTCCTCCCCACTCCCACCCCTTCTAGCCTCTCCCCTTCCC

TAGAAGGAGAGAGCTCAGAGCTCCCAGCCTTTATCCTCCCTTGCTTGCATCTGTATATAGT

GTGAGCAGCAAGTAGCCCTTCTCCCTCCTGTATCCTTTCTCAATGTAGTGGCCTTGGATAT

ATCCCCTTTGTTAATAAAGACAATTCAACCAGCTTCCACC

Fbxw7 (BS-1) (707 nts):

AAAGCAGACATGATGAATTTTGTCCAACTGTGTAGACAATATACTCCCTACCCTTCCCCCT

GCGCAAAAAACAAAAACAAACAAACAAAAAAATGAAAAAAAAAAAACAGAAAAAAAAAAA

AGAGAAAAAAGAAAAGGAAAAAAATCCCTTGTACTCAGTGGTGCAGGATGTTGGCTTGG

GACAACAGACTGAAAAGACCTACAGACTAAGAAGGCAAGAAGAGACAAGAGACCGTAA

CTGACAGGAGGCGGCAGCTGTCGCATCTCGCAAAGGCCTCACTTGTGACTGAGGGGCAGC

TTGGCAAGACGACTCTCTAAATCCAACCAGGTGCAATTATTCTTTGTTTTCTTCTCCAGTG

GTCATTGAGCAGAGCTACATCAGCGTTGTTACCGTCACCTAGAAAGGAGTGGCAGTAATA

TCCAAACACGGGCTGCTTATCTTCTAATCAGAGCATCTGCAACAAACCGTCATTTTTCTGA

AGTGGAAAAGCTTAAAACAATTACTGTGAATTGTTTTTGTACAGTTATCATGAAGCTTTCT

TTTTTCTCTTTTTCCTGTTTCTCTTCTTTTCTTTTTTCTTTTTTTTTTTTGCCAACCATTGCCA

ATGTCAATCAATCACAGTATTAGCCTCTGTTAATCTATCTCTTTACTGTTGCTTCTACTCTT

CAATGCATATGTTGCTCAAAGGTGGCAAGTTGTCCTGGGTTG

Mutated Fbxw7 (BS-1) (707 nts):

AAAGCAGACATGATGAATTTTGTCCAACTGTGTAGACAATATACTCCCTACCCTTCCCCCT

GCGCAAAAAACAAAAACAAACAAACAAAAAAATGAAAAAAAAAAAACAGAAAAAAAAAAA

AGAGAAAAAAGAAAAGGAAAAAAATCCCTTGTACTCAGTGGTGCAGGATGTTGGCTTGG

GACAACAGACTGAAAAGACCTACAGACTAAGAAGGCAAGAAGAGACAAGAGACCGTAA

CTGACAGGAGGCGGCAGCTGTCGCATCTCGCAAAGGCCTCACTTGTGACTGAGGGGCAGC

TTGGCAAGACGACTCTCTAAATCCAACCAGG**A**G**G**A**T**TTATTCTTTGTTTTCTTCTCCAGTG

GTCATTGAGCAGAGCTACATCAGCGTTGTTACCGTCACCTAGAAAGGAGTGGCAGTAATA

TCCAAACACGGGCTGCTTATCTTCTAATCAGAGCATCTGCAACAAACCGTCATTTTTCTGA

AGTGGAAAAGCTTAAAACAATTACTGTGAATTGTTTTTGTACAGTTATCATGAAGCTTTCT

TTTTTCTCTTTTTCCTGTTTCTCTTCTTTTCTTTTTTCTTTTTTTTTTTTGCCAACCATTGCCA

ATGTCAATCAATCACAGTATTAGCCTCTGTTAATCTATCTCTTTACTGTTGCTTCTACTCTT

CAATGCATATGTTGCTCAAAGGTGGCAAGTTGTCCTGGGTTG

Fbxw7 (BS-2) (513 nts):

GAGGGCTGTGGTGAATGGAAGAACATACATAGCAAAACTGACAAGATATTTTAAAGATA

TATAAAACACAAAGGAAAAGGAGGTTGCTGGTCAGTCGTAGCATCTTACAGTATTGGGGA

AAACAACTGTTACAGTTTCATTGCTCTGAGTGACTGACGTGAGAGGAATTCGCTCTGCAGT

GACGCTGTCTGTCACTCGCCTACCAGCTCGACGAGCAAGAGAGCGGGAGTCAGATGGTCC

GCCTCATTCACCAGGAGCCGTAACTCAAGCTGAACTGTGAAAGTGGTTAACACTGTATCC

TAGGCCGTCTTTTTTTTCCTCCTCCTGTTTATTTTTTGTTTGTTTTATTTATAGTCTGATTTA

AAACAATCAGATTCAAGTTGGTTAATTTTAGTTATGTAACAACCTGACGTGATGGAGGAA

ACAACCTGTAAAGGGATTGTGTCTATGGTTTGATTCACTTAGAAATTTTATTTTCTTATAA

CTTAAGTGCAATAAAATGTGTTTTTT

CATGTTA

Mutated Fbxw7 (BS-2) (436 nts):

GAGGGCTGTGGTGAATGGAAGAACATACATAGCAAAACTGACAAGATATTTTAAAGATA

TATAAAACACAAAGGAAAAGGAGGTTGCTGGTCAGTCGTAGCATCTTACAGTATTGGGGA

AAACAACTGTTACAGTTTCATTGCTCTGAGTGACTGACGTGAGAGGAATTCGCTCTGCAGT

GACGCTGTCTGTCACTCGCCTACCAGCTCGACGAGCAAGAGAGCGGGAGTCAGATGGTCC

GCCTCATTCACCAGGAGCCGTAACTCAAGCTGAACTGTGAAAGTGGTTAACACTGTATCC

TAGGCCGTCTTTTTTTTCCTCCTCCTGTTTATTTTTTGTTTGTTTTATTTATAGTCTGATTTA

AAACAATCAGATTCAAGTTGGTTAATTTTAGTTATGTAACAACCTGACGTGATGGAGGAA

ACAACCTGTAAAGGGATTGTGTCTATGGTTTGATTCACTTAGAAATTTTATTTTCTTATAA

CTTAAG**A**G**G**A**T**TAAAATGTGTTTTTTCATGTTA

MAP3K3 (436 nts):

GGACATGCTGGCTCAGCATCCTCAGGACCAAGTTGTTGCTTAATTTATTGTTTTTTAATAA

CTAATCCAGATAAAAAGTTGTGGGGCTTCAGGGTGACCTGGGCCCAAAGGTTCTGAAGGG

CAGTTCCTGGCAGCCCCAGGCTTGCTGTGGGAAGGGGCCGTGCCGTCACTTTCTCATCATT

CCATGGGGTGTGTCTGCCTGGGCCAACTCTGCATGGAGAGGCCAGGGCTGGGGACAGTCC

GCACTCTGCCACCCTCCTGCCCCTTCCACCCACCCCAGCTCTATGTCTGTGTCTGAATTGTG

GATCGTGCAGCCATGGTTATTGTGGAACTGTGGAACCTGCAGCCATAGTTATTTGACTATA

TCTTGACCGAGGGCTTGCAGTGCAAAGCCAGGCCAGTGTTGCGCATTACTTACAATAAAA

GGGATCATTTA

Mutated MAP3K3 (436 nts):

GGACATGCTGGCTCAGCATCCTCAGGACCAAGTTGTTGCTTAATTTATTGTTTTTTAATAA

CTAATCCAGATAAAAAGTTGTGGGGCTTCAGGGTGACCTGGGCCCAAAGGTTCTGAAGGG

CAGTTCCTGGCAGCCCCAGGCTTGCTGTGGGAAGGGGCCGTGCCGTCACTTTCTCATCATT

CCATGGGGTGTGTCTGCCTGGGCCAACTCTGCATGGAGAGGCCAGGGCTGGGGACAGTCC

GCACTCTGCCACCCTCCTGCCCCTTCCACCCACCCCAGCTCTATGTCTGTGTCTGAATTGTG

GATCGTGCAGCCATGGTTATTGTGGAACTGTGGAACCTGCAGCCATAGTTATTTGACTATA

TCTTGACCGAGGGCTTGCAGTGCAAAGCCAG**C**C**G**A**C**TGTTGCGCATTACTTACAATAAAA

GGGATCATTTA

Cloned 5'UTRs:

CD69

ACTGAACAAGACAGCTCCAGCTACATCTCTCCGTGGACCACTTGAGAGTCGCCAGGGACC

TTGAGGGGAAAAAAATTAAAAAGG

β-Actin

CTGTCGAGTCGCGTCCACCCGCGAGCACAGCTTCTTTGCAGCTCCTTCGTTGCCGGTCCAC

ACCCGCCACCAGTTCGCC

ΔHP

ACTGAACAAGACAGCTCCAGCTACATCTCTCCGTGGAAGGGACCTTGAGGGGAAAAAAA

TTAAAAAGG

Mut-uORF

ACTGAACAAGACAGCTCCAGCTACTTCTCTCCTTGGACCACTTGAGAGTC GCCAGGGACC

TTGAGGGGAA AAAAATTAAA AAGG

Electroporation and luciferase reporter assay

Electroporation of the activated B cells were conducted following manufacturer's protocol (Lonza, Nucleofector Kit for Stimulated Mouse B cells, VVPA-1010). Briefly, purified B cells were activated with LPS and IL-4 in B cell media for 24 hours in 37°C incubator. Cells were harvested and wash once with PBS to remove residual media. In each electroporation reaction, 8-12 million B cells were re-suspended in the 200μl nucleofector solution at room temperature with 250ng plasmid for one million B cells. Program Z-001 in the Nucleofector 2b machine was selected. The electroporated cells were immediately transferred to LPS and IL-4 supplemented and 37°C-pre-warmed B cells media. This protocol achieved 70% transfection efficiency with 60% live cells at 4h post-electroporation. Upon harvest, cells were washed with PBS once, lysed in PLB solution, and subjected to dual reporter assay following manufacturer's instructions (Promega, E1960). Alternatively, harvested cells were lysed in TRIzol for RNA extraction. Total RNA was purified from TRIzol-lysed B cells using Direct-zol RNA miniprep (Zymo Research, R2052). qRT-PCR analysis of hRluc and Rluc expression was performed as described.

Supplementary References

1. Jin HY, Gonzalez-Martin A, Miletic A, Lai M, Knight S, Sabouri-Ghomi M, et al. Transfection of microRNA mimics should be used with caution. Frontiers in Genetics. 2015;6:340. doi: 10.3389/fgene.2015.00340.

2. Xiao C, Calado DP, Galler G, Thai TH, Patterson HC, Wang J, et al. MiR-150 controls B cell differentiation by targeting the transcription factor c-Myb. Cell. 2007;131(1):146-59. Epub 2007/10/10. doi: S0092-8674(07)00958-0 [pii]
10.1016/j.cell.2007.07.021. PubMed PMID: 17923094.

3. Sanuki R, Onishi A, Koike C, Muramatsu R, Watanabe S, Muranishi Y, et al. miR-124a is required for hippocampal axogenesis and retinal cone survival through Lhx2 suppression. Nat Neurosci. 2011;14(9):1125-34. Epub 2011/08/23. doi: nn.2897 [pii]
10.1038/nn.2897. PubMed PMID: 21857657.

4. Bissels U, Wild S, Tomiuk S, Holste A, Hafner M, Tuschl T, et al. Absolute quantification of microRNAs by using a universal reference. RNA. 2009;15(12):2375-84. Epub 2009/10/29. doi: rna.1754109 [pii]
10.1261/rna.1754109. PubMed PMID: 19861428; PubMed Central PMCID: PMC2779673.

5. Ingolia NT, Ghaemmaghami S, Newman JRS, Weissman JS. Genome-Wide Analysis in Vivo of Translation with Nucleotide Resolution Using Ribosome Profiling. Science. 2009;324(5924):218-23. doi: Doi 10.1126/Science.1168978. PubMed PMID: ISI:000265024400037.

6. Ingolia NT, Brar GA, Rouskin S, McGeachy AM, Weissman JS. The ribosome profiling strategy for monitoring translation in vivo by deep sequencing of ribosome-protected mRNA fragments. Nat Protoc. 2012;7(8):1534-50. Epub 2012/07/28. doi: nprot.2012.086 [pii]
10.1038/nprot.2012.086. PubMed PMID: 22836135; PubMed Central PMCID: PMC3535016.

7. Langmead B, Trapnell C, Pop M, Salzberg SL. Ultrafast and memory-efficient alignment of short DNA sequences to the human genome. Genome Biol. 2009;10(3):R25. Epub 2009/03/06. doi: gb-2009-10-3-r25 [pii]
10.1186/gb-2009-10-3-r25. PubMed PMID: 19261174; PubMed Central PMCID: PMC2690996.

8. Kim D, Pertea G, Trapnell C, Pimentel H, Kelley R, Salzberg SL. TopHat2: accurate alignment of transcriptomes in the presence of insertions, deletions and gene fusions. Genome Biol. 2013;14(4):R36. Epub 2013/04/27. doi: gb-2013-14-4-r36 [pii]
10.1186/gb-2013-14-4-r36. PubMed PMID: 23618408; PubMed Central PMCID: PMC4053844.

9. Trapnell C, Roberts A, Goff L, Pertea G, Kim D, Kelley DR, et al. Differential gene and transcript expression analysis of RNA-seq experiments with TopHat and Cufflinks. Nat Protoc. 2012;7(3):562-78. Epub 2012/03/03. doi: nprot.2012.016 [pii]
10.1038/nprot.2012.016. PubMed PMID: 22383036; PubMed Central PMCID: PMC3334321.

10. Trapnell C, Hendrickson DG, Sauvageau M, Goff L, Rinn JL, Pachter L. Differential analysis of gene regulation at transcript resolution with RNA-seq. Nat Biotechnol. 2013;31(1):46-53. Epub 2012/12/12. doi: nbt.2450 [pii]
10.1038/nbt.2450. PubMed PMID: 23222703; PubMed Central PMCID: PMC3869392.

11. Quinlan AR, Hall IM. BEDTools: a flexible suite of utilities for comparing genomic features. Bioinformatics. 2010;26(6):841-2. doi: Doi 10.1093/Bioinformatics/Btq033. PubMed PMID: ISI:000275243500019.
12. Wolfe AL, Singh K, Zhong Y, Drewe P, Rajasekhar VK, Sanghvi VR, et al. RNA G-quadruplexes cause eIF4A-dependent oncogene translation in cancer. Nature. 2014;513(7516):65-70. Epub 2014/08/01. doi: nature13485 [pii]
10.1038/nature13485. PubMed PMID: 25079319; PubMed Central PMCID: PMC4492470.
13. Gruber AR, Lorenz R, Bernhart SH, Neuboock R, Hofacker IL. The Vienna RNA Websuite. Nucleic Acids Research. 2008;36:W70-W4. doi: Doi 10.1093/Nar/Gkn188. PubMed PMID: ISI:000258142300015.
14. Xiao C, Srinivasan L, Calado DP, Patterson HC, Zhang B, Wang J, et al. Lymphoproliferative disease and autoimmunity in mice with increased miR-17-92 expression in lymphocytes. Nat Immunol. 2008;9(4):405-14. Epub 2008/03/11. doi: ni1575 [pii]
10.1038/ni1575. PubMed PMID: 18327259; PubMed Central PMCID: PMC2533767.
